# Systematic review of cash plus or bundled interventions targeting adolescents in Africa to reduce HIV risk

**DOI:** 10.1186/s12889-023-17565-9

**Published:** 2024-01-20

**Authors:** Kate Rogers, Rikke Le Kirkegaard, Joyce Wamoyi, Kaley Grooms, Shaffiq Essajee, Tia Palermo

**Affiliations:** 1 Policy Research Solutions LLC and University at Buffalo, Buffalo, NY USA; 2 United Nations Children’s Fund (UNICEF), New York, NY USA; 3 National Institute for Medical Research, Mwanza, Tanzania

**Keywords:** HIV, Adolescents, Bundled programs, Social protection, Cash plus, Africa

## Abstract

**Background:**

HIV remains a leading cause of death for adolescents and young people aged 10–24 years. HIV prevention requires multisectoral approaches that target adolescents and young people, addressing HIV risk pathways (e.g., transactional sex, gender-based violence, and school attendance) through bundled interventions that combine economic strengthening, health capabilities, and gender equality education. However, best practices are unknown because evidence on multisectoral programming targeted to adolescents and combining these components has not been systematically reviewed.

**Methods:**

We conducted a systematic review to summarize the evidence on bundled interventions combining health and economic strengthening components for adolescents and young people and their effects on HIV/STI incidence and risk factors. We included studies from Africa published between 2005 and 2023, combining at least one economic strengthening and one health component, directed toward adolescents and young people aged 10–24 years. Included studies measured programmatic impacts on primary outcomes: HIV and STI incidence/prevalence; and mediators as secondary outcomes: sexual behaviours, sexual and reproductive health, school attendance, health-seeking behaviours, and violence. We conducted key word searches in PubMed, EMBASE, and Web of Science, imported titles/abstracts from the initial search, and reviewed them using the inclusion criteria. Full texts of selected articles were reviewed and information was extracted for analysis. Findings from the full texts identified were summarized.

**Results:**

We reviewed 58 studies, including 43 quantitative studies and 15 qualitative studies, evaluating 26 unique interventions. A majority of studies reviewed were conducted in Eastern and Southern Africa. Interventions reviewed showed a greater number of significant results in improving economic outcomes; mental health and psychosocial outcomes; sexual and reproductive health knowledge and services utilization; and HIV prevention knowledge and testing. They showed fewer significant results in improving outcomes related to HIV incidence/prevalence; sexual risk behaviours; gender-based violence; gender attitudes; education; STI incidence, prevalence and testing; and sexual debut.

**Conclusions:**

Our review demonstrated the potential for bundled, multisectoral interventions for preventing HIV and facilitating safe transitions to adulthood. Findings have implications for designing HIV sensitive programmes on a larger scale, including how interventions may need to address multiple strata of the social ecological model to achieve success in the prevention of HIV and related pathways.

**Supplementary Information:**

The online version contains supplementary material available at 10.1186/s12889-023-17565-9.

## Introduction

Progress against HIV and in support of broader adolescent well-being in general has slowed recently, in part due to the COVID-19 pandemic [1]. The 2020 targets for new HIV infections and AIDS-related deaths were missed, and no region achieved 90–90-90 testing, treatment and viral suppression targets [1]. Moreover, HIV remains a leading cause of death for adolescents and young people aged 10–24 years [2]. Recognizing the role of poverty and inequality in impeding progress, social protection has gained increasing traction as a tool in the prevention and treatment of HIV/AIDS [3]. This is evidenced by the recently adopted Global AIDS Strategy 2021–2026, which underscores social protection as a key programmatic area. Social protection, defined as “the set of policies and programs aimed at preventing or protecting all people against poverty, vulnerability and social exclusion throughout their lifecycle, with a particular emphasis towards vulnerable groups [4],” covers an estimated 46.9% of the global population. In Africa, 17.4% of people are covered by at least one social protection benefit [5]. Cash transfers, which are regular cash payments to households with objectives related to poverty reduction and promoting investment in health and education, are a widespread social protection tool, implemented in almost all African countries [6]. Examples of large-scale, government-run cash transfers in the region include South Africa’s Child Support Grant, Zambia’s Social Cash Transfer, and Kenya’s Cash Transfer for Orphans and Vulnerable Children, among others. Given widespread coverage, there is potential for reaching large populations with HIV prevention and treatment efforts through integrated social protection. However, while some social protection programmes operating at scale have addressed HIV vulnerabilities through their targeting or through spillover and secondary effects [7], other HIV-sensitive social protection programmes have been largely implemented as demonstration models, without systematic integration into social protection systems. These HIV-sensitive social protection programmes are defined as social protection programming which addresses risk, vulnerability, or impact of HIV/AIDS [8]. Some may be specifically targeted to households with adolescents, while others may be household-targeted but have indirect, protective effects on HIV risk factors among adolescents.

Despite the recent global expansion of social protection programming, adolescents and children have not benefitted from this programming proportionately [9]. This may indicate a missed opportunity in HIV prevention efforts. Different groups, including adolescents, girls, and women, experience poverty and deprivation differently [10]. Moreover, many of the risk factors for HIV infection, such as dropping out of school, early marriage and pregnancy, risky sexual behavior, and experience of gender-based violence, become heightened during adolescence [11, 12]. Thus, social protection must be sufficiently age-sensitive and gender-responsive in order to be fully HIV-sensitive.

Direct impacts of social protection, including cash transfers, on HIV prevalence and incidence have not been widely evaluated [13]; however one study compared population-based data on HIV prevalence with coverage rates of national cash transfer programmes and concluded that cash transfers were associated with a reduction in new HIV infections [14]. Despite this limited evidence on social protection’s impacts on HIV incidence and prevalence, positive impacts of social protection have been found on various protective mediators such as increased school attendance, food security, and violence reduction [15–17]. Among adolescents in particular, cash transfers improve health and well-being [9], including reducing HIV risk factors such as transactional sex, number of sexual partners, and delaying sexual debut [18–21]. The evidence on cash transfers and their direct impact on reducing HIV incidence among adolescents is more limited and mixed [21–23], and does not come from government programmes at scale.

Other, related intersectoral approaches have shown potential for positive impacts across several mediators of HIV infection, particularly among adolescents and young people [8]. Some intersectoral approaches comprising cash transfers combined with complementary programming or linkages to services are referred to as “cash plus” [24], or integrated social protection programmes. By combining cash transfers with additional programmes and services that address the risks and vulnerabilities experienced by adolescents, social protection can become promotive (enhancing income and capabilities) or even transformational (addressing power relations, equity, and exclusion) [25]. In turn, individuals’ inclusion in socioeconomic activities and their capabilities can be strengthened, which may ultimately reduce their risk of HIV, or alternatively, increase their ability to access and adhere to treatment. Intersectoral programmes targeted to adolescents typically involve various combinations of economic strengthening (e.g., cash transfers) with life skills, information, or linkages to health services. Such interventions have comprised components such as economic strengthening in the form of savings accounts, cash transfers, productive grants, and livelihoods training, combined with training on gender and reproductive health (often with linkages to health services), mentoring, and/or safe spaces. Based on these combinations, we refer to these intersectoral programmes as “bundled programmes,” given that not all of them have cash as their economic strengthening component. Bundled interventions for adolescents can help overcome barriers to safe transitions to adulthood, including HIV risk. These types of multicomponent interventions recognize that poverty shapes vulnerability to HIV [26], and that the economic and reproductive health challenges that adolescent girls, in particular, face are closely linked [10]. This bundled programming may also result in synergistic impacts above and beyond isolated effects of single-sector interventions.

While these interventions are often implemented by non-governmental organizations or researchers and not linked to social protection systems, they mirror how an integrated social protection programme working across sectors may influence HIV risk, addressing both economic strengthening and health capabilities, and thus findings may be informative for developing and scaling up HIV sensitive social protection programmes. Nevertheless, the evidence on these programmes is mixed and often context-specific, and many new studies have emerged in the past few years. To date, the effects of these bundled interventions for adolescents and young people on HIV and related risk factors has not been synthesized, and thus knowledge on what outcomes they improve and the most effective bundles remains elusive. Related reviews have been conducted on cash transfers for HIV prevention [13, 27], HIV-sensitive social protection for young women [8], structural interventions for gender equality and livelihoods [28], economic interventions to prevent IPV and HIV risk behaviours [26], social safety nets and childhood violence [17], cash transfers and IPV [29, 30], and social safety nets and adolescent well-being [9]. One scoping review was also conducted on implementation science for the prevention and treatment of HIV among adolescents and young people in Africa [31], and another scoping review focused on theory-based interventions which address multi-behavioural domains for young people [32]. While some of these reviews do focus on adolescents and young people [8, 9, 28, 33], ultimately none of them focused on bundled interventions as we define them: to simultaneously strengthen economic security and health/life skills.

In the current study, we conduct a systematic review to answer the question, how do bundled interventions which jointly aim to strengthen economic and health or life skills capabilities among adolescents and young people improve outcomes related to HIV risk in Africa? We focus on Africa specifically given the HIV burden in the region, where two out of three new infections are among adolescent girls and young women (AGYW) [34], combined with the availability of evidence on bundled programmes targeting socioeconomic and gendered vulnerabilities and risk factors in the region. We define economic strengthening broadly: the programmes we examine encompass cash transfers, productive grants, savings accounts, financial literacy training, income-generating activities, and livelihoods training.

### Conceptual framework

**Fig. 1:**
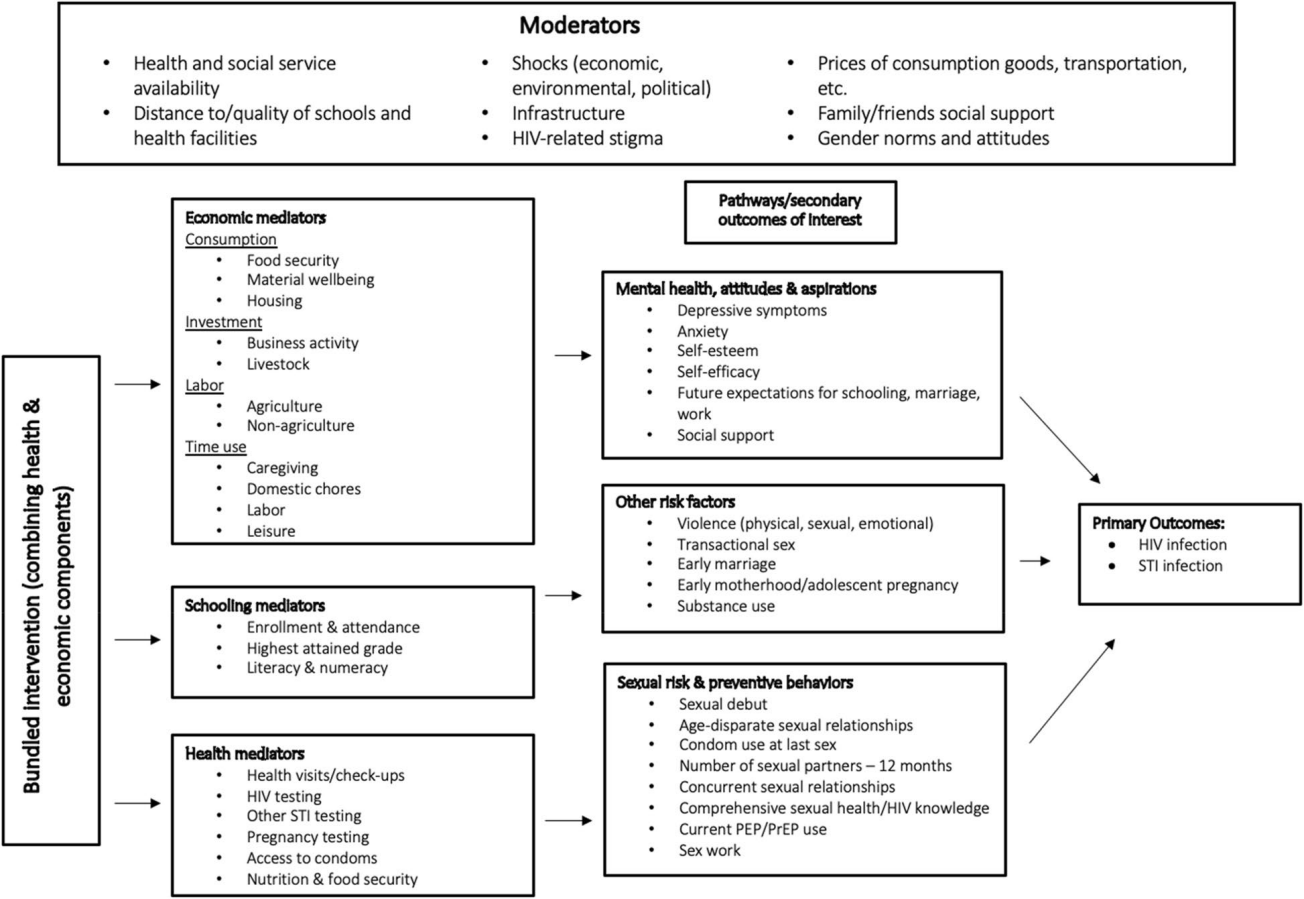
Conceptual Framework

The primary outcomes of interest in our review are HIV and STI infection, and as secondary outcomes we examine pathways and risk factors (mediators), which are laid out in the Conceptual Framework (Fig. 1). This framework illustrates the complex interplay of structural, community, household, relationship, and individual drivers influencing HIV and STI infection among adolescents (see Appendix 2 for details). By integrating bundled or “cash plus” approaches, programmes and interventions can tackle gendered vulnerabilities, provide economic empowerment, and address multiple other health and social risks.

### Definitions

Before describing our search process, we first provide some key definitions. Adolescence comprises a life phase of both biological growth and transitions in major social roles and is often defined as the period from 10 to 19 years [35]. Nevertheless, some have argued that 10–24 years corresponds more accurately to adolescent growth and reflects social aspects of the transition to adulthood [35]. In the current study, we focus on adolescents and young people as reflective of definitions for the age groups identified by the United Nations (ages 10–24 years). This age range also encompasses the target group (i.e., girls and young women aged 15–24 years) for increased investments identified by the Determined, Resilient, Empowered, AIDS-free, Mentored and Safe (DREAMS) partnership, based on increased risk of infection among this age group, particularly girls.

## Methods

### Search process

We conducted a systematic review informed by the PI[C]O framework. The interventions we sought to review comprised bundled interventions which include both 1) an economic (in-kind donations, livelihoods training, cash, voucher, or asset transfer); and 2) a health or life skills component (health information or training, psychosocial support, behavior change communication, self-efficacy training, voucher for services, safe spaces, information on gender-based violence prevention, parenting skills) and were implemented in Africa. In-kind economic strengthening included school fees and school kit donations. These could include standalone interventions targeted to adolescents or interventions targeted to adolescents but implemented as part of larger integrated social protection programmes (sometimes referred to as “cash plus”). Programmes with household-targeted cash transfers which provide additional programming targeted to adolescents were considered. Only studies with interventions implemented in Africa that adhered to the following criteria were included: 1) targeted young people ages 10–24 and 2) examined impacts of an intervention with both a health/life skills and economic component and 3) examined impacts on our primary or secondary outcomes.

The studies we included spanned various study designs, including experimental (randomized or cluster randomized control trials) and quasi-experimental (interrupted time series, matching, etc.) studies which include some sort of comparison (pre/post- test, comparison/control group) group, as well as qualitative studies. Qualitative studies were not restricted to those with a comparison group.

To select outcomes of interest, we first conducted a scoping exercise, comprised of a rapid assessment of the known literature, hand searched articles, and articles obtained from search engines (Google Scholar, PubMed). Prior to finalizing the search protocol, we convened a workshop of experts with prior experience on bundled interventions and adolescents (including academics from Eastern Africa, as well as United Nations staff from the United Nations Children’s Fund (UNICEF), the World Health Organization (WHO), the World Food Programme (WFP), and the Joint United Nations Programme on HIV/AIDS (UNAIDS)) in March 2022. The workshop helped to identify key literature, select mediators of interest, and narrowed the focus to HIV prevention (instead of prevention and adherence to treatment). Thus, any studies that focused on HIV-positive adolescents and youth and examined outcomes related to adherence were excluded from this review.

After finalizing the primary and secondary outcomes of interest (described in more detail below), we registered the search protocol with the International prospective register of systematic reviews (PROSPERO; registration ID: CRD42022325270).

We sought to include studies from 2005 through 2023. Studies before 2005 were not included, as the type of bundled capabilities strengthening interventions we aim to summarize were largely not conducted before that time. We did not conduct a meta-analysis because the interventions under review were not adequately homogeneous in terms of intervention components nor outcomes examined to do so.

### Outcomes

Our primary outcomes of interest were HIV incidence/prevalence and STI incidence/prevalence. Secondary outcomes included risk factors (mediators) and were determined based on findings from previous research and reviews [13, 27], as well as consultation with an advisory board of experts in the field of HIV and social protection. These included: educational attainment, school enrolment and attendance; food security; health visits; HIV testing; STI testing; pregnancy testing; access to condoms; mental health (depressive symptoms, anxiety); self-esteem and self-efficacy; future expectations for work, livelihood activities, savings, and wealth creation-related knowledge, schooling, and marriage; physical, sexual violence, or emotional violence; transactional sex; age disparate sex (10 years or more difference in age between partners); early marriage (defined as before 18 years); early motherhood or pregnancy (defined as before 18 years); substance use (illicit drugs and alcohol); sexual debut; condom use at last sex; number of sexual partners (past 12 months); concurrent sexual relationships; comprehensive knowledge on HIV prevention; comprehensive knowledge on modern contraceptives and access to sexual and reproductive health services; engagement in sex work; and gender attitudes. (See Appendix 3 for details.)

The search was first conducted on 15 April, 2022 and then again on 25 September, 2023 using a key word search based on findings from the inception workshop and recommendations from experts in the field (see Appendix 1). The search was conducted using search engines PubMed, EMBASE, and Web of Science. Titles and abstracts from selected articles (based on criteria above) were imported from each database and screened by two members of the research team. Conflicts for inclusion/exclusion of articles were resolved through a third-party researcher, using the inclusion criteria listed above. The remaining articles were reviewed in full-text by two reviewers. Two members of the research team and two research assistants then extracted pertinent information from all studies, including study population, timeline, location, type of intervention, research design, analysis, and results (Table 1, and Appendices 4 and 5).

**Table 1:** Panel A. Summary of programmes

**Adolescent Girls Empowerment Program (AGEP)**								
#	Author, year	Target Population	Location	Population: Age & Sex	Intervention Name	Intervention: Economic Component	Intervention: Life skills, Health, Behavioural, or Other Component	Duration of Intervention
1	Austrian, Soler-Hampejsek, Behrman, et al., 2020	Adolescent girls 10–19 in urban and rural Zambia	Zambia	Adolescent girls 10–19 in urban and rural Zambia	Adolescent Girls Empowerment Program (AGEP)	Health voucher for general wellness and SRH services; adolescent-friendly savings account; financial education	Mentor-led girl groups (“safe spaces”) with curricula delivered about SRH, HIV, life skills and financial education	Weekly for 2 years; Arm 1 was weekly meetings; Arm 2 was weekly meetings with health voucher; Arm 3 was weekly meetings, health voucher, savings account; Arm 4 was control (no interventions)
**Adolescent Girls Initiative-Kenya (AGI-K)**								
**#**	**Author, year**	**Target Population**	**Location**	**Population: Age & Sex**	**Intervention Name**	**Intervention: Economic Component**	**Intervention: Life skills, Health, Behavioural, or Other Component**	**Duration of Intervention**
2	Austrian, Soler-Hampejsek, Kangwana, et al., 2021	Adolescent girls in urban and rural Kenya	Kenya (Nairobi and Wajir)	Adolescent girls 11–14 (at baseline) in urban and rural Kenya; must be residing in study area and not enrolled in boarding school	Adolescent Girls Initiative-Kenya (AGI-K)	Education: cash and in-kind (school fees, school kits) transfers conditional on school enrollment of the girl Wealth creation: financial education and savings and activities	Violence prevention: committees developed action plans and a proposed budget to address violence-in Kibera, new resource centers or libraries for girls; in Wajir, improving primary schools Health: health and life-skills training led by a trained female mentor	2 years
3	Austrian, Soler-Hampejsek, Kangwana, et al., 2022	11–14 year old girls + whole community was targeted for violence prevention dialogue	Kenya (Wajir)	Girls 11–14 years + community members	Adolescent Girls Initiative-Kenya (AGI-K)	CCT (condition = education) and financial literacy training on wealth creation; savings activities	Community dialogue about inequitable gender norms as violence prevention, group meetings for health and life skills curricula; weekly mentor-led group meetings covering health, life skills, financial education curricula and savings activities	2 years
4	Kangwana, Austrian, Soler-Hampejsek, 2022	AGYW	Kenya	Girls 11–14 years	Adolescent Girls Initiative - Kenya	Conditional cash transfer (education) and savings activities (wealth).	Community dialogues on unequal gender norms and their consequences (vIolence prevention), health and life skills training (health), and financial literacy training.	24 months
**Biruh Tesfa (Bright Future)**								
**#**	**Author, year**	**Target Population**	**Location**	**Population: Age & Sex**	**Intervention Name**	**Intervention: Economic Component**	**Intervention: Life skills, Health, Behavioural, or Other Component**	**Duration of Intervention**
5	Erulkar, Ferede, Girma, et al., 2013	Out-of-school girls ages 10–19 in slum areas of urban Ethiopia	urban areas in Ethiopia (Addis Ababa, Gondar, and Bahir Dar)	Adolescent girls 12–19 years	Biruh Tesfa (Bright Future)	financial literacy curriculum	Group meetings; Life skills covered self-esteem, communication, gender/power dynamics, rape, coercion, menstruation, reproductive autonomy, STI/HIV, counseling, and testing, as well as optional literacy component	30 months of implementation; curriculum is approximately 30 hours
**Bridges to the Future & BridgesPLUS**								
**#**	**Author, year**	**Target Population**	**Location**	**Population: Age & Sex**	**Intervention Name**	**Intervention: Economic Component**	**Intervention: Life skills, Health, Behavioural, or Other Component**	**Duration of Intervention**
6	Kivumbi, Byansi, Ssewamala, et al., 2019	Female adolescent orphans who lost one or bother parents to AIDS, lived within a family, and were enrolled in grad 5 or 6 of government aided primary school	Uganda	Adolescent girls 10–16 years	Bridges to the Future	Used data from Bridges to the Future Study - bolstered standard of care as well as an economic empowerment intervention comprising of a child development account and workshops on financial management and microenterprise development	Mentorship with peer mentors throughout the intervention period	Not specified
**DREAMS (to include Sauti Project)**								
**#**	**Author, year**	**Target Population**	**Location**	**Population: Age & Sex**	**Intervention Name**	**Intervention: Economic Component**	**Intervention: Life skills, Health, Behavioural, or Other Component**	**Duration of Intervention**
7	Birdthistle, Kwaro, Shahmanesh, et al., 2021	AGYW 15–24 years old	Gem, Kenya & uMkhanyakude, South Africa (DREAMs sites)	Adolescent girls and young women 15–24 years	DREAMS	DREAMS core packages include strengthening families of AGYW economically (including cash transfers or education subsidies); specific components evaluated not specified	DREAMS core packages include strengthening families of AGYW economically (including cash transfers or education subsidies); specific components evaluated not specified	Not specified
8	Birdthistle, Carter, Mthiyane, et al., 2022	AGYW 15–22years in Nairobi slum settlements; AGYW 13–22 years in rural KwaZulu-Natal	Nairobi, Kenya & rural KwaZulu-Natal, South Africa	Adolescent girls and young women 13–22 years	DREAMS	DREAMS core packages include strengthening families of AGYW economically (including cash transfers or education subsidies); specific components evaluated not specified	DREAMS core packages include strengthening families of AGYW economically (including cash transfers or education subsidies); specific components evaluated not specified	Not specified
9	Chabata, Hensen, Chiyaka, et al., 2021	Young women aged 18–24 who sell sex	Zimbabwe	Young women 18 to 24 years	DREAMS	DREAMS package was delivered through several implementing partners in the two cities; services available included social protection, life skills, education and vocational training.	DREAMS package was delivered through several implementing partners in the two cities; services available included gender-based violence prevention and care, and HIV prevention, including condom promotion and distribution, an offer of PrEP combined with community empowerment and adherence support for those at highest risk of HIV. Community-based activities aimed to increase demand for and uptake of PrEP and the DREAMS package more generally, and to support PrEP adherence.	24 months
10	Floyd, Mulwa, Magut, et al., 2022	AGYW aged 13–22	Kenya and South Africa	Adolescent girls and young women 13–22 years	DREAMS	DREAMS core packages include strengthening families of AGYW economically (including cash transfers or education subsidies); specific components evaluated not specified	DREAMS core packages include strengthening families of AGYW economically (including cash transfers or education subsidies); specific components evaluated not specified	Up to 4 years in Kenya; Up to 2 years in South Africa
11	Gourlay, Floyd, Magut, et al., 2022	AGYW 13–22 years	Kenya (Gem and Nairobi), South Africa (uMkhanyakude)	Adolescent girls and young women 13–22 years	DREAMS	DREAMS core packages include strengthening families of AGYW economically (including cash transfers or education subsidies); specific components evaluated not specified	DREAMS core packages include strengthening families of AGYW economically (including cash transfers or education subsidies); specific components evaluated not specified	2 years (2016–2018, 3 years post-DREAMS baseline implementation
12	Govender, Beckett, Reddy, et al., 2022	AGYW aged 12–22	South Africa	Adolescent girls and young women 12–22 years	DREAMS-like interventions (DREAMS and other similar interventions)	DREAMS core packages include strengthening families of AGYW economically (including cash transfers or education subsidies); specific components evaluated not specified	DREAMS core packages include strengthening families of AGYW economically (including cash transfers or education subsidies); specific components evaluated not specified	1 year
13	Kuringe, Christensen, Materu, et al., 2022	Out-of-school AGYW	Tanzania	Adolescent girls and young women 15–23 years	DREAMS (Sauti project)	cash transfer quarterly	10-hour sessions of social and behavior change communication	Not specified
14	Mathur, Heck, Kishor Patel, et al., 2022	AGYW enrolled in DREAMS	Kenya, Malawi & Zambia	Adolescent girls and young women 10–24 years	DREAMS	DREAMS core packages include strengthening families of AGYW economically (including cash transfers or education subsidies); specific components evaluated not specified	DREAMS core packages include strengthening families of AGYW economically (including cash transfers or education subsidies); specific components evaluated not specified	24 months
15	Mthiyane, Baisley, Chimbindi et al., 2022	AGYW aged 13–22	South Africa (rural)	Adolescent girls and young women 13–22 years	DREAMS	DREAMS core packages include strengthening families of AGYW economically (including cash transfers or education subsidies); specific components evaluated not specified	DREAMS core packages include strengthening families of AGYW economically (including cash transfers or education subsidies); specific components evaluated not specified	Not specified
16	Mulwa, Osindo, Wambiya, et al., 2021	AGYW participating in DREAMS	Kenya	Adolescent girls and young women 15–22 years	DREAMS	DREAMS core packages include strengthening families of AGYW economically (including cash transfers or education subsidies); specific components evaluated not specified	DREAMS core packages include strengthening families of AGYW economically (including cash transfers or education subsidies); specific components evaluated not specified	24 months
17	Pelletier, Derado, Maoela, et al., 2022	Pregnant AGYW attending antenatal clinics	Lesotho	Adolescent girls and young women 15–24 years	DREAMS	DREAMS core packages include strengthening families of AGYW economically (including cash transfers or education subsidies); specific components evaluated not specified	DREAMS core packages include strengthening families of AGYW economically (including cash transfers or education subsidies); specific components evaluated not specified	4 years
18	Van Heerden, Sausi, Oliver, et al., 2020	AGYW & their caregivers - in DREAMS and not	Lesotho	Adolescent girls and young women 10–24 years + their caregivers	DREAMS	Social asset building: internal lending communities (savings-led microfinance); financial education and entrepreneurship training; job placement. Caregiver intervention: opportunity to participate in internal lending community.	Adolescent-friendly health services, referrals & linkages to services, community service provision days (events to make community members aware of services). Caregiver intervention focused on capacity building for caregivers, building parenting skills.	2 years (in Lesotho - began in 2015); study took place 8 months after onset of intervention
19	Wambiya, Gourlay, Mulwa, et al., 2023	AGYW aged 13–22	Kenya and South Africa	Adolescent girls and young women 13–22 years	DREAMS	DREAMS core packages include strengthening families of AGYW economically (including cash transfers or education subsidies); specific components evaluated not specified	DREAMS core packages include strengthening families of AGYW economically (including cash transfers or education subsidies); specific components evaluated not specified	Not specified
**Empowerment and Livelihood for Adolescents (ELA)**								
**#**	**Author, year**	**Target Population**	**Location**	**Population: Age & Sex**	**Intervention Name**	**Intervention: Economic Component**	**Intervention: Life skills, Health, Behavioural, or Other Component**	**Duration of Intervention**
20	Bandiera, Buehren, Burgess, et al., 2020	Adolescent girls 14–20 participating in BRAC clubs in urban and rural Uganda	Uganda (urban and rural settings)	Adolescent girls 14–20 years	Empowerment and Livelihood for Adolescents (ELA)	vocational skills training through a series of courses on income-generating activities (e.g., hairdressing, computing, etc.) with focus on establishing small-scale enterprises. Supplemented with financial literacy courses (budgeting, etc.). Two years after intervention started, limited microfinance was offered to girls in half of treatment areas (very low take-up).	life skills (given by community mentors close in age or BRAC staff) and include courses on SRH, menstruation, pregnancy, STIs, HIV awareness, family planning, and rape; also include conflict management and negotiation skills along with practical legal knowledge (e.g., bride price, child marriage, and VAW).	2 years
21	Buehren, Goldstein, Gulesci, et al., 2017	AGYW	Tanzania	Adolescent girls (age range not specified)	Empowerment and Livelihood for Adolescents (ELA)	livelihoods training (education on IGAs), financial education. Microcredit services (provided to half of the girls’ clubs - only older adolescents) & financial literacy training	adolescent development centers (girls clubs, meant to be safe spaces), life-skills training (SRH, family planning, etc.), and sensitization meetings with the parents and village elders	Approximately 1–1.5 years (not clearly specified)
**Girl Empower**								
**#**	**Author, year**	**Target Population**	**Location**	**Population: Age & Sex**	**Intervention Name**	**Intervention: Economic Component**	**Intervention: Life skills, Health, Behavioural, or Other Component**	**Duration of Intervention**
22	Özler, Hallman, Guimond, et al., 2020	Adolescent girls 13–14 years	Liberia	Adolescent girls 13–14 years	Girl Empower	**GE:** cash to start savings account, a savings book and a cash box ($2 per month for a total of $16 during the eight-month implementation).**GE+:** caregivers of program participants received of a payment of $1.25 for each of the 32 regular sessions that the adolescent girl attended (maximum $40).	GE & GE+: weekly meetings with female mentors, aged 20 to 35, for a total of 39 weeks in safe spaces designated by community. 2 mentors per group (130 mentors in total) to facilitate 32 weekly sessions based on a life skills curriculum, covering: Sense of self; Feelings and emotions; Social networks; Protection and safety; Financial literacy; Reproductive Health; Leadership and Empowerment; and Setting life goals. Additional 7 weeks of meetings to prepare community action event & graduation ceremonies. Caregivers: 8 monthly facilitated sessions to reinforce content & encourage protection of girls in their communities.	46 weeks
**The SHAZ (Shaping the Health of Adolescents in Zimbabwe) Project**								
**#**	**Author, year**	**Target Population**	**Location**	**Population: Age & Sex**	**Intervention Name**	**Intervention: Economic Component**	**Intervention: Life skills, Health, Behavioural, or Other Component**	**Duration of Intervention**
23	Dunbar, Maternowska, Kang, et al., 2010	Adolescent female orphans in semi-urban Zimbabwe	Zimbabwe	Orphaned adolescent girls and young women < 20 years	SHAZ!	Access to microcredit loans ($51–87 USD), business training/mentoring, skill-building (e.g., making soap) workshops. Loan repayment in 3–9 months at 30% interest.	Life-skills education - HIV, SRH knowledge and skills, and issues related to gender, culture, physical/sexual violence	6 months: Life skills were 10 sessions; Business training was 4 days.
24	Dunbar, Kang Dufour, Lambdin, et al., 2014	Adolescent female orphans (16–19 y/o) out of school, HIV-uninfected, not pregnant, living in high-density urban area	Zimbabwe (Chitungwiza)	Adolescent girls and young women 16–19 years	SHAZ!	Vocational training, guidance counseling and a micro-grant (100 USD) and financial literacy education	HIV and sexual and reproductive health screenings; conducted life skills curriculum (Drawing on Stepping Stones and CDC Zimbabwe Talk Time) which included HIV/STI and reproductive health; relationship negotiation; strategies to avoid violence; and identification of safe and risky places in the community; and home-based care training	6 months (on average)
**Suubi**								
**#**	**Author, year**	**Target Population**	**Location**	**Population: Age & Sex**	**Intervention Name**	**Intervention: Economic Component**	**Intervention: Life skills, Health, Behavioural, or Other Component**	**Duration of Intervention**
25	Curley, Ssewamala, Nabunya, et al., 2016	AIDS-orphans (lost one or more parents due to AIDS) in Uganda between ages 11 and 17	Uganda	Adolescent girls 11–17 years	Suubi	Family based economic component: workshops that focus on financial education, asset building, and career planning; 2) mentorship from near-peers to reinforce learning; and 3) a joint CDA in both the child’s and caregiver’s name	Monthly mentorship (received by treatment only), support and counseling from faith-based organizations in the target community plus school supplies (received by treatment & controls)	Not specified
26	Ssewamala, Ismayilova, McKay, et al., 2010	Adolescents who lost 1+ parents from AIDS & were enrolled in school	Uganda	Male and female adolescents ~ 13 years	SUUBI	Matched CSA and 12 1–2-hour workshops over a 10-month period focused on asset building and financial planning, including topics related to asset-building strategies, including saving, education, and small business development	Standard of care: counseling and educational related supplies (including textbooks), health education (including AIDS-focused education) provided through a nationwide school-based curriculum, monthly mentoring on future planning and life options	10 months
27	Ssewamala, Nielands, Waldfogel, 2012	Adolescent orphans, having lost one or both parents to HIV/AIDS, enrolled in the last 2 years of primary school	Uganda	Male and female adolescents 10–16 years	SUUBI	Comprehensive microfinance intervention comprising matched savings accounts, financial management workshops & small business classes	Standard of care: counseling and educational related supplies (including textbooks), health education (including AIDS-focused education) provided through a nationwide school-based curriculum, monthly mentoring on future planning and life options	5 years (2012–2017)
28	Ssewamala, Brathwaite, Neilands, et al., 2023	Adolescent girls	Uganda	Adolescent girls 14–17 years	Suubi	1-to-1 matched savings youth development account (YDA)	Evidence-based family strengthening intervention designed to enhance youth behavioral health	2 years
**Suubi-Maka Project**								
**#**	**Author, year**	**Target Population**	**Location**	**Population: Age & Sex**	**Intervention Name**	**Intervention: Economic Component**	**Intervention: Life skills, Health, Behavioural, or Other Component**	**Duration of Intervention**
29	Jennings, Ssewamala, Nabunya, 2016	Adolescent orphans (lost one or bother parents to AIDS) in the last 2 years of primary schooling & were living within a family, & their caregivers	Uganda	Male and female adolescents 10–17 years + their caregivers	Suubi-Maka Project	Financial education, and a matched CSA held in the adolescent orphan’s name (match limit equivalent to US$10 a month), financial education & mentoring	Usual orphan care services (counseling services, school lunch, and scholastic materials (textbooks and uniforms)) plus monthly mentoring (both control and treatment groups received orphan care + mentoring)	12 months
30	Karimli & Ssewamala 2015	Adolescent orphans, having lost one or both parents to HIV/AIDS, enrolled in the last 2 years of primary school, and living within a family setting	Uganda	Adolescent girls < 18 years	Suubi-Maka Project	Suubi-Maka: [1] a matched savings account (Child Savings Account – CSA) held in the adolescent’s name with his/her parent/guardian as a co-signatory in a recognized financial institution. Any of the adolescent’s family members, relatives, or friends were allowed to contribute towards the CSA. The account was then matched with money from the intervention program. The match cap was an equivalent of US$10 a month per family or US$200 for the 20-month intervention period. The match rate was 2:1. Participant & their guardians attended four training sessions on financial management covering microenterprise development principles, working with financial institutions, savings and investment, and goal-specific training focused on particular businesses (e.g., chicken rearing).	Suubi-Maka: control group received enhanced usual care for orphans in the study region, which consisted of counseling, food aid (school lunches), scholastic materials (textbooks and notebooks), and mentorship.	20 months
31	Ssewamala, Karimli, Torsten, et al., 2016	Adolescents orphaned by AIDS in the last 2 years of primary school	Uganda	Male and female adolescents 12–16 years	Suubi-Maka Project	Family-level economic strengthening intervention in the form of a matched Child Savings Account (Suubi-Maka treatment arm) - matched by up to $10USD/family/month for 12 months. Participants also received 10 1–2 hour microenterprise development workshops on starting family-based income-generating activities & financial management (including how to save money)	Standard of care (counseling, school uniforms, school lunch, notebooks, and textbooks), “bolstered” with mentorship from a near-peer	1 mentorship meeting/month for 12 months
32	Tutlam, Filiatreau, Byansi, et al., 2023	AIDS-orphaned adolescents	Uganda	Male and female adolescents 12–16 years	Suubi Maka	Family-level economic strengthening intervention: family-level income-generating projects (micro-enterprises) believed to enhance economic stability, reduce poverty, and enhance protective family processes for youth orphaned by AIDS; and monetary savings for educational opportunities for AIDS-orphaned children; matched savings account of $10/month	Adult mentors to children	Not specified
**Suubi4Her**								
**#**	**Author, year**	**Target Population**	**Location**	**Population: Age & Sex**	**Intervention Name**	**Intervention: Economic Component**	**Intervention: Life skills, Health, Behavioural, or Other Component**	**Duration of Intervention**
33	Filiatreau, Tutlam, Brathwaite, et al., 2023	AGYW	Uganda	Adolescent girls 14–17 years	Suubi4Her	Bank account open in name of participant and 1:1 matched savings program	Provided a safe setting for multiple families to gatherand directly discuss family challenges, shared experiences, adolescent mental health challenges, and potential strategies formitigating these challenges.	12 months
**Women First and Go Girls!**								
**#**	**Author, year**	**Target Population**	**Location**	**Population: Age & Sex**	**Intervention Name**	**Intervention: Economic Component**	**Intervention: Life skills, Health, Behavioural, or Other Component**	**Duration of Intervention**
34	Burke, Field, González-Calvo, et al., 2019	13–19-year-old girls (13–17 years at intervention start)	Mozambique	Adolescent girls and young women 13–19 years	Women First and Go Girls!	Women First, using Go Girls! curriculum: trains women to sell products door-to-door in their communities. Included business education followed by the sale of items included in a business “kit”. Participants were expected to re-pay the program for the kits with a portion of their sales to receive the next kit, with the remainder of their revenue considered profit to be spent or saved. Also included accumulated savings and credit associations to encourage saving.	Used the full Go Girls! curriculum and a locally-tailored gender-based violence (GBV) curriculum to encourage social empowerment and reduce adolescent girl participants’ vulnerability to HIV. The intervention also had the goal of encouraging girls to stay in school.	5 years (but start dates and implementation methods varied across communities due to logistic constraints)
**Women of Worth (cash plus)**								
**#**	**Author, year**	**Target Population**	**Location**	**Population: Age & Sex**	**Intervention Name**	**Intervention: Economic Component**	**Intervention: Life skills, Health, Behavioural, or Other Component**	**Duration of Intervention**
35	Naledi, Little, Pike, et al., 2022	AGYW 19–24 years	Cape Town, South Africa	Adolescent girls and young women 19–24 years	Women of Worth (cash plus)	Cash transfer of R300 ($22) paid after attendance at each session	“Care” interventions: [1] 12 facilitator-led, group skills building sessions to address a range of SRH/HIV determinants, [2] support services, including psychosocial services and [3] fixed (government) and mobile (non-governmental) YFHS with the promotion of HIV testing, contraception services, antiretroviral treatment and HIV pre-exposure prophylaxis referral.	12 sessions, 1:45 min each, to be completed anywhere from 10 weeks to 12 months. (Total duration 18 months - May 2017-December 2019)
**Ujana Salama: Cash Plus Model for Safe Transitions to a Healthy and Productive Adulthood**								
**#**	**Author, year**	**Target Population**	**Location**	**Population: Age & Sex**	**Intervention Name**	**Intervention: Economic Component**	**Intervention: Life skills, Health, Behavioural, or Other Component**	**Duration of Intervention**
36	Chzhen, Prencipe, Eataama, et al., 2021	Adolescents aged 14–19 at baseline living in households receiving government cash transfer	Tanzania	Male and female adolescents 14–19 years	Ujana Salama: Cash Plus Model for Safe Transitions to a Healthy and Productive Adulthood	Livelihoods training (12 weeks), 9 months of mentoring, and productive grant (80 USD)	Life-skills training (including HIV prevention and treatment, gender, violence; 12 weeks) and supply-side strengthening of adolescent friendly HIV and SRH servicesnd linkages to existing SRH and HIV services for adolescents	2-hour weekly sessions over a 12-week period for livelihoods and life skills training and then mentoring phase over 9 months (2x per month)
37	Palermo, Prencipe and Kajula, 2021	Adolescents aged 14–19 at baseline living in households receiving government cash transfer	Tanzania	Male and female adolescents 14–19 years	Ujana Salama: Cash Plus Model for Safe Transitions to a Healthy and Productive Adulthood	Livelihoods training (12 weeks), 9 months of mentoring, and productive grant (80 USD)	Life-skills training (including HIV prevention and treatment, gender, violence; 12 weeks) and supply-side strengthening of adolescent friendly HIV and SRH services and linkages to existing SRH and HIV services for adolescents	2-hour weekly sessions over a 12-week period for livelihoods and life skills training and then mentoring phase over 9 months (2x per month)
38	Prencipe, Houweling, van Lenthe, et al., 2022	Adolescents aged 14–19 at baseline living in households receiving government cash transfer	Tanzania	Male and female adolescents 14–19 years	Ujana Salama: Cash Plus Model for Safe Transitions to a Healthy and Productive Adulthood	Livelihoods training (12 weeks), 9 months of mentoring, and productive grant (80 USD)	Life-skills training (including HIV prevention and treatment, gender, violence; 12 weeks) and supply-side strengthening of adolescent friendly HIV and SRH services and linkages to existing SRH and HIV services for adolescents	2-hour weekly sessions over a 12-week period for livelihoods and life skills training and then mentoring phase over 9 months (2x per month)
39	Ranganathan, Quinones, Palermo et al., 2022	Adolescents aged 14–19 at baseline living in households receiving government cash transfer	Tanzania	Male and female adolescents 14–19 years	Ujana Salama: Cash Plus Model for Safe Transitions to a Healthy and Productive Adulthood	Livelihoods training (12 weeks), 9 months of mentoring, and productive grant (80 USD)	Life-skills training (including HIV prevention and treatment, gender, violence; 12 weeks) and supply-side strengthening of adolescent friendly HIV and SRH service and linkages to existing SRH and HIV services for adolescents	2-hour weekly sessions over a 12-week period for livelihoods and life skills training and then mentoring phase over 9 months (2x per month)
40	Waidler, Gilbert, Mulokozi, et al., 2022	Adolescents aged 14–19 at baseline living in households receiving government cash transfer	Tanzania	Male and female adolescents 14–19 years	Ujana Salama: Cash Plus Model for Safe Transitions to a Healthy and Productive Adulthood	Livelihoods training (12 weeks), 9 months of mentoring, and productive grant (80 USD)	Life-skills training (including HIV prevention and treatment, gender, violence; 12 weeks) and supply-side strengthening of adolescent friendly HIV and SRH service and linkages to existing SRH and HIV services for adolescents	2-hour weekly sessions over a 12-week period for livelihoods and life skills training and then mentoring phase over 9 months (2x per month)
**Unnamed Intervention**								
**#**	**Author, year**	**Target Population**	**Location**	**Population: Age & Sex**	**Intervention Name**	**Intervention: Economic Component**	**Intervention: Life skills, Health, Behavioural, or Other Component**	**Duration of Intervention**
41	Austrian & Muthengi, 2014	Adolescent girls 10–19 in low-income areas of Kampala, Uganda	Uganda	Adolescent girls and young women 10–19 years in low-income areas of Kampala, Uganda	None specified	1) financial education + savings accounts and 2) savings account only	Safe spaces group meetings with reproductive health education and social asset building (with community mentors 20–35 y/o)	Safe spaces mentorship was weekly for 30–90 minutes; reproductive health lessons were 30 sessions; lasted 12 months
42	Hegdahl, Musonda, Svanemyr, et al., 2022	Adolescent girls in grade 7	Zambia (rural)	Adolescent girls in Grade 7	None specified	Month cash transfer to the girls, yearly cash transfer to their parents/guardians, school fee coverage	Six community and parent meetings per year on the benefits of girls’ education and postponement of early marriage and child bearing; and youth clubs every second week (36 in total) providing CSE for the participants and boys in the same class	2 years
43	Tozan, Capasso, Sun, et al., 2019	Adolescents orphaned by AIDS	Uganda	Male and female adolescents 10–16 years	None specified	Incentivized savings account [Child Development Account (CDA)] with either a 1:1 match rate (Bridges) or 2:1 match rate (BridgesPLUS). All participants in Bridges and BridgesPLUS received: three sessions on financial literacy and management (FLT), including how to save, budget and support asset accumulation & six sessions on income generating activities	Standard of care for OVC (counseling by community priests and school supplies) plus eight sessions of peer mentorship	5 years (2012–2016)
**Panel B. Summary of Programmes:** ***Qualitative Studies***								
**DREAMS (to include Sauti Project)**								
**#**	**Author, year**	**Target Population**	**Location**	**Population: Age & Sex**	**Intervention Name**	**Intervention: Economic Component**	**Intervention: Life skills, Health, Behavioural, or Other Component**	**Duration of Intervention**
1	Chimwaza-Manda, Kamndaya, Pilgrim, et al., 2023	Very Young Adolescents (VYA)	Malawi	Adolescent girls 10–14 years: one sample participating in DREAMS Girls Only Clubs (*N* = 23) and comparative sample was girls who were not in clubs, in region where DREAMS was not implemented (*n* = 20)	DREAMS: Girls Only Club	DREAMS core packages includes strengthening families of AGYW economically (including cash transfers or education subsidies); specific components evaluated not specified	Multisectoral package of interventions including strengthening existing HIV testing, prevention, and linkage to care interventions and the introduction of evidence-based interventions for gender-based violence, family and caregiving, social asset building; specific components evaluated in this study not specified	Not specified
2	Gangaramany, Balvanz, Gichane, et al., 2021	AGYW 15–23 years, their influencers (mothers & partners), and financially empowered women 20–30 years	Tanzania	Adolescent girls and young women 15–23 years, their influencers (mothers and partners), and financially empowered women 20–30 years	DREAMS (Sauti Project)	Sauti Project WORTH+ intervention: includes entrepreneurial training, mentorship, and savings and loan groups to equip women with the necessary skills to plan their economic development more efficiently and cash transfers (SIM card, $31 USD/3 months for 18 months.	Sauti Project standard intervention package: community-based HIV testing and counseling; behavioural interventions, including peer-led education sessions to promote health-seeking behaviors by improving negotiation, self-efficacy, and condom-use skills and BCC	18 months; cash every 3 months, 10 hours of BCC education
3	Gichane, Wamoyi, Atkins, et al., 2020	Out-of-school adolescent girls and young women	Tanzania	Adolescent girls and young women 15–23 years	DREAMS (Sauti Project Worth+)	Sauti Project WORTH+ intervention: includes entrepreneurial training, mentorship, and savings and loan groups to equip women with the necessary skills to plan their economic development more efficiently and cash transfers (SIM card, $31 USD/3 months for 18 months.	Behaviour change and communication group peer-led sessions that discussed topics such as TIV AND STI prevention, gender-based violence, family planning, negotiation skills, self-efficacy/agency skills, condom skills and health seeking.	10 hours BCC training; cash transfer every 3 months for 18 months
4	Manda, Pilgrim, Kamndaya, et al., 2021	Adolescent girls participating in girls’ clubs	Malawi	Adolescent girls 12–14 years	DREAMS	DREAMS project/girls club with topics related to socioeconomic approaches for caregivers, food security and nutrition, back to school support	DREAMS project/ girls club with topics related to social asset building, HIV testing, condom information, screening for case management, post violence care, access to contraceptive information & services	24 months
5	Pettifor, Wamoyi, Balvanz, et al., 2019	Out-of-school adolescent girls and young women enrolled in DREAMS	Tanzania	Adolescent girls and young women 15–23 years	DREAMS (Sauti Worth+)	Cash transfers of approximately USD 31 were provided every 3 months for 18 months to AGYW who attended at least 10 hours of a behaviour change and communication (BCC) curriculum. Girls AGYW who completed the BCC curriculum and received cash were offered to to participate in a small group financial literacy and individual savings and loan programme called WORTH+.	DREAMS project Sauti: 10 hours of BCC curriculum	12 months
6	Wamoyi, Balvanz, Atkins, et al., 2020	AGYW participating in DREAMS CT programme (Sauti project)	Tanzania	Adolescent girls and young women 15–23 years	DREAMS (Sauti Project)	Cash transfer of TZS 70,000 ($ 31) delivered via SIM cards on mobile phones provided by Sauti project every 3 months over an 18-month period, combined with WORTH+ economic empowerment intervention comprised of financial literacy education, individual and group savings and loan, and entrepreneurship skills.	BCC package provided education on HIV and other STI prevention, gender-based violence prevention, family planning, condom use, negotiation skills, self-efficacy/agency skills, and promoted health-seeking behaviors.	18 months
7	Wamoyi, Balvanz, Gichane, et al., 2020	AGYW participating in DREAMS CT programme (Sauti project)	Tanzania	Adolescent girls and young women 15–23 years	DREAMS (Sauti Project)	Cash transfer of TZS 70,000 ($ 31) delivered via SIM cards on mobile phones provided by Sauti project every 3 months over an 18-month period, combined with WORTH+ economic empowerment intervention comprised of financial literacy education, individual and group savings and loan, and entrepreneurship skills.	BCC package provided education on HIV and other STI prevention, gender-based violence prevention, family planning, condom use, negotiation skills, self-efficacy/agency skills, and promoted health-seeking behaviors.	18 months
**Girls Empowerment Programme (GEP)**								
**#**	**Author, year**	**Target Population**	**Location**	**Population: Age & Sex**	**Intervention Name**	**Intervention: Economic Component**	**Intervention: Life skills, Health, Behavioural, or Other Component**	**Duration of Intervention**
8	Berry, Kuriansky, Little, et al., 2013	Adolescent girls and young women 17–22 years who showed potential in becoming community leaders	Lesotho	Adolescent girls and young women 17–22 years who showed potential in becoming community leaders	Girls Empowerment Programme (GEP) Camp	Half day training on income generating activities based on ILO programme, including topics on: generating your business idea; starting the business; and improving the business. Information on financial support was also provided. Half (*n* = 19) of the girls attended a subsequent 2-week workshop in income-generating activities.	Outward Bound Camp; psychosocial and life skills for girls’ empowerment including information on HIV/AIDS prevention.	1 week long camp; 1/2 day training module for income-generating activities
**Research Initiative to Support the Empowerment of Girls (RISE)**								
**#**	**Author, year**	**Target Population**	**Location**	**Population: Age & Sex**	**Intervention Name**	**Intervention: Economic Component**	**Intervention: Life skills, Health, Behavioural, or Other Component**	**Duration of Intervention**
9	Milimo, Zulu, Svanemyr, et al., 2021	AGYW in school enrolled in RISE intervention	Zambia	Adolescent girls and young women 14–17 years	Research Initiative to Support the Empowerment of Girls (RISE) trial	School fees (grade 8–9), writing materials, and $3 monthly grant for AGYW & $35 monthly grant for AGYW caregivers	Youth club meetings every 2 weeks for AGYW (provided comprehensive sexual and reproductive health education to adolescent males and females in and out of school). The; community dialogue meetings every 2 months for caregivers (focused on the benefits of education for adolescent females and the postponement of early pregnancy and marriage).	24 months
**Women First and Go Girls!**								
**#**	**Author, year**	**Target Population**	**Location**	**Population: Age & Sex**	**Intervention Name**	**Intervention: Economic Component**	**Intervention: Life skills, Health, Behavioural, or Other Component**	**Duration of Intervention**
10	Burke, Packer, González-Calvo, et al., 2019	Adolescent girls 13–19 years	Mozambique	Adolescent girls and young women 13–19 years in rural Mozambique	Women First and Go Girls!	Women First, using Go Girls! curriculum: trains women to sell products door-to-door in their communities. Included business education followed by the sale of items included in a business “kit”. Participants were expected to re-pay the program for the kits with a portion of their sales to receive the next kit, with the remainder of their revenue considered profit to be spent or saved. Also included accumulated savings and credit associations to encourage saving.	Used the full Go Girls! curriculum and a locally-tailored gender-based violence (GBV) curriculum to encourage social empowerment and reduce adolescent girl participants’ vulnerability to HIV. The intervention also had the goal of encouraging girls to stay in school.	6 years (but start dates and implementation methods varied across communities due to logistic constraints)
11	Lenzi, Packer, Ridgeway, et al., 2019	Girls aged 13–17 (particularly orphans/vulnerable children)	Mozambique	Adolescent girls and young women 13–17 years	Women First and Go Girls!	Trained girls to sell products such as homemade cakes, cooking oil, and soap door-to-door in their communities	Go Girl!, an intervention of 15 facilitator-led sessions covered topics such as harmful gender norms for boys and girls, how to communicate with adults and partners, puberty and pregnancy prevention, HIV prevention, staying in and returning to school, preventing unwanted advances, planning goals, and assessing values, money and gifts	Not specified
**Unnamed Intervention**								
**#**	**Author, year**	**Target Population**	**Location**	**Population: Age & Sex**	**Intervention Name**	**Intervention: Economic Component**	**Intervention: Life skills, Health, Behavioural, or Other Component**	**Duration of Intervention**
12	Banda, Svanemyr, Sandøy, et al., 2019	Youth in five rural schools and surrounding communities in Monze and Pemba districts of Zambia.	Zambia (Southern Province)	Purposive sampling of youth participants in communities receiving economic, community, and youth club components of RISE trial) & community members	None specified	Monthly cash transfers and school fees for participating girls	Community meetings and youth clubs (for girls) to provide SRH education, life skills (HIV, family planning, pregnancy, menstruation, negotiation, conflict resolution, management skills, legal knowledge on women’s issues), recreational activities, with female mentors	Intervention was 2 years and qualitative data were collected 14 months into the trial.
13	Mason, Zulaika, van Eijk, et al., 2022	AGYW attending secondary day school	Kenya	Adolescent girls and young women (age range not specified)	None specified	Cash transfer (KES1500 per school term), conditional upon 80% or greater attendance of school	Menstrual cup use + puberty and hygiene education (e.g., SRH knowledge and menstrual hygiene)	5 years
14	Sitienei & Pillay, 2019	Orphans & vulnerable children (OVC) selected from a community-based organization (CBO)	South Africa	Orphaned male and female adolescents 10–18 years	None specified	School fees and assisting the caregivers with obtaining grants from the government.	Psychosocial support/mentoring, healthcare, life skills training, and transport.	2 years
15	Skovdal, 2010	Caregiving children for parents with HIV/AIDS	Kenya	Male and female adolescents 12–17 years	None specified	Children are given cash or provided with items (e.g., chickens) for their activities	Children split into clubs & trained to develop action plan, project management, & book keeping skills	21 months

### Analysis

Aggregate qualitative and quantitative data were used in analysis. Following the PRISMA guidelines, we analyzed recurring patterns of impacts on outcomes across studies. We organized findings by intervention type and according to 12 categories of outcomes (Table 4). We rated the strength of causal identification of each quantitative study as low (observational, pre/post), medium (uses quasi-experimental methods to construct a counterfactual; RCT but low number of clusters), and high (RCTs with adequate number of units of randomization). Qualitative studies were not rated in terms of quality of causal identification, as this is not in line with the qualitative research approach.

In the summary charts and results below, counts refer to quantitative studies, and qualitative studies are described separately. One study (Berry, et al. [36]) was framed as a quantitative study, but we analyzed it together with qualitative studies because the sample size was small (*N* = 40), and the authors did not conduct any statistical tests. In quantitative studies that included a control or comparison group, we counted protective effects only as those that were statistically significantly different between treatment and control/comparison groups and not significant changes over time within the treatment group that may have been reported (e.g., Jennings et al. [37]). Otherwise, if studies only examined pre/post comparisons and did not have a control group, we counted significant differences over time within the treatment group (e.g., Naledi and colleagues [38]). In our counts, if a study examined two sexual risk behaviours, for example, and on one a protective effect was found and on the other a null effect was found, we counted this as the study having a protective effect on sexual risk behaviours in our overall count. However, if a study examined only one outcome in a category and found positive effects in one sub-group and null effects in another sub-group, we counted this as a mixed effect (and vice versa for adverse effects). For the purposes of this paper, separate implementation sites, age groups, and sexes were considered sub-groups; various treatment arms were not. If an effect was positive in one sub-group and not significant in another, it was considered mixed (likewise for negative effects). In contrast, if an effect was positive in one intervention treatment arm, and not significant in another, it was considered a positive effect for the intervention (likewise for negative effects).

## Results

### Search process

**Fig. 2:**
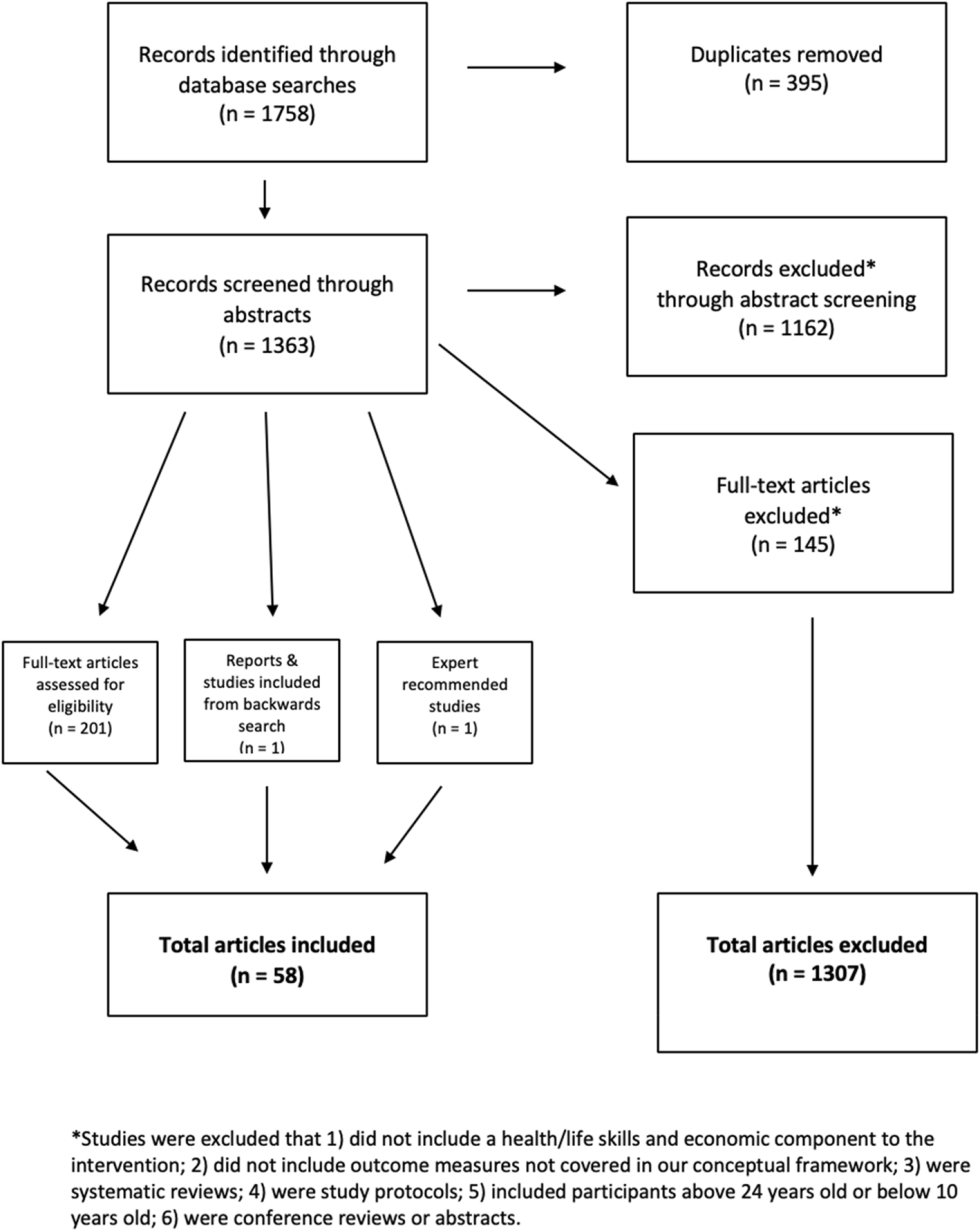
PRISMA flowchart for inclusion and exclusion criteria

A total of 1758 records were identified through the database searches (see Fig. 2). Of these studies, 395 duplicates were removed, and the remaining 1363 abstracts were screened. During the screening process, 1162 studies were excluded based on eligibility criteria. In the full-text review, 201 articles were then screened for eligibility, and 145 were excluded. Expert guidance recommended one additional paper and a backwards search of the included literature added one additional article. A total of 58 peer-reviewed studies were included in the final review, with several studies (those examining DREAMS specifically) including results in multiple countries. A majority of the studies reviewed came from Eastern and Southern Africa. Sixteen studies were conducted in Uganda; 12 each in Kenya and Tanzania; 9 in South Africa; five in Zambia; three each in Lesotho, Zimbabwe, Malawi, and Mozambique; and one each in Ethiopia and Liberia. Of the 58 studies, 43 were quantitative: 27 (46.5%) were randomized or cluster randomized control trials, seven (12%) were quasi-experimental, nine (15.5%) were observational; and 15 (26%) were qualitative. Thirty-eight studies included only adolescent girls and young women, 17 included both adolescent boys and girls, and three involved both AGYW as well as members of their community (e.g., caretakers, community leaders).

### Intervention components

The 58 studies included in this review examined various outcomes from 26 interventions: Adolescent Girls Empowerment Programme (AGEP) in Zambia; Adolescent Girls Initiative – Kenya (AGI-K), also in Kenya; Empowerment and Livelihood for Adolescents (ELA) in both Uganda and Tanzania; Girls Empowerment in Lesotho; DREAMS, as implemented in Kenya, Lesotho, Malawi, South Africa, Zambia, and Zimbabwe; the Suubi, Suubi-Maka, and Suubi4Her studies in Uganda; Women First and Go Girls! in Mozambique; Ujana Salama: Cash Plus Model for Safe Transitions to a Healthy and Productive Adulthood in Tanzania; SHAZ! (Shaping the Health of Adolescents in Zimbabwe) in Zimbabwe; Biruh Tesfa (Bright Future) in Ethiopia; the Sauti Project in Tanzania; Bridges to the Future and BridgesPLUS in Uganda; Research Initiative to Support the Empowerment of Girls (RISE) trial in Zambia; Women of Worth in South Africa; Girl Empower in Liberia; and seven unnamed interventions. As such, each study does not represent a unique intervention; rather, the studies are grouped by outcomes and protective factors as relevant to this review’s conceptual framework. In the results that follow, we indicate how many studies found protective associations/effects in each outcome category, and then in parenthesis we list the unique number of interventions with protective effects. When listing percentages, we refer to the percentage of studies which found protective effects on at least one indicator in the outcome category, among the total number of studies which examined that outcome category.

The health/life skills and economic components were not homogenous across studies. Health information included sexual and reproductive health (SRH) knowledge incorporating menstruation, fertility, family planning, and condom use, STI/HIV knowledge and testing, mental health, and/or general knowledge of health resources. Life skills included gender attitudes, GBV-awareness education, conflict management skills, decision-making skills, and/or empowerment. Mentoring was either from a peer or other community member, and vocational training incorporated business planning, skill building workshops, entrepreneurial training, and/or income-generating activities. Fiscal literacy included wealth and savings education, economic development training, and/or financial management skills.

Combinations of intervention components were also not homogenous across studies. As such, we separated out the most frequent combinations, with the understanding that many studies had multiple overlapping combinations (e.g., a study included in the cash plus health information combination may also have a vocational training plus health information component): 23 studies had cash plus health information and 22 had cash plus some life skills component; 20 had cash plus mentoring and 20 studies included vocational training plus life skills; 18 included vocational training plus health information; 14 studies incorporated cash plus a savings account, fiscal literacy, and health information; 6 studies included vocational training plus a mentorship component, while 4 studies incorporated vocational training with microcredit, health, and life skills training; one study had fiscal literacy plus health information plus life skills; and one had a health voucher plus a savings account, fiscal literacy, and life skills. These categories are not mutually exclusive, and the totals sum to more than the 58 studies and 26 interventions reviewed.

Lastly, 13 studies evaluated DREAMS programming, where a majority (*n* = 11) did not specify what intervention components they were evaluating. DREAMS interventions generally combine core packages to empower girls and young women (condoms, PrEP, violence prevention and post-violence care, HIV testing and counselling, increasing contraceptive method mix, social asset building), reduce the risk of sexual partners (provision of ART to male partners), strengthen families (parenting and caregiver programmes, cash transfers, educational subsidies, socio-economic approaches), and mobilize communities for change (school-based HIV prevention and community mobilization/norms change) [39]. However, intervention components and implementation varied greatly – many of the sites that implemented DREAMS used only a few components of the overall DREAMS programming, but the studies that examined or evaluated DREAMS often did not specify which components were implemented in their study sites. This made it difficult to understand what most of the DREAMS studies we reviewed were actually evaluating.

Table 1 describes the programme components, target population, and location for each of the studies included in our review, both quantitative and qualitative. Tables 2 and 3 summarize the findings, based on the outcomes and mediators of interest, for quantitative and qualitative studies, respectively. Detailed findings, including sample sizes and effect estimates, from all included studies can be found in Appendix 4 for quantitative studies and Appendix 5 for qualitative studies.

**Table 2:**
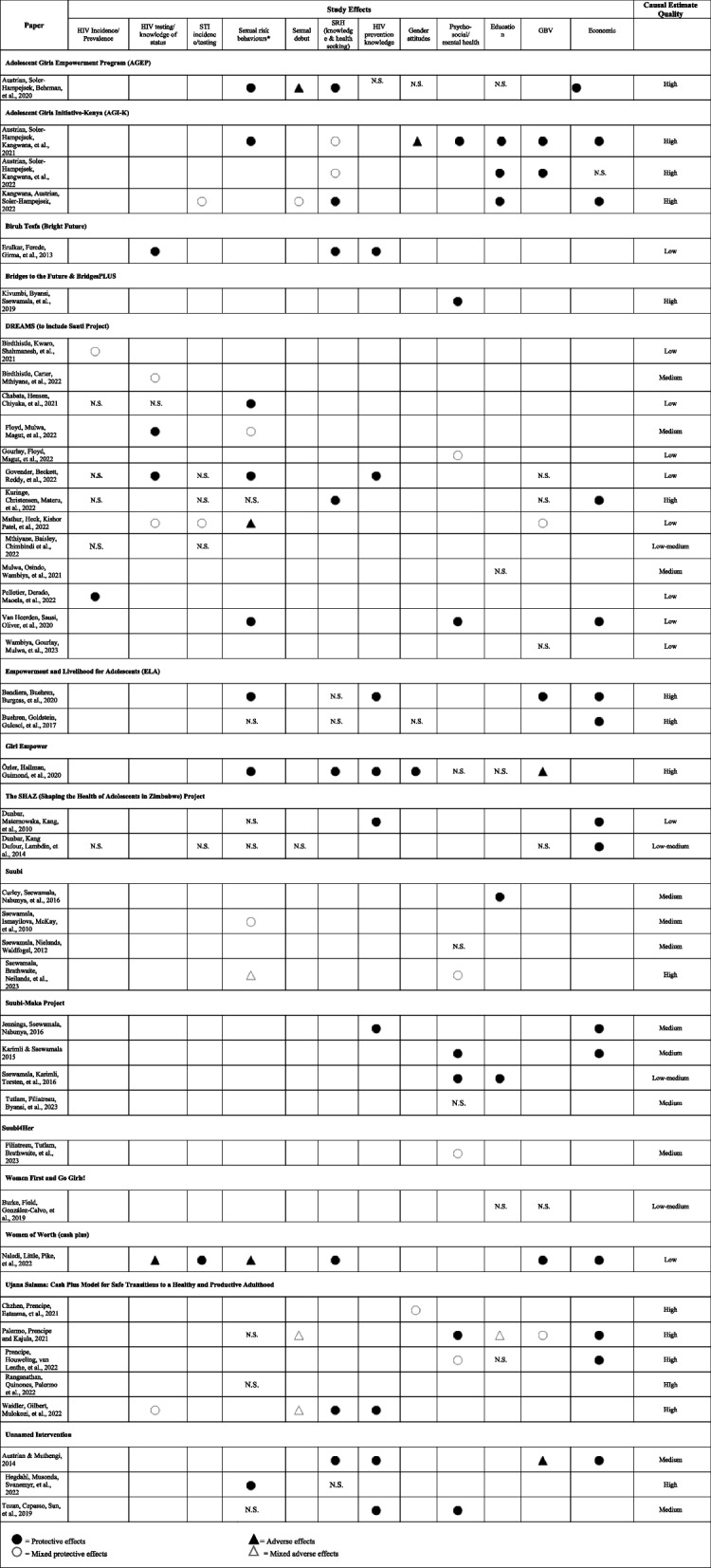
Quantitative study outcomes

**Table 3:**
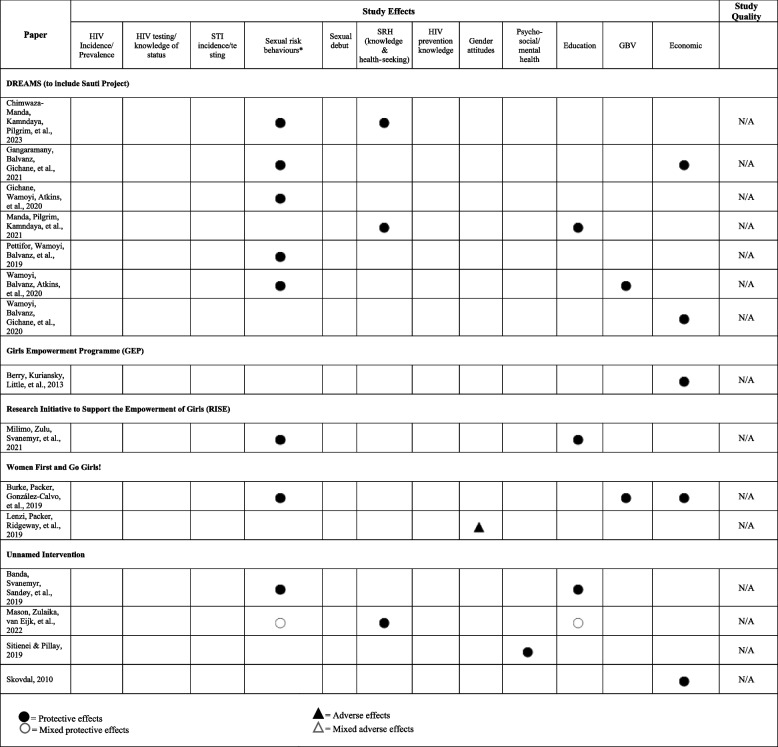
Qualitative study outcomes

### Quality of causal identification strategies

Among the 43 quantitative studies, in terms of the study design’s ability to estimate causal impacts, 16 studies were rated as high, 12 as medium, 4 as low-medium, and 11 were rated as low quality (see Appendix 4). Study quality was determined by the rigor of the study design’s ability to causally identify impacts (e.g., RCTs with sufficient cluster size were rated as high, RCTs with low numbers of clusters and quasi-experimental designs were rated as medium, and observational studies without a causal identification strategy were rated as low).

### High-level findings

We grouped outcomes into 12 categories across the 58 studies as follows: HIV incidence/prevalence; HIV testing or knowledge of HIV status; HIV prevention knowledge; STI incidence, prevalence, and/or testing (separate from HIV); sexual risk behaviour; early sexual debut; sexual and reproductive health; gender attitudes; gender-based violence; psycho-social well-being and mental health; education; and economic outcomes (see Table 4 for effectiveness of interventions by outcome category). Forty-three studies examined these outcomes quantitatively, and 15 qualitatively.

**Table 4:** Effectiveness of bundled interventions

Bundle	Studies that Map to Bundle^a^	HIV Incidence	HIV Testing	STI Incidence/Testing	Sexual Risk Behaviours	Sexual Debut	SRH	HIV prevention knowledge	Gender Attitudes	Psycho social well-being	Education	GBV	Economic
**Cash + health information (23)**	**Study #:** 1, 2, 3, 4, 9, 22, 26, 27, 28, 29, 30, 31, 32, 33, 35, 36, 37, 38, 39 40, 41, 42, 43	0 of 1 full (0%) 0 of 1 mixed (0%) **Total: 0%**	0 of 3 full (0%) 1 of 3 mixed (0%) **Total: 0%**	1 of 2 full (50%) 1 of 2 mixed (50%) **Total: 100%**	5 of 11 full (45%) 1 of 11 mixed (1%) **Total: 46%**	0 of 4 full (0%) 1 of 4 mixed (25%) **Total: 25%**	6 of 9 full (66%) 2 of 9 mixed (22%) **Total: 88%**	5 of 6 full (83%) 0 of 6 mixed (0%) **Total: 83%**	1 of 4 full (25%) 1 of 4 mixed (25%) **Total: 50%**	5 of 11 full (45%) 3 of 11 mixed (27%) **Total: 72%**	4 of 8 full (50%) 0 of 8 mixed (0%) **Total: 50%**	3 of 6 full (50%) 1 of 6 mixed (17%) **Total: 67%**	9 of 10 full (90%) 0 of 10 mixed (0%) **Total: 90%**
**Cash + life skills (22)**	**Study #:** 1, 2, 3, 4, 9, 22, 25, 26, 27, 28, 29, 30, 31, 32, 33, 35, 36, 37, 38, 39, 40 41	0 of 1 full (0%) 0 of 1 mixed (0%) **Total: 0%**	0 of 3 full (0%) 1 of 3 mixed (33%) **Total: 33%**	1 of 2 full (50%) 1 of 2 mixed (50%) **Total: 100%**	4 of 9 full (44%) 1 of 9 mixed (11%) **Total: 55%**	0 of 4 full (0%) 1 of 4 mixed (25%) **Total: 25%**	6 of 8 full (75%) 2 of 8 mixed (25%) **Total: 100%**	4 of 5 full (80%) 0 of 5 mixed (0%) **Total: 80%**	1 of 4 full (25%) 1 of 4 protective (25%) **Total: 50%**	4 of 10 full (40%) 3 of 10 mixed (30%) **Total: 70%**	5 of 9 full (55%) 0 of 9 mixed (0%) **Total: 55%**	3 of 6 full (50%) 1 of 6 mixed (17%) **Total: 67%**	9 of 10 full (90%) 0 of 10 mixed (0%) **Total: 90%**
**Cash + mentoring (20)**	**Study #:** 1, 2, 3, 4, 22, 25, 26, 27, 28, 29, 30, 31, 32, 36, 37, 38, 39, 40, 41, 43	N/A	0 of 1 full (0%) 1 of 1 mixed (100%) **Total:** **100%**	0 of 1 full (0%) 1 of 1 mixed (100%) **Total: 100%**	3 of 8 full (37%) 1 of 8 mixed (12%) **Total: 49%**	0 of 4 full (0%) 1 of 4 mixed (25%) **Total: 25%**	5 of 7 full (71%) 2 of 7 mixed (28%) **Total: 99%**	5 of 6 full (83%) 0 of 6 mixed (0%) **Total: 83%**	1 of 4 full (25%) 1 of 4 mixed (25%) **Total: 50%**	5 of 10 full (50%) 2 of 10 mixed (20%) **Total: 70%**	5 of 9 full (55%) 0 of 9 mixed (0%) **Total: 55%**	2 of 5 full (40%) 1 of 5 mixed (20%) **Total: 60%**	8 of 9 full (88%) 0 of 9 mixed (0%) **Total: 88%**
**Vocational training + health information (18)**	**Study #:** 9, 18, 20, 21, 23, 24, 26, 27, 28, 29, 30, 31, 34, 36, 37, 38, 39, 40	0 of 2 full (0%) 0 of 2 mixed (0%) **Total: 0%**	0 of 2 full (0%) 1 of 2 mixed (50%) **Total: 50%**	0 of 1 full (0%) 0 of 1 mixed (0%) **Total: 0%**	3 of 10 full (30%) 1 of 10 mixed (0%) **Total: 30%**	0 of 3 full (0%) 0 of 3 mixed (0%) **Total: 0%**	1 of 3 full (33%) 0 of 3 mixed (0%) **Total: 33%**	4 of 4 full (100%) 0 of 4 mixed (0%) **Total: 100%**	0 of 2 full (0%) 1 of 2 mixed (50%) **Total: 50%**	4 of 7 full (57%) 2 of 7 mixed (28%) **Total: 85%**	1 of 4 full (25%) 0 of 4 mixed (0%) **Total: 25%**	1 of 4 full (25%) 1 of 4 mixed (25%) **Total: 50%**	9 of 9 full (100%) 0 of 9 mixed (0%) **Total: 100%**
**Vocational training + life skills (20)**	**Study #:** 9, 18, 20, 21, 23, 24, 26, 27, 28, 29, 30, 31, 32, 33, 34, 36, 37, 38, 39, 40	0 of 2 full (0%) 0 of 2 mixed (0%) **Total: 0%**	0 of 2 full (0%) 1 of 2 mixed (50%) **Total: 50%**	0 of 1 full (0%) 0 of 1 mixed (0%) **Total: 0%**	3 of 10 full (30%) 1 of 10 mixed (1%) **Total: 40%**	0 of 3 full (0%) 0 of 3 mixed (0%) **Total: 0%**	1 of 3 full (33%) 0 of 3 mixed (0%) **Total: 33%**	4 of 4 full (100%) 0 of 4 mixed (0%) **Total: 100%**	0 of 2 full (0%) 1 of 2 mixed (50%) **Total: 50%**	4 of 9 full (44%) 3 of 9 mixed (33%) **Total: 77%**	1 of 4 full (25%) 0 of 4 mixed (0%) **Total: 25%**	1 of 4 full (25%) 1 of 4 mixed (25%) **Total: 50%**	9 of 9 full (100%) 0 of 9 mixed (0%) **Total: 100%**
**Savings account + cash + fiscal literacy + health information (14)**	**Study #:** 1, 6, 22, 25, 26, 27, 28, 29, 30, 31, 32, 33, 41, 43	N/A	N/A	N/A	2 of 5 full (40%) 1 of 5 mixed (20%) **Total: 60%**	0 of 1 full (0%) 0 of 1 mixed (0%) **Total: 0%**	3 of 3 full (100%) 0 of 3 mixed (0%) **Total: 100%**	4 of 5 full (80%) 0 of 5 mixed (0%) **Total: 80%**	1 of 2 full (50%) 0 of 2 mixed (0%) **Total: 50%**	4 of 9 full (44%) 2 of 9 mixed (22%) **Total: 63%**	2 of 4 full (50%) 0 of 4 mixed (0%) **Total: 50%**	0 of 2 full (0%) 0 of 2 mixed (0%) **Total: 0%**	4 of 4 full (100%) 0 of 4 mixed (0%) **Total: 100%**
**Vocational training + microcredit + health + life skills (4)**	**Study #:** 18, 20, 23, 24	0 of 1 full (0%) 0 of 1 mixed (0%) **Total: 0%**	N/A	0 of 1 full (0%) 0 of 1 mixed (0%) **Total: 0%**	2 of 4 full (50%) 0 of 4 mixed (50%) **Total: 50%**	0 of 1 full (0%) 0 of 1 mixed (0%) **Total: 0%**	0 of 1 full (0%) 0 of 1 mixed (0%) **Total: 0%**	2 of 2 full (100%) 0 of 2 mixed (0%) **Total: 100%**	N/A	1 of 1 full (100%) 0 of 1 mixed (0%) **Total: 100%**	N/A	1 of 2 full (50%) 0 of 2 mixed (0%) **Total: 50%**	4 of 4 full (100%) 0 of 4 mixed (0%) **Total: 100%**
**Vocational training + mentoring (6)**	**Study #:** 36, 37, 38, 39, 40, 43	N/A	0 of 1 full (0%) 1 of 1 mixed (100%) **Total: 100%**	N/A	0 of 3 full (0%) 0 of 3 mixed (0%) **Total: 0%**	0 of 2 full (0%) 0 of 2 mixed (0%) **Total: 0%**	1 of 1 full (100%) 0 of 1 mixed (0%) **Total: 100%**	2 of 2 full (100%) 0 of 2 mixed (0%) **100%**	0 of 1 full (0%) 1 of 1 mixed (100%) **Total: 100%**	2 of 3 full (66%) 1 of 3 mixed (33%) **Total: 99%**	0 of 2 full (0%) 0 of 2 mixed (0%) **Total:** **0%**	0 of 1 full (0%) 1 of 1 mixed (100%) **Total: 100%**	2 of 2 full (100%) 0 of 2 mixed (0%) **Total: 100%**
**Voucher + savings account + fiscal literacy + life skills (1)**	**Study #:** 1	N/A	N/A	N/A	1 of 1 full (100%) 0 of 1 mixed (0%) **Total: 100%**	0 of 1 full (0%) 0 of 1 mixed (0%) **Total: 0%**	1 of 1 full (100%) 0 of 1 mixed (0%) **Total: 100%**	0 of 1 full (0%) 0 of 1 mixed (0%) **Total: 0%**	0 of 1 full (0%) 0 of 1 mixed (0%) **Total: 0%**	N/A	0 of 1 full (0%) 0 of 1 mixed (0%) **Total: 0%**	N/A	1 of 1 full (100%) 0 of 1 mixed (0%) **Total: 100%**
**Fiscal literacy + health information + life skills (1)**	**Study #:** 5	N/A	1 of 1 full (100%) 0 of 1 mixed (0%) **Total: 100%**	N/A	N/A	N/A	1 of 1 full (100%) 0 of 1 mixed (0%) **Total: 100%**	1 of 1 full (100%) 0 of 1 mixed (0%) **Total: 100%**	N/A	N/A	N/A	N/A	N/A
**DREAMS:** **unspecified (11)**	**Study #:** 7, 8, 10, 11, 12, 13, 14, 15, 16, 17, 19	1 of 5 full (20%) 1 of 5 mixed (20%) **Total: 40%**	2 of 4 full (50%) 2 of 4 mixed (50%) **Total: 100%**	0 of 4 full (0%) 1 of 4 mixed (25%) **Total:** **25%**	1 of 4 full (25%) 1 of 4 mixed (25%) **Total:** **50%**	N/A	1 of 1 full (100%) 0 of 1 mixed (0%) **Total:** **100%**	1 of 1 full (100%) 0 of 1 mixed (0%) **Total:** **100%**	N/A	0 of 1 full (0%) 1 of 1 mixed (100%) **Total: 100%**	0 of 1 full (0%) 0 of 1 mixed (0%) **Total: 0%**	0 of 4 full (0%) 1 of 4 mixed (25%) **Total: 25%**	1 of 1 full (100%) 0 of 1 mixed (0%) **Total: 100%**

^a^Study numbers coincide with numerical ordering in Table 1. Qualitative studies not included in effectiveness counts.

### HIV outcomes (testing, knowledge of status, incidence)

Two out of seven quantitative studies examining impacts on HIV incidence/prevalence found a protective association. (Protective effects were found in one of the three interventions that included HIV testing/prevalence). Two of these studies found a protection association with the time trend over the period examined in Lesotho and South Africa (but not Kenya) [40, 41], while two studies in Zimbabwe, one study in Kenya and South Africa, one in Tanzania, and another in South Africa found no protective effects [42–46]. Four of the studies examining HIV incidence had low quality with respect to study design and identification of causal impacts [40–42, 44], two were rated as low-medium quality [43, 46], and one was high quality [45].

Four out of eight quantitative studies examining outcomes related to HIV testing and/or knowledge of HIV status found protective effects, while one found adverse effects. (Protective effects were found in three of the four interventions that included HIV testing and/or related knowledge.) In Ethiopia, Erulkar et al. [47] found that girls in treatment areas were more likely to want voluntary HIV testing and counselling than those in the comparison group. In Tanzania, Waidler and colleagues [48] found a significant increase in HIV testing. Floyd and colleagues [49] found positive impact on knowledge of HIV status for all DREAMS participants in both Kenya and South Africa. In another DREAMS-related study, Govender and colleagues [44] found that participants were more likely to have been HIV tested. In adverse effects, in South Africa, Naledi and colleagues [38] found that, compared to baseline, the intervention group had significantly lower odds of self-reporting HIV testing in the last 6 months, as well as reduced reporting of perceived HIV risk. At a second, post-intervention follow-up, impacts on testing and risk perceptions were no longer significant [38].

Two studies found mixed protective effects. Birdthistle and colleagues [50] found that knowledge of HIV status was higher among DREAMS beneficiaries compared to non-beneficiaries in Kenya, while in South Africa associations were significant in a younger age group (13–17 years) but not in an older group (18–22 years). Mathur and colleagues [51] examined HIV risk-related behaviours among DREAMS participants in Kenya, Malawi, and Zambia and found mixed protective effects. In Zambia and Malawi, HIV testing increased among all participants, and was significant for both adolescent girls (15–19 years) and young women (20–24 years). In Kenya, HIV testing also increased among all participants, and was significant among adolescent girl participants (15–19 years), but not those 20–24 years old. One study in Zimbabwe found no association with knowledge of HIV status [42].

### HIV prevention knowledge outcomes

Nine out of 10 studies (nine out of nine interventions) quantitatively examining HIV prevention knowledge found protective effects. In Uganda, Austrian and Muthengi [52] found that AGYW in their SavingsPLUS arm were more likely to understand HIV transmission mechanisms and HIV prevention methods. Bandiera and colleagues [53] found that the HIV knowledge index increased among intervention participants (although this effect was not sustained at endline). Erulkar and colleagues [47] in Ethiopia found that girls in treatment areas had higher HIV knowledge than those in comparison areas. In Zimbabwe, Dunbar and colleagues [54] found that intervention participants had significantly increased HIV prevention knowledge. In Uganda, Jennings et al. [37] found that adolescents in the intervention increased HIV prevention attitudinal scores and higher odds of a maximum HIV-prevention score. Özler et al. [55] found that participants in both study arms of Girl Empower (GE and GE+) had increased HIV knowledge. Tozan and colleagues [56] found in Uganda that participants in both treatment arms (Bridges and Bridges PLUS) had significantly increased HIV knowledge over the control arm, but there were no impacts on HIV prevention attitudes. In Tanzania, Waidler et al. [48] found the intervention increased HIV-related knowledge. Govender and colleagues found that among participants in DREAMS-like interventions, exposure to an increasing number of interventions was associated with higher HIV prevention knowledge [44]. In Zambia, Austrian and colleagues [57] did not find any impacts on participants’ knowledge of HIV.

### STI incidence, symptoms, and testing outcomes

One out of seven quantitative studies (one out of five interventions) examining STI testing, symptoms and incidence outcomes found protective effects. Naledi et al. [38] found that the intervention increased STI testing in South Africa immediately following the intervention, but impacts were no longer significant at the post-intervention follow-up. Two studies found mixed protective effects. In Kenya, Kangwana and colleagues [58] found significant reductions in HSV-2 prevalence and incidence, but only among a younger sub-sample (13–14 years at baseline) and not in the full sample. Mathur et al. [51] found that STI symptoms were reduced in DREAMS participants 20–24 years old (but not among those 15–19 years) in Malawi; in Kenya, they found no association between DREAMS participation and STI symptoms. In another study, Govender and colleagues [44] found no association between the DREAMS-like interventions and STI prevalence, and in yet another DREAMS study, Mthiyane et al. [46] found no association between the intervention and HSV-2 incidence. In Zimbabwe, Dunbar and colleagues [43] found no impacts of the intervention on HSV-2, nor did Kuringe and colleagues [45] find association between the intervention and HSV-2 incidence in Tanzania.

### Sexual risk behaviour outcomes

Eight out of 20 studies (seven out of 11 interventions) that quantitatively examined outcomes related to sexual risk behaviours found protective effects on at least some outcomes. In Zambia, the intervention evaluated by Austrian et al. [57] reduced engagement in transactional sex. In another study in Kenya, Austrian and colleagues [59] found that the intervention increased condom use self-efficacy scores. Bandiera and colleagues [53] found that the intervention increased the probability of always using a condom. Chabata and colleagues [42] found that residence in a DREAMS area was associated with increased ability to negotiate condom use and decreased odds of condom-less sex with a regular partner. In Liberia, Özler et al. [55] found that participants in both intervention arms of Girl Empower had fewer sexual partners. In Lesotho, Van Heerdan et al. [60] found that DREAMS beneficiaries reported lower levels of sexual risk-taking than non-DREAMS participating peers. In Kenya and South Africa, Govender and colleagues [44] found that exposure to two or more DREAMS-like interventions increased the likelihood of condom use (although they found no association between the interventions and age-disparate sex). In Zambia, Hegdahl and colleagues [61] found that both intervention arms reduced the risk of adolescent girls having been sexually active in the past month, and the combined cash plus community support arm also reduced the risk of unprotected sex among participants.

Two studies found mixed protective effects. In Uganda, Ssewamala and colleagues [62] found that the intervention was protective against sexual risk-taking attitudes among boys, but not among girls. Floyd and colleagues [49] found that, in Kenya, DREAMS reduced condomless sex among 18–22-year-old girls and reduced the odds of having more than two lifetime partners among all DREAMS participants in Kenya, but there were no effects on these outcomes in South Africa. However, Floyd and colleagues [49] found no impact of DREAMS on transactional sex in Kenya and South Africa.

Three studies found adverse programme effects. In Kenya, Mathur and colleagues [51] found DREAMS participants were less likely to use condoms consistently and were more likely to engage in transactional sex (authors found no association with DREAMS and these outcomes in Malawi). In South Africa, Naledi and colleagues [38] also found that participants were less likely to use a condom at last sex. In another study in Uganda, Ssewamala and colleagues [63] found mixed adverse effects: while there appeared to be no effect on sexual risk or attitudes toward condom use at the 24-month follow-up overall, the combined intervention arm reported reduced favourable attitudes toward condoms at 12 months, and the savings-only arm increased sexual risk behaviour at 24 months.

Seven studies found no impacts. Buehren et al. [64] found no programmatic impacts on knowledge of safe sexual behaviour among participants in Tanzania. In Zimbabwe, Dunbar and colleagues [54] also found no impacts in regards to condom use, sexual activity, transactional sex, life preferences, or power in sexual relationship among participants over time. In a separate study in Zimbabwe, Dunbar and colleagues [43] found no intervention effects on condom use, transactional sex, or other sexual activity. In Tanzania, Palermo et al. [65] found no effects of Ujana Salama on age-disparate partnerships. In another study of the same intervention in Tanzania, Ranganathan and colleagues [66] found no impacts on engagement in transactional sex. In Uganda, Tozan and colleagues [56] found no programmatic impacts on sexual risk taking. Last, in Tanzania, Kuringe and colleagues [45] did not find protective effects for the DREAMS Sauti Project on any of the following behaviours: transactional sex, intergenerational sex, condom use, and number of sexual partners.

Eight studies (among five unique interventions) evaluated sexual risk behaviour outcomes qualitatively. Both Banda et al. [67] and Gangaramany and colleagues [68] found that economic support helped to decrease adolescent girls’ reliance on and engagement in transactional sex. Mason and colleagues [69] also found that girls who received both cash and cash plus menstrual education reported feeling empowered to refuse male sexual advances as well as a reduced need to engage in transactional sex due to the extra money. In Zambia, Burke et al. [70] found that the intervention supported less engagement in both transactional sex and age-disparate (intergenerational) partnerships, most likely due to the cash that participants received during the intervention period. In Zambia, Milimo et al. [71] found that adolescent girls had a decreased desire to pursue relationships with boys in exchange for money or gifts. Gichane et al. [72], Pettifor et al. [73], and Wamoyi and colleagues [74] all explored sexual risk behaviour within the Sauti Project Worth+ intervention. Gichane and colleagues [72] found similar results among their study participants in Tanzania, with participants less likely to engage in transactional sex. Pettifor and colleagues [73] found two primary mechanisms to reduce dependence on male sex partners through transactional sex in Tanzania: the first was that the cash provided for more basic needs, such as food or toiletries. The second was that the financial education component of the intervention appeared to empower participants to reject sexual partners. In Malawi, Chimwaza-Manda et al. [75] found that DREAMS participants who were also in Girls Only Clubs consulted others on decision-making and information on sexual relationships, used condoms, and quit sexual relationships more than their non-club counterparts Additionally, they corrected sexual misinformation among their peers with information they learned from the club. Last, Wamoyi and colleagues [74] found that cash transfers among participants in Tanzania empowered adolescent girls to reduce their participation in transactional sex, along with other risky sexual behaviour.

### Sexual debut outcomes

None of the five quantitative studies (across four interventions) examining sexual debut found fully protective effects. However, mixed protective effects were found in one of the four studies (in one intervention). Kangwana and colleagues [58] found in Kenya significant reductions in the percentage of AGYW ever having sex only among those intervention participants who were 13–14 years old at baseline. In Tanzania, Waidler and colleagues [48] and Palermo and colleagues [65] (both examining the same programme) found mixed results, where treatment females sexually debuted earlier than controls, but there were no effects among males. In Zambia, Austrian and colleagues [57] found adverse effects, where intervention participants had an increased probability of ever having sex. Lastly, in Zimbabwe, Dunbar et al. [43] found no effects on sexual debut.

### Sexual and reproductive health outcomes

Eight out of 13 quantitative studies (eight out of 10 interventions) found protective effects on SRH outcomes. In Uganda, Austrian and Muthengi [52] found that participants had increased knowledge of contraception. In Zambia, Austrian et al. [57] found the intervention increased SRH knowledge at both Rounds 3 and 5. In Ethiopia, Erulkar et al. [47] found that intervention participants were significantly more likely to know where to obtain voluntary health counselling and testing. In Kenya, two years post-intervention, Kangwana and colleagues [58] found that participants had increased SRH knowledge. Naledi and colleagues [38] found increased contraceptive use among participants following the intervention. Özler et al. [55] found positive effects on SRH knowledge in both treatment arms of the Girl Empower intervention in Liberia. In Tanzania, Waidler and colleagues [48] found the Ujana Salama intervention increased contraceptive knowledge and knowledge of where to seek condoms; however, they also found mixed effects in that the intervention increased health seeking for SRH among males, but not females. Also in Tanzania, Kuringe and colleagues [45] found increased use of biomedical services among DREAMS Sauti participants.

In Kenya, Austrian and colleagues [59] found mixed protective effects. The intervention increased SRH knowledge and knowledge of modern contraceptives in Kibera; however, in Wajir, the intervention reduced contraceptive knowledge.

Four studies found no significant impacts on SRH. In Kenya, Austrian et al. [76] did not find an increase in SRH knowledge nor fertility outcomes in the full sample; however, among those out of school at baseline, the intervention reduced the probability of pregnancy and the fertility summary z-score. Buehren and colleagues [64] did not find any programme impact on knowledge of safe sexual practice or reproductive health for adolescents in Tanzania, and in Uganda, Bandiera and colleagues [53] found no impact on contraceptive use. In Zambia, Hegdahl and colleagues [61] found no impacts on contraceptive use, knowledge of contraceptives, or norms surrounding contraceptive use for either treatment arm.

Two qualitative studies also examined sexual and reproductive health outcomes. In Malawi, Manda and colleagues [77] found that girls’ only clubs (as part of the DREAMS network) were influential for SRH knowledge acquisition among young adolescent girls. In Kenya, Mason et al. [69] found that girls who received menstrual, puberty, and hygiene education reported feelings of empowerment as a result of this knowledge (although only those who also received cash reported behaviour change).

### Gender attitudes outcomes

One out of five studies (across five unique interventions) that included outcomes related to gender attitudes found fully protective effects, and one study found mixed protective effects. Özler and colleagues [55] found an increase in gender equitable attitudes regarding IPV among study participants in both arms of Girl Empower in Liberia. However, neither treatment arm had effects on the physical or sexual violence experiences index. Chzhen and colleagues [78] found mixed protective effects from the Ujana Salama intervention in Tanzania. In the pooled sample of males and females, gender equitable attitudes increased at midline, but the effect was not sustained at endline. In the sex-stratified sample, they found that while gender equitable attitudes increased for males at both midline and endline, there were no statistically significant impacts on gender equitable attitudes for females.

In Kenya, Austrian et al. [59] observed adverse effects, finding reduced gender equitable attitudes among intervention participants in Wajir.

Two studies had no significant findings related to gender attitudes. In Kenya, Austrian and colleagues [57] found no significant programme impacts on gender norms. Buehren et al. [64] found no impacts in ITT models on perceived gender role index in Tanzania.

Lenzi and colleagues [79] found in their qualitative evaluation that participants believed the intervention had reinforced or taught harmful gender stereotypes encouraging girls to be “good” (e.g., deferential and obedient, subservient to their families, and sexually chaste), despite objectives to the contrary.

### Gender-based violence outcomes

Four out of the 13 quantitative studies (three out of 10 interventions) that examined gender-based violence-related outcomes found fully protective effects. Two out of 10 studies (two out of 10 interventions) found mixed protective effects. In Kenya, Austrian et al. [59] found reduced experience of violence among participants in Kibera. Austrian and colleagues [76] also found an increase in the violence prevention outcomes summary index among study participants in Kenya. Bandiera and colleagues [53] found reduced probability of “unwilling sex” among intervention participants at midline (although this effect was not sustained at endline). Naledi and colleagues [38] found that over time, participants reported lower rates of GBV threats and forced sex, but these effects were not sustained at the later follow-up round.

Mathur and colleagues [51] found mixed protective effects for DREAMS beneficiaries who reported reduced violence over time: less sexual IPV was reported among girls 15–19 years and 20–24 years in Kenya and Malawi (but not Zambia); less non-partner sexual violence was reported among women 20–24 years in Kenya and Zambia, and less physical IPV was reported among women 20–24 years in Malawi. In Tanzania, Palermo and colleagues [65] found mixed protective effects in the Ujana Salama intervention. They found reduced sexual violence experiences among the pooled and female samples, but not among males, and reduced physical violence perpetration among males, but not females.

Two studies observed adverse effects. Austrian and Muthengi [52] found that girls in the savings only arm of the intervention had increased odds of being sexually touched or teased by men. In Liberia, Özler and colleagues [55] found that the GE+ arm of the intervention (and the pooled treatment) increased non-consensual touching.

In Mozambique, Burke and colleagues [80] did not observe any programme impacts on participants’ knowledge related to gender-based violence. In Zimbabwe, Dunbar and colleagues [43] also found no effects on experiences of physical or sexual violence, and in Tanzania, Kuringe and colleagues [45] also did not find any impacts on sexual violence. Last, Govender and colleagues [44] found no association between exposure to DREAMS-like interventions and intimate partner violence in either Kenya or South Africa, nor did Wambiya and colleagues find any impact of DREAMS [81] on emotional, physical, or sexual violence in those same countries.

Two studies qualitatively examined GBV-related outcomes from two separate interventions. In Mozambique, Burke et al. [70] found that study participants reported increased knowledge of what types of behaviour constitute gender-based violence, as well as decreased perpetration of sexual or physical violence against women and girls within the community. In Tanzania, Wamoyi and colleagues [74] found that participants reported reduction in experiences of intimate partner violence in connection with an empowerment programme.

### Psycho-social and mental health outcomes

Nine out of 14 studies (five out of eight interventions) that quantitatively examined psycho-social well-being and mental health found protective effects. Austrian and colleagues [59] found that their intervention in Kenya increased the general self-efficacy z-score among participants. In Uganda, Karimli and Ssewamala [82] found that an intervention in Uganda reduced hopelessness and increased self-concept. In Uganda, Kivumbi and colleagues [83] found Bridges to the Future reduced depressive symptoms at Waves 2 and 3 and increased self-concept at Wave 2 (but not Wave 3). They did not find impacts for hopelessness at either wave. In Uganda, Ssewamala and colleagues [84] found lower levels of hopelessness and higher levels of self-concept resulting from the Suubi-Maka Project. In a separate study examining the impact of the Suubi intervention in Uganda, Ssewamala and colleagues [63] found that 24 months post-intervention, the savings-only arm reduced depression and increased self-concept, while the combination savings and family strengthening arm reduced hopelessness and depression and increased self-concept. Also in Uganda, Tozan and colleagues [56] found the Bridges to the Future Plus intervention increased self-concept and self-efficacy, and decreased hopelessness, but the Bridges (no plus component) intervention arm did not have these effects. Van Heerden et al. [60] found that adolescents exposed to DREAMS in Lesotho reported higher levels of self-efficacy as compared with their peers not exposed to DREAMS. In Tanzania, Palermo and colleagues [65] found that the Ujana Salama intervention increased self-esteem. In a separate study of the same intervention in Tanzania, Prencipe and colleagues [85] found the intervention reduced odds of depressive symptoms and increased self-esteem among participants after two years.

Two studies found mixed protective effects. Gourlay and colleagues [86] observed that AGYW reporting having been invited to participate in DREAMS reported higher self-efficacy than AGYW not invited to participate in Kenya (not significant in 2018; in 2019, association was significant among those 18–22 years in Nairobi and 15–17 years in Gem) and South Africa (in 2018 and 2019). In the Suubi4Her intervention in Uganda, Filiatreau and colleagues [87] found reduced hopelessness and increased self-esteem for the treatment arm that combined youth development accounts with a family group intervention, but found no effects for the youth development account only arm.

Özler et al. [55] observed no programmatic impact on the psycho-social index for either intervention arm of Girl Empower in Liberia. While Ssewamala et al. [88] reported a reduction in depression among the treatment group, the difference in slopes over time between treatment and control were not significant, indicating no significant programme impacts. In Uganda, Tutlam et al. [89] found no effects of the Suubi-Maka intervention on outcomes related to prosocial behaviour or emotional and behavioural difficulties.

In South Africa, Sitienei and Pillay [90] qualitatively evaluated participants who received psycho-social support from mentors and peer groups. They found that peer groups especially provided participants with an opportunity to share with and receive support from peers who had had similar experiences.

### Education outcomes

Five out of 11 quantitative studies (three out of eight interventions) examining educational outcomes found protective effects. In Kenya, Austrian et al. [59] found that Adolescent Girls Initiative-Kenya (AGI-K) increased completion of primary school and transition to secondary school (in Kibera), enrolment and grade attainment (in Wajir), and an education outcomes summary z-score (in Kibera and Wajir) after two years. After four years, Austrian and colleagues [76] found that AGI-K intervention increased the education outcomes summary z-score in Wajir. In Uganda, Curley and colleagues [91] found the intervention increased confidence in achieving education plans. In Kenya, Kangwana et al. [58] found that the intervention increased the schooling outcomes index. In Uganda, Ssewamala and colleagues [84] found that the Suubi Maka Project increased the probability of taking the primary leaving exam, confidence in achieving educational plans, and test scores.

One study found mixed adverse effects. In Tanzania, Palermo et al. [65] found that the Ujana Salama intervention reduced school attendance among girls, but not boys. However, when Prencipe et al. [85] examined a combined indicator of school attendance or vocational training, there were no impacts, and forthcoming findings from the larger study (not published at the time of our review and thus not included), indicated that impacts on school attendance were not sustained in later rounds of data collection, and there were no impacts on schooling attainment, suggesting that these adverse effects were temporary in nature and concentrated among a small sub-sample of intervention participants (older females) [92].

Five studies did not observe programmatic impacts for education outcomes. In Zambia, Austrian and colleagues [57] found that the Adolescent Girls Empowerment Programme (AGEP) intervention had no significant impacts on grade completion. In Mozambique, Burke et al. [80] also did not observe any programmatic impacts related to school attendance. Mulwa and colleagues [93] found no association between having been invited to participate in DREAMS and school attendance in Kenya. In Liberia, Özler and colleagues [55] found Girl Empower had no effect on grades attained or the schooling summary index. Last, as mentioned above, in Tanzania, Prencipe et al. [85] found no effects of the Ujana Salama intervention on school attendance and vocational training (combined indicator).

Four studies examined education outcomes qualitatively from four unique interventions. In Zambia, Banda and colleagues [67] observed an increase in school attendance for AGYW who participated in the intervention. In Malawi, Manda and colleagues [77] found that out-of-school female participants reported that when their guardians were economically empowered (e.g., through cash transfers), this facilitated their return to school. Milimo et al. [71] found that economic support increased the motivation to attend and/or remain in school among study participants. Finally, in Kenya, Mason and colleagues [69] found that girls who received menstrual cups reported less school absenteeism due to menstrual concerns, although this did not appear to impact overall school dropout rates.

### Economic outcomes

Fifteen out of 16 studies (10 out of 10 interventions) that quantitatively examined economic outcomes found protective effects. In Uganda, Austrian and Muthengi [52] found that both the savings and savings plus arms of the intervention increased the odds of having a budget and saving money in the last six months. However, the savings-only arm also lowered the odds of participants having knowledge of reasons for saving. In Zambia, Austrian and colleagues [57] found that at round 3, the intervention increased financial literacy and the probability that girls had saved money in the last six months. At round 5, the intervention was found to have increased the probability of saving money. In Kenya, Austrian and colleagues [59] found that AGI-K increased financial literacy, the likelihood of savings, and the wealth outcomes summary z-score in Kibera and increased the probability of having savings and the wealth outcomes summary z-score in Wajir. In Uganda, Bandiera and colleagues [53] found that the ELA intervention increased self-perceived entrepreneurial abilities (significant at endline but not midline), the probability of engaging in any income-generating activities (significant at both rounds), and the probability of being self-employed (significant at endline but not midline). In Tanzania, Buehren and colleagues [64] found no programmatic effects for any key economic indicators, with the exception of the treatment arm including club + microfinance increasing the probability of having savings at a rotating savings and credit schemes (ROSCA). In Zimbabwe, Dunbar and colleagues [54] found that the intervention increased participants having their own income and savings. Qualitatively, however, participants indicated barriers to loan repayment including economic shocks and insufficient financing. In a later study, also in Zimbabwe, Dunbar and colleagues [43] found that Shaz! increased participants’ own income and reduced food insecurity. In Uganda, Jennings et al. [37] found that the intervention increased cash savings and savings attitudes. Kangwana and colleagues [58] found in Kenya that AGI-K increased participants’ wealth creation summary score. In Uganda, Karimli and Ssewamala [82] found that the intervention increased the likelihood of savings and amount saved, at both 12- and 24-months post-intervention. Naledi and colleagues [38] found in South Africa that the intervention increased the probability of being employed. In Tanzania, Palermo and colleagues [65] found that Ujana Salama increased participation in livestock tending. Also in Tanzania, Prencipe et al. [85] found positive impacts on paid work as a result of the Ujana Salama intervention. In Lesotho, Van Heerden et al. [60] found that AGYW who were exposed to DREAMS were more likely to have savings, as well as a plan for how to spend the money they had earned, as compared to peers not exposed to DREAMS. Last, in Tanzania, Kuringe and colleagues [45] found that the DREAMS Sauti intervention increased savings among participants.

Austrian et al. [76] did not find any programmatic impacts on economic outcomes in the full sample (however the intervention did increase the wealth summary score among AGYW who were out of school at baseline).

Qualitatively, five studies examined economic outcomes from five unique interventions. Burke and colleagues [70] examined economic outcomes related to the business component of their intervention in Mozambique. At round 1, they found that nearly all participants earned money and were satisfied with the amount earned. At round 2, roughly half of participants were earning money, largely due to the fact that they were repaying the intervention for loaned items related to their businesses. Berry and colleagues [36] found that the intervention increased income-generating activity knowledge among participants in Lesotho.

## Discussion

This study is the first systematic review of bundled interventions simultaneously aiming to strengthen economic and health/life skills assets for adolescents and young people in Africa. Our review was informed by a conceptual framework with HIV and STI incidence/prevalence as the primary outcomes and other secondary outcomes which are mediators of HIV risk. We reviewed 58 studies, including 43 quantitative studies and 15 qualitative studies, evaluating 26 unique interventions.

The intervention components and outcomes examined varied widely and thus were not conducive to a meta-analysis. However, the most common types of intervention bundles included the following (not mutually exclusive categories because several interventions included multiple combinations): cash plus health information (23 studies); cash plus a life skills component (22 studies); cash plus mentoring (20 studies); vocational training (e.g., hairdressing or computing) plus life skills (20 studies); vocational training plus health information (18 studies); and a savings account plus cash, fiscal literacy, and health information (14 studies).

Overall, the studies showed a greater number of significant results (more than 50%) in improving economic outcomes; mental health and psychosocial outcomes; sexual and reproductive health knowledge and services utilization; and HIV prevention knowledge and testing. They showed fewer significant results (50% or less) in improving outcomes related to HIV incidence/prevalence; sexual risk behaviours; gender-based violence; gender attitudes; education; STI incidence, prevalence and testing; and sexual debut.

These limited impacts on behaviours may be due to the complex interplay of gender norms and vulnerability surrounding these outcomes. Gendered power disparities in sexual relationships (especially those that are transactional or age-disparate) enable a tacit acceptance of violence [94] and can make it difficult for AGYW to negotiate condom use and whether or when to have sex [95]. Thus, gender norms can leave AGYW vulnerable to increased risk of HIV and other STIs, pregnancy, violence, and exploitation [96–98]. Sexual risk behaviours, gender attitudes, and gender-based violence outcomes are also more distal outcomes (relative to the intervention components), and harder to change for the aforementioned reasons than knowledge, HIV/STI testing, health services utilization, and economic well-being. Nevertheless, some interventions such as Ujana Salama in Tanzania did show promise in addressing gender equitable attitudes (with many results driven by males) [78].

Alternatively, it may be that the economic vulnerability driving HIV risk is not adequately addressed in the interventions despite improvements in the economic outcomes measured (e.g., having a savings account, amount saved, engagement in paid activities). These outcomes demonstrate marginal improvements in economic security, but it is likely that the adolescents and young people targeted remain poor, and their economic security was only partially improved by these interventions. Relatedly, many of the interventions reviewed only address economic strengthening at the adolescent/youth-level and do not address household-level economic security, a limitation noted by Austrian and Soler-Hampejsek [57] in their potential explanations for limited impacts of the AGEP intervention in Zambia. Thus, poverty as a driver of HIV risk was not fully eliminated in most of the interventions. Indeed, interventions such as those studied here cannot be expected to eliminate poverty, and our findings underscore the need for expanded, scaled programming of structural interventions such as increased access and quality of education, social protection (often most effectively targeted to households and not adolescents), and expanded formal sector employment opportunities. Most of these solutions can only be fully addressed by government policy.

Only one study examined in this review found a protective effect in delaying sexual debut [58], while two others found mixed adverse effects [48, 65] and one found fully adverse effects [57]. These mixed/adverse findings are in contrast to two studies examining cash transfers only, which found protective effects on delaying sexual debut in Kenya and Malawi [20, 99]. It is possible that the cash transfers more effectively reduced poverty through larger cash transfers sizes and longer duration of transfers, thus reducing the risk of early sexual debut, as compared to the bundled interventions. However, few studies have examined cash only versus cash plus versus control arms to effectively isolate the different impacts, and thus our conclusion on this point is only speculative.

When examining effectiveness by combinations of components, the combination with the highest percentage of protective effects (out of total categories examined), were interventions that incorporated cash plus a life skills component; cash plus a health information component; cash plus a savings account, fiscal literacy, and health information component; and cash plus a mentoring component. These combinations all had the highest percentage of protective effects for economic outcomes, but also saw a high percentage of protective effects for sexual and reproductive health, HIV prevention knowledge, and psychosocial and mental health outcomes. These findings that multisectoral interventions can improve adolescent health and well-being are supported by another study which found enhanced effects when interventions, policies, and practices (referred to as “accelerators”) are combined, creating a “simultaneous, cumulative effect across a range of outcomes” [100]. Nevertheless, many of the studies evaluating DREAMS interventions did not specify the specific combinations of components evaluated, and thus this limits our ability to draw broad conclusions about the most effective combinations.

As has been found in previous reviews, the proportion of studies examining HIV incidence or prevalence is low [13, 31], and this may be due to logistical or ethical challenges in providing adequate counselling and referral services within a study setting, the desire to avoid stigmatizing study participants, or difficulties in obtaining ethical approvals for HIV testing. It may also be due to lower HIV prevalence compared to other STIs, and thus lower implied power in impact evaluations to find impacts on HIV incidence. Thus, studies often include STI incidence and/or testing as a proxy for HIV risk . In addition, most of the studies examining HIV incidence/prevalence directly in our review (and all of which found protective trends) had weak causal identification study designs, and thus findings should be interpreted with caution. This speaks to difficulties in evaluating scaled-up programming such as DREAMS, but quasi-experimental methods such as matching or geographic regression discontinuity can sometimes be implemented to better estimate causal impacts.

In terms of geographic representation, the largest number of studies came from Uganda with 16, followed by Kenya and Tanzania, with 12 studies each. Countries from western and central Africa were underrepresented in our review, which has been previously recognized as a prevailing trend in research on HIV in Africa [31], but is also likely due in part to our review of studies published in English only.

A majority of the studies reviewed (38 in total) focused exclusively on girls and young women, which is not unexpected given their increased risk of HIV infection in the African context. However, engaging only AGYW at the individual level places the burden of change on some of the most vulnerable members of the community. In connection with the findings that interventions were less effective at changing gender attitudes, violence experiences, and sexual risk behaviours, all of which are relational outcomes (dependent upon interactions with a male partner or the community at large), it is important to consider engaging those members of the community who hold more power (e.g., caregivers, religious organizations, schools, community leaders). These findings taken together may suggest that more interventions need to target both males and females, or the larger community (which entails more complex interventions), to effect meaningful change around these complex outcomes. Programmes that focus on HIV risk behaviours through the promotion of gender equality and inclusion of boys and young men (e.g., Stepping Stones in South Africa [101]) show promising results in addressing this issue as well.

Alternatively, lack of effectiveness across certain domains may indicate problems with the way interventions are implemented, rather than with how they were designed. For example, components like cash transfers are likely to be consistently implemented across settings. However, more complex interventions like in-person trainings may vary in quality based on background qualifications of instructors as well as the amount of training they received prior to implementation. One example of how implementation characteristics may affect programme impacts comes from the Empowerment and Livelihood for Adolescents programme, which was implemented in both Uganda (with strong impacts) [102] and Tanzania (largely null impacts) [64]. In the Tanzanian case, the authors used qualitative data to understand lack of impacts and concluded this was due to lower quality implementation resulting from resource constraints and contextual factors. There is also a risk of publication bias if interventions are implemented poorly, causing null effects, and then corresponding studies may be less likely to be published. More implementation research is needed to understand how different interventions work in real-world conditions and subsequently improve programme design and delivery. Lessons can be learned to understand how to better adapt these interventions for AGYW in different contexts within Africa.

In studies which did include both males and females, some differences were found between the groups. Out of 17 studies which examined intervention impacts among males and females combined, eight did not report moderating impacts by sex. Five studies found differences by sex, with four finding more protective effects among boys than girls [48, 62, 78, 91], and the fifth finding different protective effects among the two [65]. The remaining four studies which included both males and females were qualitative, and they did not report differences in findings by sex. The finding that interventions were more effective among males than females may underscore the influence of gender norms on girls’ attitudes and behaviours and reflect the fact that gender norms create more barriers for the latter, which are harder to overcome with individually targeted interventions.

Despite many protective effects across multiple domains summarized in this review, findings were mixed, suggesting that programme design and implementation characteristics matter, as does the context where the intervention was implemented. For example, the ELA intervention was implemented in both Uganda and Tanzania, where in the former many protective effects were found and in the latter, almost no effects were found [64, 102]. Similarly, the AGI-K intervention had considerably different effects within the same country (in Kenya in Kibera as compared to in Wajir) [59, 76]. Additionally, two studies found significance at midline but not endline [38, 53] for HIV testing, knowledge, and risk perception and gender based- and sexual violence outcomes, indicating that the intervention effects may not be sustained in the medium- and long-terms. Naledi and colleagues [38] suggested that this is possibly because HIV was deprioritized among their study participants, as factors related to poverty (e.g., low educational attainment, high unemployment, and violence) can take priority over HIV risk perception. This could also be because the cultural or economic environments were too strong an influence to sustain the individual-level positive effects of the intervention. Longer intervention periods, interventions that enrol influential community members or institutions, and/or programming that that addresses both the economic and social drivers of HIV (e.g., harmful gender norms) might be more effective in the long run. In contrast, Bandiera and colleagues [53] found increased self-perception of entrepreneurial abilities and probability of self-employment only at endline, suggesting that the programmatic effects required time to develop, or possibly that as participants aged, they began to perceive their abilities and economic opportunities differently. Similarly, in the Ujana Salama intervention in Tanzania, Round 2 effects related more to intermediate outcomes such as attitudes and knowledge, whereby effects on HIV testing, violence, and other behaviors were only seen after Round 3 [48, 65, 78].

The heterogeneity across programming reviewed and outcomes examined makes it difficult to draw conclusions about the most effective designs for reducing HIV risk. Our conceptual framework can serve as a guide for future evaluations in terms of mediators to measure. Future efforts should provide guidance on preferred instruments for more consistent measurement of concepts across studies. Relatedly, most studies did not examine moderators; for example, how does service availability and readiness at existing facilities moderate programme impacts on health seeking, or how do community gender norms affect impacts on GBV? More research on how these contextual factors moderate programme impacts is needed.

There are some limitations to our review. One relates to the heterogeneous programmes examined, making definitive conclusions about the most effective combinations elusive. A second, related limitation is the heterogeneity in the outcomes measured across studies. A third limitation is that many studies were low quality in terms of ability to identify causal effects, and thus comparisons across strong and weak study designs should be interpreted with caution. In addition, for each study, we counted a category as having protective effects if any indicator in that category had a protective effect (and no mixed findings), even if impacts on other indicators in the same category were null. In this way, we may overestimate how “protective” the interventions are. A final limitation is that we only reviewed studies in English and in doing so we may have missed out on important contributions to the literature.

## Conclusion

Our review demonstrates the potential for bundled, multisectoral interventions combining health and economic strengthening for preventing HIV and facilitating safe transitions to adulthood, and findings from these studies have implications for the design of HIV sensitive programmes on a larger scale. In particular, findings support the Global AIDS Strategy 2021–2026 Results Area 9, which highlights the need for health and social protection schemes that support wellness, livelihood and enabling environments for people living with, at risk of and affected by HIV. Our findings underscore that intersectoral programming is successful at increasing SRH and HIV knowledge, testing, and related outcomes; however, mitigating risk related to relational outcomes (e.g., sexual risk behaviours, HIV incidence, and violence) remains more elusive and may require multi-level (including partners and/or the community) and not just intersectoral programming targeted to individual adolescents and youth, or also simultaneous supply-side strengthening to improve service readiness and availability. Moreover, intersectoral linkages are not only effective in producing desired outcomes related to HIV prevention but may also be effective in securing funding through co-financing, especially in an environment of diminished vertical HIV funding.

### Supplementary Information

**Additional file 1: Appendix 1. **Search Terms.

**Additional file 2: Appendix 2. **Conceptual Framework.

**Additional file 3: Appendix 3. **Outcome Categories and Indicators.

**Additional file 4. **Sum of Programmes: *Quantitative Studies*

**Additional file 5: Appendix 5.** Sum of Findings *(Qualitative Studies).*

**Additional file 6: Appendix 6. **Sexual Risk Behaviour Outcomes Sub-Chart.

**Additional file 7: Appendix 7. **List of Abbreviations.

## Data Availability

No data sets were generated or analysed during this study. However, all studies reviewed are referenced in this published article (see Table 1 for complete list of included studies).
