# Supplementary material for: Systematic review of cash plus or bundled interventions targeting adolescents in Africa to reduce HIV risk
Source: BMC Public Health. 2024 Jan 20;24:239. doi: 10.1186/s12889-023-17565-9 (PMC10799364; doi:10.1186/s12889-023-17565-9)
Supplement: Supplementary file 1 — Additional file 1: Appendix 1. Search Terms. [file 12889_2023_17565_MOESM1_ESM.docx]

**Appendix 1. Search Terms**

(adolescents OR adolescent OR adolescence OR teen OR girls OR “young people” OR “young women”) AND Africa AND (mentoring OR “behavior change communication” OR “behaviour change communication” OR “safe spaces” OR “life skills” OR “life-skills” OR “health promotion” OR “transactional sex” OR HIV OR “human immunodeficiency virus” OR “sexual health behavior” OR “sexual health behaviour” OR “risky sexual behavior” OR “risky sexual behaviour” OR “sexual health” OR “reproductive health” OR “well-being” OR “well being” OR parenting) AND (“social protection” OR “cash plus” OR “bundled intervention” OR “multisectoral programme” OR “multisectoral program” OR “multi-sectoral programme” OR “multi-sectoral program” OR “structural intervention” OR “multifaceted policy intervention” OR “multi-faceted policy intervention” OR “girls empowerment program” OR “girls empowerment programme” OR “gender transformative intervention” OR “empowerment plus” OR “cash transfer” OR “economic empowerment intervention” OR microfinance OR “productive grant” OR microcredit OR “savings account” OR voucher OR “in-kind” OR “safety nets” OR DREAMS OR “income-generating activities” OR “income-generating activity” OR “income generating activity” OR “income generating activities”)
