# Supplementary material for: Systematic review of cash plus or bundled interventions targeting adolescents in Africa to reduce HIV risk
Source: BMC Public Health. 2024 Jan 20;24:239. doi: 10.1186/s12889-023-17565-9 (PMC10799364; doi:10.1186/s12889-023-17565-9)
Supplement: Supplementary file 2 — Additional file 2: Appendix 2. Conceptual Framework. [file 12889_2023_17565_MOESM2_ESM.docx]

**Appendix 2. Conceptual Framework**

# **Conceptual Framework**

The primary outcomes of interest in our review are HIV and STI infection, and as secondary outcomes we examine pathways and risk factors (mediators), which are laid out in more detail in the Conceptual Framework (Figure 1).

***Context***

This framework illustrates the complex interplay of structural, community, household, relationship, and individual drivers influencing HIV and STI infection among adolescents. By integrating bundled interventions or “cash plus” approaches, programmes and interventions can tackle gendered vulnerabilities, provide economic empowerment, and address multiple other health and social risks. Before laying out this framework, we first provide some key definitions. Adolescence comprises a life phase of both biological growth and transitions in major social roles and is often defined as the period from 10-19 years (Sawyer et al., 2018). Nevertheless, some have argued that 10-24 years corresponds more accurately to adolescent growth and reflects social aspects of the transition to adulthood (Sawyer et al., 2018). In the current study, we focus on adolescents and young people as reflective of definitions for the age groups identified by the United Nations (ages 10-24 years). This age range also encompasses the target group (i.e., girls and young women aged 15-24 years) for increased investments identified by the Determined, Resilient, Empowered, AIDS-free, Mentored and Safe (DREAMS) partnership, based on increased risk of infection among this age group, particularly girls. The conceptual framework described below lays out hypothesized pathways through which bundled interventions may address HIV risk factors.

***Mediators of HIV risk***

Indirect and direct mediators of HIV risk can work at the structural, community, household, relationship, and individual levels. It is important to note that most government social protection programmes target households with economic strengthening components (e.g., cash transfers or asset transfers), whereas most of the bundled interventions aimed at adolescent empowerment and prevention of HIV risk tend to target adolescent girls and young women (AGYW). In our model, we illustrate hypothesized mediators at both levels.

*Household-level mediators*

At the household level, economic strengthening programmes may affect economic and food security, labour and time use decisions, stress, and intrahousehold decision-making. Economic strengthening components of bundled interventions may reduce poverty, food insecurity, and stress, as these effects have been demonstrated with social protection programmes (Bastagli et al., 2019; Hidrobo et al., 2018). Economic strengthening can result from direct cash payments, increased livelihoods capacity (through more productive agriculture, small businesses, or livestock tending). In turn, these activities may have ambiguous effects on adolescents’ engagement in labour, as studies have shown that while in general cash transfers reduce child labour (De Hoop & Rosati, 2014), they can sometimes result in children increasing participation in agricultural activities for the household (as these activities generally become more productive) (De Hoop et al., 2020), and these increases in labour may occur simultaneously with increases in school attendance (De Hoop et al., 2019). Moreover, economic strengthening can lead to adults’ increased engagement in productive activities, which in turn may increase the burden of caregiving or domestic chores among adolescents, particularly girls (Hoddinott et al., 2010).

Households facing economic insecurity may engage in decisions which increase girls’ risk of HIV infection, including marrying off girls or tacitly approving transactional relationships to support households’ basic needs (Stoebenau et al., 2016). The evidence base on cash transfers in particular and child marriage is mixed, but does suggest the potential for these programmes to reduce child marriage in some contexts (Malhotra & Elnakib, 2021; Mathers, 2021). In terms of transactional sex, the evidence is also mixed, but some studies have shown household-level cash transfers to be protective (Cluver et al., 2013; Rosenberg et al., 2014).

Food and economic insecurity at the household level may also affect time use decisions. For example, households’ improved economic standing may result in adolescents’ ability to continue schooling, and the evidence shows that cash transfers increase school attendance (Baird et al., 2014), which is protective against HIV risk (De Neve et al., 2015; Stoner et al., 2017). However, while increasing education is associated with protective behaviors (De Walque, 2009), the relationships between educational attainment and HIV risk varies depending on stage of epidemic (Iorio & Santaeulàlia-Llopis, 2016). In contrast, some household-targeted economic strengthening programmes such as public works programmes, where households may be required to work on public projects (e.g., digging wells, planting trees) to be eligible for cash payments, or programmes with intense training or work requirements for adult women might increase adolescent girls’ participation in household chores as a substitution effect for adult women’s labour (Hoddinott et al., 2010).

In terms of access to health care, cash transfers have been found to increase health seeking when ill (Bastagli et al., 2019; Novignon et al., 2022), and increase spending on healthcare (Pega et al., 2017).

*Relationship-level mediators*

At the relationship-level, risk factors for HIV include intimate partner violence, age-disparate relationships, and unequal power dynamics (which may influence condom use and sexual negotiation). These risk factors may be mitigated by economic strengthening components of bundled interventions, which reduce the need for engaging in sexual relationships with unequal power dynamics or concurrent relationships, or they may be mitigated by health/life skills components, which can increase HIV prevention knowledge, self-efficacy and behaviours such as condom use. Cash transfers generally reduce IPV risk (Buller et al., 2018), as do vocational training interventions combined with gender transformative components (Gibbs et al., 2017). Moreover, the synergies between the two components (economic and health/life skills), may also improve these outcomes, as economic strengthening interventions combined with gender transformative components have been found to increase condom use and reduce number of sexual partners (Gibbs et al., 2017).

*Individual-level mediators*

Poverty and food insecurity often drive sexual risk behaviours such as early sexual debut, participation in transactional sex, age disparate partnerships, or sex without a condom, and these behaviours can increase risk of HIV and STIs. Economic strengthening components of bundled interventions can reduce poverty and food insecurity, reducing the need to engage in these risky behaviours. Moreover, economic strengthening components such as cash transfers can increase school enrolment (Baird et al., 2014), which is protective against HIV infection (Glynn et al., 2004). Further, higher grade achievement in school has been shown to increase age of sexual debut and decrease participation in transactional sex (Dunkle et al., 2004; Uthman, 2008).

Access to information on basic sexual and reproductive health knowledge (including contraception and HIV prevention) can be improved through life skills and health components of bundled interventions. This can address lack of knowledge and increase participants’ accurate knowledge of mode of HIV transmission and protection. Relatedly, misconceptions often learned through accepted social norms regarding sexual behaviours (e.g., that condoms presuppose the user is sexually promiscuous) (Ninsiima et al., 2018) can be corrected. In turn, this increased knowledge may lead to fewer risky sexual behaviours as well as increased access to health services and, subsequently, increased contraceptive use, and HIV testing and treatment.

Economic strengthening components can improve mental health and psychosocial outcomes (Zaneva et al., 2021; Zimmerman et al., 2021), as could life skills components such as mentoring and safe spaces, which in turn may reduce the risk of risky sexual behaviours and gender-based violence, both of which are mediators of HIV risk (Pitpitan et al., 2012; Roberts et al., 2018; Wamoyi et al., 2016).

***Moderators***

There are several moderating factors that can influence the effectiveness of bundled interventions on HIV infection and related risk factors. These have largely not been empirically tested in the literature we reviewed; however, we note them as important contextual factors which can influence programme effectiveness.

At the macro-level (including regional and national levels), economic, political, or environmental shocks (such as droughts/floods or changing weather patterns) can influence the availability of resources and ability to cope with shocks. These can potentially expose adolescents to food insecurity, violence, and/or instability.

One important moderator is HIV prevalence levels in any specific context. Additionally, the availability and accessibility of health services influences whether and how an individual will seek health care, including HIV and STI testing (Kidman et al., 2020; Mavedzenge et al., 2014). Distance from a health facility, roads and infrastructure, and availability of transportation are also critical to whether an individual accesses health care. Socioeconomic inequality and stigma associated with HIV-related care also plays a role in whether an individual accesses care (Chipanta et al., 2022). Adolescent-friendly services can also have an effect on an individual’s knowledge of their HIV status, treatment options if they do test positive, and the understanding one has of sexual and reproductive health and HIV prevention in general (Hoopes et al., 2016; Rosenberg et al., 2018; Tylee et al., 2007).

At the community-level, gender attitudes and norms can influence how an individual understands certain behaviours to be acceptable, even if they might be harmful (e.g., girls getting married and pregnant at a young age, or not wearing condoms to prove fidelity to one’s partner) (Lokot et al., 2021; Ninsiima et al., 2018). Other community-level moderators include distance to and quality of education and health services, which affect educational attainment and access to healthcare. Another important barrier to HIV testing and accessing condoms is levels of HIV-related stigma and how these are internalized by individuals (Mbonu et al., 2009; Velloza et al., 2015).

Moreover, the effectiveness of interventions can be strengthened by addressing gender inequality and systemic exclusions of people with disability.


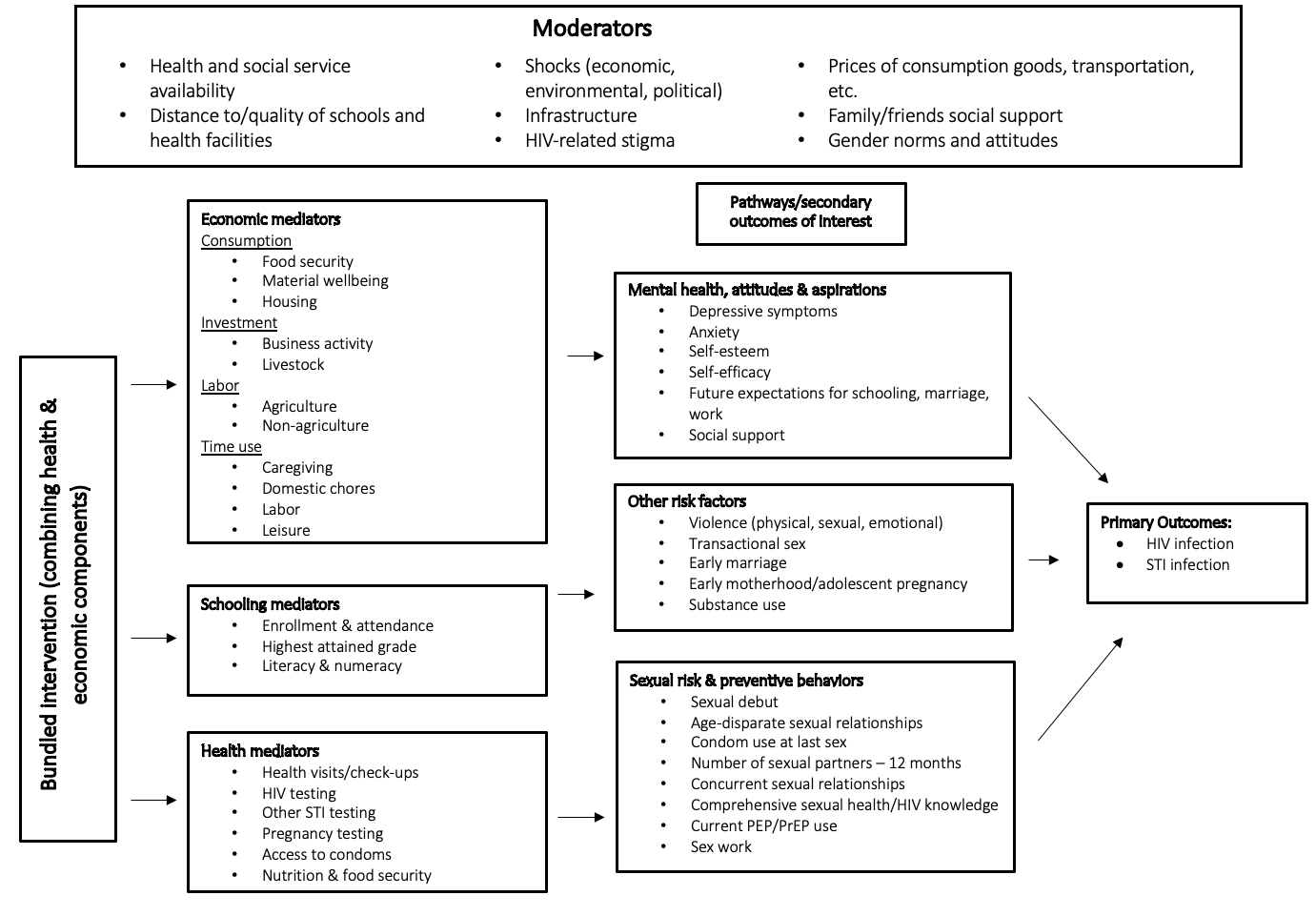


***Figure 1*. Conceptual Framework**
