# Supplementary material for: Systematic review of cash plus or bundled interventions targeting adolescents in Africa to reduce HIV risk
Source: BMC Public Health. 2024 Jan 20;24:239. doi: 10.1186/s12889-023-17565-9 (PMC10799364; doi:10.1186/s12889-023-17565-9)
Supplement: Supplementary file 3 — Additional file 3: Appendix 3. Outcome Categories and Indicators. [file 12889_2023_17565_MOESM3_ESM.docx]

| **Appendix 3. Outcome Categories and Indicators** | |
| --- | --- |
| **Outcome Categories** | **Indicators** |
| HIV incidence | HIV incidence |
| HIV testing/knowledge of status | Tested in past 12 months, knowledge of where to obtain testing |
| STI incidence/testing | Tested in past 12 months, knowledge of where to obtain testing, STI symptoms, STI incidence (e.g., HSV-2) |
| Sexual risk behaviors | Knowledge of safe sex and/or sexual risk behaviors |
| *Number of partners/concurrency* | *Number of sexual partners in the last year, multiple partners at the same time* |
| *Condom use* | *Condom use at last sex, knowledge of where to obtain condoms, likelihood of using condoms with future partners* |
| *Age-disparate partnerships* | *Age differences of 10 or more years between partners, intergenerational sex* |
| *Transactional sex* | *Non-marital sex conducted in exchange for material goods or money* |
| Sexual debut | Ever had sex, age at first sex |
| Sexual and Reproductive Health  (SRH) | SRH knowledge (including menstruation, fertility, pregnancy, and family planning), SRH health-seeking behavior, contraceptive knowledge, contraceptive use, knowledge of health facilities |
| HIV prevention knowledge | General and/or clinical knowledge of HIV, knowledge or awareness of how to prevent HIV, HIV risk factors, HIV risk perception |
| Gender attitudes | Gender norms, gender-equitable attitudes |
| Psycho-social well-being/mental health | Self-efficacy, self-esteem, self-concept, confidence, depression, hopelessness, |
| Education | Enrolment, attendance, attainment, future educational aspirations, confidence in attaining educational plan |
| Gender-Based Violence  (GBV) | Knowledge or experience of GBV (including physical violence, sexual violence, coercion, forced sex), violence prevention and/or awareness, attitudes towards violence |
| Economic | Earnings or savings, access to a savings account, fiscal literacy education, wealth creation education/knowledge, engagement in paid work, owning a business, tending livestock, income-generating activities, learning a vocational skill, economic empowerment, entrepreneurial attitudes |
