# Supplementary material for: Systematic review of cash plus or bundled interventions targeting adolescents in Africa to reduce HIV risk
Source: BMC Public Health. 2024 Jan 20;24:239. doi: 10.1186/s12889-023-17565-9 (PMC10799364; doi:10.1186/s12889-023-17565-9)
Supplement: Supplementary file 4 — Additional file 4. Sum of Programmes: Quantitative Studies [file 12889_2023_17565_MOESM4_ESM.docx]

**Sum of Programmes: *Quantitative Studies***

| **Adolescent Girls Empowerment Program (AGEP)** | | | | | | | | |
| --- | --- | --- | --- | --- | --- | --- | --- | --- |
| **#** | **Author, year** | **Sample Size** | **Study Design** | **Outcomes** | **Analysis Method** | **Duration of Follow-up (from baseline)** | **Findings** | **Causal Estimate Quality** |
| 1 | Austrian, Soler-Hampejsek, Behrman, et al., 2020 | 3200 | cRCT with 5 rounds of data collection: baseline, Round 3 (end of intervention, i.e., 2 years from baseline); Round 5 (2 years after intervention ended, i.e. 4 years from baseline). | Longer-term educational (completed grades 7 and 9) and fertility outcomes (15+, ever had sex, ever been pregnant, ever given birth, ever been married). Subset of hypothesized mediating indicators: social assets (self-efficacy, safe space, positive gender attitudes, non-acceptability of IPV; economic assets (financial literacy, whether or not saved money in last year); health assets (knowledge of fertile period and contraceptive methods, HIV knowledge - if ever sex, included condom use, transactional sex). | ITT approach for outcomes measured at baseline, R3 and R5. DID comparing changes between rounds and intervention/control. Secondary analysis of TOT conducted to account for potential differential programme participation. ITT was re-estimated for urban vs. rural, younger vs. older and highest vs. lowest vulnerability. | 4 years (2 years after programme ended) | Round 3: programme improved outcomes relative to the control at Round 3 including: 1) having a safe space in the community to meet with friends increased 8.2 percentage points (CI 1.7–14.6), 2) financial literacy score increased 0.24 points out of 9 (95% CI 0.01–0.48), whether girls had saved money in the past year increased 6.7 percentage points (pp) (CI 1.8–11.5) and sexual and reproductive health knowledge increased 0.289 points out of 11 (CI 0.095–0.483). Round 5: intervention increased probability of saving money by 6.7 pp (95% CI 2–11.3); sexual and reproductive health knowledge by 0.268 points out of 11 (CI 0.06–0.48); self-efficacy by 0.31 points out of 10 (95% CI 0.02–0.6); probability of ever having had sex by 6.9 pp (CI 1-12.7) and reduced the probability of having transactional sex by 11.8 pp (CI 12.19 to -1.7). Programme impacts not observed for girls’ HIV knowledge, condom use, gender norms, grade completion, marriage, pregnancy, or fertility. Few differences between age cohorts: self-efficacy was 0.49 points out of 10 higher for older girls (95% CI − 0.02–1) and non-acceptability of IPV was 11.7 percentage points higher for older girls (95% CI -0.5–23.9). Girls in intervention arm in urban sites were 13.5 percentage points (CI 3.3-23.7) more likely to have sexually debuted than those in rural areas. The poorest girls in the treatment arm were 16.2 pp more likely to be married (CI 0.8-31.7), 21.3 pp more likely to have given birth (CI 7.1-35.4), and 28pp more likely to have been pregnant (CI 14.1-41.8). | High |
| **Adolescent Girls Initiative-Kenya (AGI-K)** | | | | | | | | |
| **#** | **Author, year** | **Sample Size** | **Study Design** | **Outcomes** | **Analysis Method** | **Duration of Follow-up (from baseline)** | **Findings** | **Causal Estimate Quality** |
| 2 | Austrian, Soler-Hampejsek, Kangwana, et al., 2021 | Kibera: 2390  Wajir: 2147 | RCT in Kibera /cRCT in Wajir | Experienced violence by a male in past year, contraceptive knowledge and use, marriage; school enrollment, grades attained, primary school completion, transition to secondary school, gender equitable attitudes, gender equitable schooling attitudes, knowledge of menstrual cycle, SRH knowledge, self-efficacy, condom self-efficacy, financial literacy, savings | 4 arms: 1) Violence prevention (comparison); 2) violence and education; 3) violence, education, and health; 4) violence, education, health, wealth. Baseline: assessed balance and analyzed attrition using OLS. Baseline survey prior to start of intervention and 2 -year follow-up. ITT impacts estimated for each nested combination of single-sector interventions relative to the Violence-only arm using OLS ANCOVA models. All scale and summative score were converted into z-scores. | 2 years | All findings summarized are impacts of the intervention arm compared to the violence-only (comparison arm). In Kibera, violence + education reduced violence experiences by 8.8pp (CI -0.14, -0.03) and increased general self-efficacy z-score by 0.152 (CI 0.06, 0.25) but had no effects on gender equitable attitudes, grade attainment, primary school completion, enrolment, transition to secondary school, fertility and contraceptive knowledge, condom self-efficacy, wealth creation, or savings, violence summary index, education summary index, health summary index, or wealth summary index. Violence+education+health reduced violence experiences by 5.9pp (CI -0.12, -0.00), increased contraceptive knowledge by 13pp (CI 0.07, 0.19), increased SRH knowledge z-score by 0.213 (CI 0.09, 0.34), increased condom self-efficacy z-score by 00.178 (CI 0.05,0.30), and increased health outcomes summary index z-score by 0.306 (CI 0.19, 4.42) but had no effects on no effects on gender equitable attitudes, grade attainment, primary school completion, enrolment, transition to secondary school, fertility knowledge, self-efficacy, wealth creation, or savings, violence summary index, education summary index, or wealth summary index. The violence+education+health+wealth intervention arm increased grade attainment by 0.067 grades (CI 0.01, 0.12), increased the transition to secondary school by 7.1 pp (CI 0.01, 0.13), increased completion of primary school by 7 pp (0.02, 0.12), increased knowledge of modern contraceptive methods by 11.9 pp (0.06, 0.18), increased SRH z-score by 0.158 (CI 0.03, 0.29), increased the financial literacy z-score by 0.381 (CI 0.26, 0.50), increased the likelihood of savings by 20.2 pp (CI 0.14, 0.26), increased the education outcomes summary z-score by 0.123 (CI 0.03, 0.22), increased the health outcomes summary z-score by 0.279 (CI 0.16, 0.40), increased the wealth outcomes summary z-score by 0.517 (CI 0.40, 0.63) but had no effects on violence, primary school completion, enrolment, fertility knowledge, self-efficacy, or the violence summary index.  In Wajir, violence + education increased gender schooling attitudes by 0.156 (CI 0.00, 0.31), increased grade attainment by 0.259 grades (CI 0.10, 0.42), increased probability of enrollment by 14.4 pp (CI 0.09, 0.20), increased SRH knowledge z-score by 0.344 (CI 0.08, 0.60), and increased the education outcomes summary z-score by 0.302 (CI 0.16, 0.44) but had no effects on violence, gender equitable attitudes, fertility knowledge, contraceptive knowledge, self-efficacy, financial literacy, saving, violence prevention summary index, health summary index, or wealth summary index. The Violence+education+health arm reduced gender equitable attitudes by 0.208 (CI -0.39, -0.02), increased grade attainment by 0.194 grades (CI 0.02, 0.36), increased enrollment by .07 pp (0.01, 0.13), reduced contraceptive knowledge by 10.3 pp (-0.19, -0.01), increased SRH z-score by 0.433 (CI 0.17, 0.70), increased probability of savings by 4.7 pp (CI 0.01, 0.09), increased the education outcomes summary z-score by 0.189 (CI 0.02, 0.36, and increased the wealth outcomes summary z-score by 0.349 (CI 0.03, 0.66) but had no effects on violence, positive gender schooling attitudes, fertility knowledge, self-efficacy, financial literacy, violence prevention summary index, or health summary index. The violence+education+health+wealth intervention arm increased grade attainment by 0.181 grades (0.01, 0.35), increased enrollment by 8.4 pp (CI 0.02, 0.14), increased savings by 40.9 pp (CI 0.31, 0.51), and increased the wealth creation outcomes index z-score by 2.852 (CI 2.14, 3.56) but had no effects on violence, gender equitable attitudes, positive gender schooling attitudes, fertility knowledge, SRH knowledge, self-efficacy, financial literacy, violence prevention summary index, or health summary index. | High |
| 3 | Austrian, Soler-Hampejsek, Kangwana, et al., 2022 | 2147 | prosepective cRCT | Primary: ever married, ever pregnant, ever given birth, fertility outcomes summary index. Secondary: violence prevention summary index, education outcomes summary index, health outcomes summary index, wealth creation outcomes summary index | 4 arms: 1) Violence prevention (comparison); 2) violence and education; 3) violence, education, and health; 4) violence, education, health, wealth. Baseline: assessed balance and analyzed attrition using OLS. Estimated ITT effect of each package of interventions relative to the Violence-only study arm using ANCOVA models. School enrollment was moderately imbalanced across study arms at baseline, so authors carried out a post-hoc subgroup analysis for girls enrolled versus not enrolled in school at baseline. | 4 years | There were no impacts of any of the study arms in the full sample on primary outcomes including ever married, ever pregnant, ever given birth, fertility outcomes summary index. However, among those who were out of school at baseline, the violence education arm reduced the probability of ever having been married by 24.3 pp (CI -0.39, -0.10) and every being pregnant by 18.2 pp (CI -0.33, -0.03) and reduced the fertility summary z-score by -0.471 (CI -0.77, -0.17), but had no effects on ever given birth, fertility summary index, health outcomes summary index, or wealth creation summary index. The violence education + arm did not have any impacts among this sub-sample. The violence + health + education + wealth arm reduced the probability of ever having been married by 16.8 pp (CI -0.33, -0.00) and reduced the fertility summary z-score by -0.345 (CI-0.67, -0.02) in the subsample of girls out of school at baseline but had no impacts on ever pregnant, ever given birth. In terms of secondary outcomes in the full sample, the violence education arm increased the education outcomes summary z-score by 0.193 (CI0.09, 0.29) but had no effects on violence prevention summary index, health outcomes summary index, wealth creation index. The violence education + health + wealth arm increased the education outcomes summary z-score by 0.111 (CI 0.01, 0.22) and the wealth creation summary z-score by 0.263 (CI 0.02, 0.51) but had no impacts on violence prevention summary or health outcomes summary. The violence + education + health arm did not have any significant impacts on secondary outcomes at the 10% level. Among secondary outcomes in the sub-sample of girls out of school at baseline, the violence education arm increased the education outcomes summary index z-score by 1.103 (CI0.78, 1.43) but had no impacts on the education, health or wealth summary scores. Among this sub-sample, the violence education + health arm reduced the wealth creation summary z-score by 0.340 (CI –0.62, -0.06) but had no impacts on the violence, education, or health summary scores, while the violence education + health + wealth arm increased the education outcomes summary z-score by 0.748 (CI 0.41, 1.09) and the violence prevention outcomes summary z-score by 0.378 (CI 0.13, 0.63) but had no impacts on the health or wealth creation scores. | High |
| 4 | Kangwana, Austrian, Soler-Hampejsek, 2022 | n=2,075 | mixed methods RC T(individual-level randomization), with qual interviews longitudinally. There were three treatment arms: violence + education, violence + education + health, and violence + education + health + wealth creation. Impacts are compared to the violence only arm (control). | ever had sex, ever pregnant, ever given birth, HSV-2, fertility summary score, violence prevention summary score, education summary score, health summary score, wealth creation summary score, | Intent to treat estimates with ANCOVA models (linear probability models) and additional controls for baseline schooling, cognitive skills, parental characteristics and household wealth to improve precision and account for any initial imbalance. Examined various treatment arms separately and combined as one v. violence-only arm. | 4 years from baseline | Qualitatively, respondents reported that girls were likely to have sex as a result of child sexual exploitation, peer pressure or influence from the media, as well as for sexual adventure and as a mark of maturity. There were no impacts of any treatment arm in the full sample (ages 15-19) on fertility outcomes (index, had sex, ever pregnant, ever had child) or violence prevention outcomes. In the full sample, the violence + education arm increased the education outcomes summary score by 0.175 (CI 0.07, 0.28) and the wealth creation summary score by 0.158 (CI0.04, 0.28) but had no impacts on violence prevention, health summary. The violence education + health arm increased the health outcomes summary (b=0.187, CI 0.07-0.31) but had no impacts on education, violence prevention or wealth summary scores. The violence education + health + wealth arm increased the health (b=0.126, CI 0-0.25) and wealth (b=0.407, CI 0.29-0.53) but had no impacts on the violence prevention score. The combined treatment estimate increased the education outcomes index by 0.126 (CI 0.03-0.22), the health outcomes index by 0.136 (CI 0.04-0.24), and the wealth creation index by 0.225 (CI 0.12, 0.33). In stratified analyses by age, the following impacts were seen among those 13-14 years at baseline: the violence education arm reduced the probability of ever having sex by 8.2pp (CI -0.16, -0.01), reduced the probability of being HSV-2 positive by 7.5pp (CI -0.14 ,-0.01), reduced HSV-2 incidence (b=-0.056, CI -0.11, -0.00), increased the education outcomes summary index (b=0.243, CI 0.09, 0.40), increased the wealth creation outcomes summary index (b=0.198, CI 0.02, 0.37) but had no effects on ever pregnant, ever given birth, fertility summary, violence prevention summary, or health prevention summary; the violence education + health arm increased the health outcomes summary index (b=0.187, CI 0.07, 0.31) but had no effects on ever had sex, ever pregnant, ever given birth, fertility summary, HSV-2 positive, HSV-2 incidence, violence prevention summary, education summary, or wealth creation summary; the violence education + health + wealth arm increased the education summary index (b=0.159, CI 0.00, 0.32) and the wealth creation summary index (b=0.367, CI 0.20, 0.54) but had no impacts on ever had sex, ever pregnant, ever given birth, fertility summary index, HSV-2 positive, HSV-2 incidence, violence prevention summary, or health summary. | High |
| **Biruh Tesfa (Bright Future)** | | | | | | | | |
| **#** | **Author, year** | **Sample Size** | **Study Design** | **Outcomes** | **Analysis Method** | **Duration of Follow-up (from baseline)** | **Findings** | **Causal Estimate Quality** |
| 5 | Erulkar, Ferede, Girma, et al., 2013 | 601 at baseline, 571 at endline; (1172 interviews, but not unique) | Repeat cross-sectional population-based surveys in treatment and comparison kebeles. Baseline surveys took place in Addis Ababa, Bahir Dar, and Gondar, but endline surveys were only implemented in Gondar (2 treatment kebeles and 1 comparison kebele). | social support, HIV knowledge, voluntary testing and counselling (VCT) | Data were weighted by number of eligible girls in household to correct for selection probability. Multivariate analysis at endline examining difference between treatment and comparison. | 30 months | At endline, girls in treatment areas were more likely to have social support (OR=1.18, CI 1.07-1.31), have high HIV knowledge (OR=1.93, CI 1.23-3.02), were more likely to know where to get VCT (OR=1.39, CI 1.25-1.54), and were more likely to want VCT (OR=1.35, CI 1.21-1.50). | Low |
| **Bridges to the Future & BridgesPLUS** | | | | | | | | |
| **#** | **Author, year** | **Sample Size** | **Study Design** | **Outcomes** | **Analysis Method** | **Duration of Follow-up (from baseline)** | **Findings** | **Causal Estimate Quality** |
| 6 | Kivumbi, Byansi, Ssewamala, et al., 2019 | n=789 (control n=273; treatment n=516) | RCT (individual-level) with two treatment arms (combined for analysis purposes) and a control arm. The control arm received usual care services for orphaned children. | self-concept,, depression, and hopelessness | Estimated combined treatment effects using multiple linear regression; reported adjusted risk difference. | baseline, 12, and 24 months post-intervention initiation | Compared to controls, treatment adolescents reported a reduction in depressive symptoms (b = − 1.262, 95% CI − 2.476, − 0.047), at wave II and a 0.645-point reduction at 24-months p (b = − 1.907, 95% CI − 3.192, − 0.622), increased self-concept at Wave II (b = 3.503 (95% CI 1.469, 5.538) but not at Wave III. There were no impacts on hopelessness at either wave. | High |
| **DREAMS (to include Sauti Project)** | | | | | | | | |
| **#** | **Author, year** | **Sample Size** | **Study Design** | **Outcomes** | **Analysis Method** | **Duration of Follow-up (from baseline)** | **Findings** | **Causal Estimate Quality** |
| 7 | Birdthistle, Kwaro, Shahmanesh, et al., 2021 | 5-6,000 AGWY annually in South Africa; 8,515-11,428 in Kenya | (observational) longitudinal prospective cohort using Demographic Surveillance Site (DSS) Data | HIV incidence | Poisson regression among residents aged 15 to 24 years who had at least 2 HIV test results, with the first HIV-negative test before the age of 25. Analyses compared the 5-year period immediately prior to rollout (2011 to 2015). The DREAMS scale-up period was monitored up to 3 years after DREAMS interventions were introduced (2016 to 2019). Poisson regression was used to estimate rate ratios (RRs) and 95% CIs for the effect of calendar period on HIV incidence, overall and separately by age group (age 15 to 19 years and 20 to 24 years). | 3 years | In the DREAMS implementation phase (2016 to 2018), age-adjusted RR (aRR) for HIV incidence were 0.62 (CI 0.48 to 0.82]) among 15-19 year olds as compared to the time period 2011-2015 in South Africa. Incidence estimates were not statistically significant among 20- to 24-year-olds in South Africa, nor among any age group in Kenya. | Low |
| 8 | Birdthistle, Carter, Mthiyane, et al., 2022 | 1081 in  Nairobi slum settlements, and 2174 aged 13–22  years in rural KwaZulu-Natal | (observational) longitudinal prospective cohort using Demographic Surveillance Site (DSS) Data | knowledge of HIV status | Propensity score logistic regress (to estimate a counterfactual for DREAMS beneficiary, defined as receiving an invitation regardless of uptake). Propensity score adjustment approach was applied within age groups (15-19 and 20-24), as well as overall, with a formal test for effect modification by age group. | 1 year | In Nairobi, knowledge of HIV status was higher among DREAMS beneficiaries compared with non-beneficiaries (OR=6.98; 95% CI 4.84, 10.47 among full sample; OR=8.26; CI 5.46, 13.83 among those 15-17 years; OR=5.93 (CI3.16, 13.34 among those 18-22 years). In KwaZulu-Natal, knowledge of status was higher among DREAMS beneficiaries aged 13–17 years (OR=1.52; CI 1.26, 2.00), and there were statistically significant effects among those aged 18–22 years. | Medium |
| 9 | Chabata, Hensen, Chiyaka, et al., 2021 | 2431 | non-randomized "plausibility" evaluation | Primary outcome: HIV incidence rate by study group and site; secondary outcomes by study group and site: knowledge of HIV status, ever taken PREP, ability to negotiate condom use, knowledge of HIV status of partner, condom-less sex with regular partner, condom-less sex with client, accessed STI treatment, food insecurity, selling sex, ability to decline sex, experience of GBV from partner, violence from police | ITT estimates compared cohorts recruited in the two DREAMS cities and four non-DREAMS towns regardless of programme uptake. Impacts on HIV incidence was examined using Poisson regressions controlling for individual-level factors and community-level HIV prevalence. For secondary outcomes, authors used logistic regression and controlled for confounders due to non-randomized design. | 12 months and 24 months from baseline | There were no programme impacts on HIV incidence in fully adjusted model. DREAMS increased the odds of ever taking PREP (OR 63.82, CI 19.78-205.90), ability to negotiate condom use with partner (OR 3.39, CI 2.24-5.14), knowledge of HIV status of partner (OR 1.38, CI 1.03-1.81) and reduced the odds of condom-less sex with regular partner (OR 0.72, CI 0.53-0.98), condom-less sex with a client (OR 0.58, CI 0.38-0.89), and number of sex partners being more than 3 (OR 0.66, CI 0.50-0.87). DREAMS had no impact on knowledge of HIV status, food insecurity, selling sex, ability to decline sex, or experience of violence from police. | Low |
| 10 | Floyd, Mulwa, Magut, et al., 2022 | N=1081 (Nairobi, Kenya)  N=2184 (South Africa)  N=1171 (Gem, Kenya) | Quasi-experimental design using propensity-score matching | knowledge of HIV status, condomless sex (in past 12 months), lifetime sex, transactional sex (in past 12 months), and awareness and use of condoms and pre-exposure prophylaxis | Multivariable logistic regression model with inverse probability weighting for propensity to be invited to DREAMS and then simulations were run with propensity score matching to estimate marginal percent difference in average predicted probabilities in scenarios where 1) all respondents were DREAMS beneficiaries and 2) no respondents were DREAMS beneficiaries | 1-2 years (depending on year of enrollment) | DREAMS had positive impacts on all age groups in all 3 settings on knowledge of HIV status: Nairobi OR=5.1 (CI 3.4 to 7.6), Gem OR=1.9 (CI 1.5 to 2.5), uMkhanyakude OR=1.4 (CI 1.1 to 1.7). There were no effects on transactional sex. Effects on condomless sex, more than 1 lifetime partner, and more than 2 lifetime partners were largely not significant, with some exceptions by age-location group (but no significant effects in South Africa). For example, DREAMS reduced condomless sex among 18-22 year olds in Nairobi who were sexually active (OR=0.2, CI 0.07 to 0.8), reduced the odds of more than 1 lifetime partner in Gem overall (OR=0.7, CI 0.5 to 0.9), and reduced the odds of more than 2 lifetime partners in Gem overall (OR=0.6 CI 0.4 to 0.9) and among those 13-17 years in Gem (OR=0.3, CI 0.1 to 0.6). | Medium |
| 11 | Gourlay, Floyd, Magut, et al., 2022 | n=3582 (Nairobi n=852; Gem n=1018; uMkhanyakude n=1712) | Observational study design randomly selected participants for survey from demographic surveillance sites in Kenya and South Africa and interviewed them in 2017 and 2019, asking whether they were invited to participate in DREAMS. | social support, self-efficacy, aspirations, expectations (on education, employment, marriage, having children) | Conducted multivariable logistic regression examining self-reports of invitation to join DREAMS as independent variable and ran simulations on each outcome in 2018 and 2019 by comparing counter-factual scenarios in which all, vs no, AGYW were DREAMS invitees. | 2017-2019 (2 yrs - 3 years from outset of DREAMS study) | In 2018, AGYW invited to participate in DREAMS had higher levels of social support in Nairobi (OR=1.5, CI 1.1-2.1), GEM (OR=2.0, CI 1.6-2.6), and uMkhanyakude (OR=1.4, CI 1.1-1.7) and higher self-efficacy in uMkhanyakude (OR=1.4, CI 1.2-1.8). Stratified by age group in 2018, there were positive associations with social support in Nairobi among those 15-17 years (OR=24, CI 1.5-3.9) but not 18-22 years; and positive associations with both age groups in Gem [13-17 years: OR=2.0, CI 1.4-2.8; 18-22 years: OR=2.4 CI 1.6-3.6)] and uMkhanyakude Gem [13-17 years: OR=1.3, CI 1.4-1.7; 18-22 years: OR=1.4 CI 1.1-1.9)]. In 2018, there were no impacts on self-efficacy in Nairobi or Gem. In 2019, AGYW invited to participate in DREAMS had higher levels of social support in Nairobi (OR=1.4, CI 1.0-1.9) and GEM (OR=1.4, CI 1.0-1.8), but not in uMkhanyakude and higher self-efficacy in uMkhanyakude (OR=1.43, CI 1.0-1.5). Stratified by age, these associations were significant among those 15-17 years (OR=1.4, CI 1.0-1.8) and 18-22 years (OR=1.5, CI 1.1-2.1). In 2019, there were no impacts on self-efficacy in Nairobi or Gem in any age group. | Low |
| 12 | Govender, Beckett, Reddy, et al., 2022 | N=10384 (Gauteng province)  N=7912 (KwaZulu-Natal province) | Cross-sectional survey | HIV prevalence; secondary variables: HIV testing and antiretroviral therapy uptake, pregnancy, sexually transmitted infection, intimate partner violence, and age-disparate sex) | Parents/caregivers and adolescents were asked about exposure to "DREAMS-like" interventions to create a parent/caregiver exposure index variable (0-3 indicating 3 or more). Multiple logistic regression analyses assessed the association between DREAMS-like intervention uptake and HIV outcomes, adjusting for controls (education, race and ethncity, relationship status, having been away from home for more than amonth in previous year, repeating a grade). | 12 months | There was no association between DREAMS-like interventions and HIV prevalence, STI prevalence, number of partners, age-disparate sex, intimate partner violence, or pregnancy. Participants exposed to 2 interventions were more likely to takeup ART (OR=1.50, CI 1.04 to 2.16), have been tested for HIV (OR=1.42 (CI 1.13 to 1.63), have more HIV knowledge (OR=1.26, CI 1.10 to 1.44), and have used condoms (OR=1.29, CI 1.07 to 1.55). Participants exposed to 3 or more interventions were more likely to have been tested for HIV (OR=2.39, CI 2.11 to 2.71), have more HIV knowledge (OR=1.22, CI 1.08 to 1.38), and have used condoms (OR=1.68, CI 1.33 to 2.13). Participants exposed to only one intervention were more likely to have been tested for HIV (OR=1.26, CI 1.10 to 1.43). | Low |
| 13 | Kuringe, Christensen, Materu, et al., 2022 | N=2720 | cRCT (15 treatment, 15 control); pairs matched on urban/rural and high/low HIV prevalence and then randomized. | Incidence of HSV-2 as a proxy for risky sexual behavior | Cox proportional hazards model with random effects used to examine impacts on HSV-2 incidence, and log-binomial model used to examine impacts on other outcomes | 6, 12, 18 months | The intervention increased savings (OR=1.87, CI 1.69 to 2.08) and use of biomedical services (OR=2.10, CI 1.95 to 2.26). There were no effects on sex work, sexual partner violence, number of sexual partners, condom use, transactional sex, intergenerational sex, compensated sex, or HIV prevalence, or HSV-2 incidence. | High |
| 14 | Mathur, Heck, Kishor Patel, et al., 2022 | 1247 AG (Kenya: 389, Malawi: 371, Zambia:  487) and 1628 YW (Kenya: 347, Malawi: 883, Zambia:  398) | Pre/post, (Observational) Prospective cohorts with two time points (Kenya: 2016/2017, 2018; Malawi: 2017, 2018; Zambia: 2016/2017, 2018) | HIV testing, sexually transmitted infection (STI) symptom experience, number of sexual partners, condom use (consistently, at last sex), transactional sex, experience of physical violence (from intimate partners) and sexual violence (from intimate partners and strangers/non-partners). | Generalised estimating equations (with a Poisson distribution) to examine temporal changes, controlling for characteristics (study site and marital, schooling and orphanhood status at round 1). | 24 months | Kenya: intervention was associated with lower sexual IPV among 15-19 (IRR=0.32, CI 0.18-0.56) and 20-24 age groups (IRR=0.51, CI 0.34-0.75); associated with reduced non-partner sexual violence among the 20-24 years (IRR=0.44, CI 0.32-0.61); increased HIV testing among 15-19 years (IRR=1.18, CI 1.02-1.37), reduced consistent condom use among those 20-24 years (IRR=0.57, CI 0.40-0.81), and increased transactional sex (IRR=1.73, CI 1.04-2.89) among those 20-24 years but had no impacts on STI symptoms, number of sexual partners. Malawi: intervention increased HIV testing among 15-19 (IRR=1.11, CI 0.95-1.29) and 20-24 year age groups (IRR=1.11, CI 1.01-1.22); reduced STI symptoms in 20-24 year age group (IRR=0.69, CI 0.60-0.81); reduced sexual IPV in 15-19 years (IRR=0.35, CI 0.21-0.57) and 20-24 years (IRR=0.27, CI 0.20-0.36), and reduced physical IPV among those 20-24 years (IRR=0.68, CI 0.55-0.85) but had no impacts on number of sex partners, consistent condom use, condom used at last sex, or transactional sex. Zambia: increased HIV testing among 15-19 years (IRR=1.56, CI 1.34-1.82) and 20-24 years (IRR=1.13, CI 0.99-1.28) and reduced non-partner sexual violence among those 20-24 years (IRR=0.60, CI 0.44-0.82) but had no impacts on number of STI symptoms, number of sex partners, consistent condom use, condom used at last sex, or violence perpetrated by intimate partner. | Low |
| 15 | Mthiyane, Baisley, Chimbindi et al., 2022 | N=2016 | Cohort study | Risk of acquiring HIV (measured as incident HSV-2 and incident HIV) and the risk of sexually transmitting HIV (measured as being HIV positive with a dectectable viral load) | Poisson regressions models used to examine effects on HSV-2 and HIV; simulations run to compare predicted probability of prevalence in 1) full exposure to DREAMS and 2) no exposure to DREAMS. | Followed up annually for 2 years | There were no effects of DREAMS on HSV-2 or HIV incidence, or on transmissible HIV prevalence. | Low-medium |
| 16 | Mulwa, Osindo, Wambiya, et al., 2021 | n=1081 | Quasi-experimental using propensity score matching with a prospective cohort; exposure is based on invitation to participate and invitations were targeted to the most vulnerable | school attendance and educational attainment | Multivariable logistic regression analysis and second set of models employ propensity score matching (PSM) to account for selection bias in exposure to treatment. | 24 months | In PSM models there were no impacts on any of the outcomes examined. | Medium |
| 17 | Pelletier, Derado, Maoela, et al., 2022 | n=53,146 (n=29141 DREAMS districts, n=24005 non-DREAMS districts) | Observational | HIV incidence (direct testing) | Poisson log-linear regression models to examine differences in HIV incidence by DREAMS v. non-DREAMS districts. | 4 years (2020 f/u from 2016 start) | Among AGYW in DREAMS districts, the percentage of new HIV diagnoses decreased from 11.4% in the first quarter of fiscal year 2016 to 3.3% in the second quarter of fiscal year 2020, for a total reduction of 71.4% (p<0.001). In non-DREAMS districts, HIV incidence declined from 7.7% to 4.0% over the same period ( 48.4% decline; p<.001). The difference in the reduction between DREAMS v. non-DREAMS districts was significant (p=0.002). | Low |
| 18 | Van Heerden, Sausi, Oliver, et al., 2020 | n=126 (Dreamers n=73; non-Dreamers n=53) | (Observational) cross-sectional design; AGYW selected from implementing partner intervention lists | self-esteem, hope, self-efficacy, confidence to resist pressure to have sex, number of sexual episodes past three months | Logistic regression and chi-squared tests. | 8 months from baseline | AGYW exposed to DREAMS reported lower levels of sexual risk (OR 1.28, CI1.01–1.63) and higher levels of self-efficacy (OR 0.85, CI 0.78–0.93). Those exposed to DREAMS were also more likely to have savings (p<0.05) and a plan for how to spend the money they earn (p <0.05). There were no associations with DREAMS and the following outcomes: self-esteem, hope, caregiver’s parenting scale. | Low |
| 19 | Wambiya, Gourlay, Mulwa, et al., 2023 | N=852 (Nairobi)  N=1018 (Gem)  N=1712 (KZN) | Quasi-experimental prospective cohort study | Emotional, physical, and sexual violence | Multivariable propensity score-adjusted logistic regression | 1-2 years | There were no effects of DREAMS on violence outcomes in any setting. | Low |
| **Empowerment and Livelihood for Adolescents (ELA)** | | | | | | | | |
| **#** | **Author, year** | **Sample Size** | **Study Design** | **Outcomes** | **Analysis Method** | **Duration of Follow-up (from baseline)** | **Findings** | **Causal Estimate Quality** |
| 20 | Bandiera, Buehren, Burgess, et al., 2020 | Endline: 3522 (midline: 4888; baseline: 5966) | cluster RCT with 100 treatment and 50 control communities | **economic empowerment:** entrepreneurial index, income-generating activity, employment, spending, empowerment index **body control:** (early) childbearing, (child) marriage, forced sex, condom use, contraceptive use, pregnancy knowledge; **aspirations:** perceptions of gender roles, aspirations related to marriage and child bearing | Surveys conducted at baseline, midline (2 yrs), and endline (4 yrs). ITT impacts for core analysis, estimated using OLS ANCOVA specification. | 2 and 4 years | The paper presents two sets of ITT estimates: overall and Lee bounds. In our summary, we present the Lee bounds estimates because the authors provided standard errors (SE) in the tables for the Lee bounds estimates but not for the overall ITT estimates. Both estimates are very similar. In terms of economic outcomes, the intervention increased self- perceived entrepreneurial abilities by 5.76 (SE 2.17) at midline (effects not significant at endline), increased the probability of engaging in any income-generating activities by 7pp (SE 0.19) at midline (SE 0.024) and 5pp at endline, increased the probability of being self-employed by 6pp (SE 0.013) at midline (not significant at endline), increased expenditures on goods by 4,972 UGX (SE1357) at midline (not significant at endline). In terms of the “control over the body domain, the intervention reduced the probability of marriage/cohabiting by 7.1pp (SE 0.018) at midline and 8.2pp (SE 0.032) at endline, reduced the probability of “unwilling sex” by 7.1 pp (SE 0.024) at midline (not significant at endline), increased the pregnancy knowledge index score by 0.058 (SE 0.026) at midline (not significant at endline), increased the HIV knowledge index by 0.507 (SE 0.079) at midline (not significant at endline), increased the probability of always using a condom by 19.4pp (SE 0.03) at midline and 8.9pp (SE 0.039) at endline, and increased the control over the body index by 0.54 (SE 0.052) at midline and 0.265 (SE 0.06) at endline. In terms of aspirations, the intervention increased the gender empowerment index by 2.63 (SE 1.26) at midline (not significant at endline), increased the perceived suitable age for a woman to be married by 0.826 years (SE 0.145) at midline (not significant at endline), increased the perceived suitable age for a man to be married by 0.747 years (SE 0.198) at midline (not significant at endline), reduced the preferred number of children by 0.296 (SE0.089) at midline (not significant at endline), increased perceptions of the suitable age for women to have a first child by 0.681 years (SE0.168) at midline (not significant at endline), increased preferred age for daughters to get married by 0.749 years (SE 0.126) at midline (not significant at endline), and increased the aspirations index by 0.29 (SE 0.055). The intervention had no impacts on the following outcomes at either round: wage employment, has children, contraceptive use, preferred age at which sons get married. | High |
| 21 | Buehren, Goldstein, Gulesci, et al., 2017 | n=5454 at baseline; f/u n=3179 | cRCT comparing control (no intervention), core ELA "club only" intervention only, and core ELA "club" + microfinance | likelihood of being involved in any earning activity, income, plans for starting new activity or financial skills, having savings (general, at home, at ROSCA), amount of savings, having a loan, knowledge of safe sexual practices and productive health, fertility preferences, perceptions of gender roles and control over life | 1) ITT effects with individual fixed effects using linear regression with treatment arm x follow-up interaction and 2) 2-stage estimation using treatment village residence as instrument to predict club participation. | approximately 24 months | In ITT models, the programme did not have impacts on any of the key economic outcomes with the exception of one: the treatment arm with club + microfinance led to a 2.8pp increase in the probability of having savings at a ROSCA (se=0.012). However, in terms of social outcomes, ITT models indicated that the club+MF arm increased the probability of talking about business with a friend by 6.6pp (se=0.028). In instrumental variables (IV) models, the treatment arm with club participation + microfinance led to increase in having savings (b=0.572, SE0.287), ln(savings amount) (b=5.645, se 2.992), having savings at a ROSCA (b=0.194, se=0.095), and having savings at home (b=0.453, se=0.252). Also in IV models, in terms of social outcomes, the club + microfinance arm increased the perceived gender role index by 45.951 (se=18.465) and the probability of talking about business with a friend by 55.3pp (se=0.241). There were no impacts on likelihood of being involved in any earning activity, income, plans for starting new activity or financial skills, amount of savings, having a loan, knowledge of safe sexual practices and reproductive health, fertility preferences, perceptions of gender roles and control over life. | High |
| **Girl Empower** | | | | | | | | |
| **#** | **Author, year** | **Sample Size** | **Study Design** | **Outcomes** | **Analysis Method** | **Duration of Follow-up (from baseline)** | **Findings** | **Causal Estimate Quality** |
| 22 | Özler, Hallman, Guimond, et al., 2020 | n=11176 (control n=383 girls; GE n=393 girls; GE+ n=400 girls) | cRCT (village-level) | Primary outcome: sexual violence. Secondary outcomes: schooling index, SRH index, ever married, never had sex, never pregnant, number of partners past 12 months, safe sex index, psychosocial index, Rosenberg scale and SMFQ scale (psychosocial well-being), gender equity index, attitudes towards IPV, knowledge of HIV, life skills index, health, financial literacy, knowledge of condom effectiveness, healthy intimate relationships, social capital index | ITT impacts estimated with ANCOVA models (linear regression), additional controlling for age | baseline & 24 months | After 24 months, the GE arm had no impacts on sexual or physical violence, schooling index, highest grade attained, school enrolment, increased the SRH index (b=0.229, se = 0.082) [in terms of components, decreased number of partners (b=-0.412, se=0.231), increased safe sex index (b=0.215, se=0.198)], had no impact on the psychosocial index or its components, increased the gender attitudes index (b=0.207, se=0.088) [in terms of components, no impact on gender equity index but increased attitudes towards IPV index (b=0.181, se=0.090)], increased life skills index (b=0.233, se=0.098) [in terms of components, increased HIV knowledge (b=0.198, se=0.096), increased financial literacy (b=0.216, se=0.072), increased knowledge of condom effectiveness (b=0.191, se=0.078)], and had no impact on the protective factors index. When examining components of the sexual violence index, there were no impacts on the individual items.  The GE+cash arm had no impacts on the physical or sexual violence indices, but did increase risk of sexual violence components including non-consensual touching (b=0.05, se=0.02), had no impact on schooling index or highest grade attained, increased school enrolment (b=0.043, se=0.023) , increased the SRH index (b=0.354, se = 0.087) [in terms of components, increased never married (b=0.038, se=0.014), decreased number of partners (b=-0.651, se=0.203), increased safe sex index (b=0.311, se=0.113)], had no impact on the psychosocial index or its components, increased the gender attitudes index (b=0.224, se=0.082) [in terms of components, no impact on gender equity index but increased attitudes towards IPV index (b=0.213, se=0.074)], increased life skills index (b=0.294, se=0.096) [in terms of components, increased HIV knowledge (b=0.166, se=0.081), increased financial literacy (b=0.342, se=0.080), increased knowledge of condom effectiveness (b=0.261, se=0.072)], and had no impact on the protective factors index.  The combined treatment arm (GE or GE+) had no impacts on the physical or sexual violence indices, but did increase risk of sexual violence components including non-consensual touching (b=0.04, se=0.021), increased the SRH index (b=0.229, se = 0.082) [in terms of components, increased never married (b=0.032, se=0.013), decreased number of partners (b=-0.528, se=0.194), increased safe sex index (b=0.262, se=0.087)], had no impact on the psychosocial index or its components, increased the gender attitudes index (b=0.216, se=0.077) [in terms of components, increased gender equity index (b=0.142, se=0.078) and increased attitudes towards IPV index (b=0.191, se=0.075)], increased life skills index (b=0.260, se=0.086) [in terms of components, increased HIV knowledge (b=0.176, se=0.079), increased financial literacy (b=0.280, se=0.065), increased knowledge of condom effectiveness (b=0.222, se=0.071)], and had no impact on the protective factors index. | High |
| **The SHAZ (Shaping the Health of Adolescents in Zimbabwe) Project** | | | | | | | | |
| **#** | **Author, year** | **Sample Size** | **Study Design** | **Outcomes** | **Analysis Method** | **Duration of Follow-up (from baseline)** | **Findings** | **Causal Estimate Quality** |
| 23 | Dunbar, Maternowska, Kang, et al., 2010 | 50 | Mixed-method, qualitative and quantiative (pilot at 2 sites); study not powered to estimate quantitative impacts. Used pre/post approach (no comparison group). | Quantitative: transactional sex, relationship power, economic indicators, orphan status control in sexual relationships, experience of physical or sexual violence, forced sex, tests for HIV, HSV-2, and pregnancy. Qualitative: identify factors associated with business start-up and management, and loan repayment. | **Quant:** Bivariate analysis of pre/post intervention comparisons from baseline and 6 months. **Qual:** semi-structured, open-ended interviews of 13 loan recipients to identify barriers to loan repayment; simple and rapid content analysis/ranking of factors. FGD conducted post-intervention and synthesized for life-skills improvement. | 6 months | **Quant:** Increases in had own income (from 6% to 44%, p<0.001) and had savings (from 0% to 88%; p<0.001), increased HIV knowledge (from 16% to 38%; p<0.001), increased perception of high power in non-sexual relationships (from 1% to 4% non-sexual, p=0.04). No impacts on sexual activity, condom use, transactional sex, life preferences, power in sexual relationship. **Qual:** many barriers to loan repayment - girls' vulnerability, economic shocks, insufficient financing to repay loan; risk for personal safety and goods in transport to market. Family support appeared protective in loan repayment. Lack of trust between participants and mentors. Life-skills sessions were found helpful and participants wanted more about abuse/HIV knowledge and assertiveness training. **Overall:** Ultimately, microcredit loans were not successful in this group. Empowerment findings were promising, but unintended consequences increased AGYW's vulnerability to physical/sexual abuse/coercion, underscoring hazards of microfinancing in unstable economic environments w/out appropriate social support. | Low |
| 24 | Dunbar, Kang Dufour, Lambdin, et al., 2014 | 315 (158 intervention, 157 control) | Individual-level RCT comparing full intervention to life skills and health services alone. | educational status, food insecurity, social support, experience of violence, relationship power, physical and sexual violence, rape, sexual activity, contraceptive use HIV and HSV-2, unintended pregnancy | Intent-to-treat analysis comparing outcomes within treatment arms using generalized estimating equations with an exchangeable correlation structure for repeated measures and an interaction to show moderation by treatment arm. Cox proportional hazards models to estimate impacts on HIV or HSV-2 infection, or unintended pregnancy. Robustness check on biological outcomes using inverse probability weights. | baseline; 6, 12, 18, and 24 months | Within the treatment arm, over time food insecurity decreased (OR=0.68, CI 0.60-0.77; interaction p=0.02) and received own income increased (OR=2.05, CI 1.79-2.34, interaction p=0.02). There were no effects on HIV, HSV-2, unintended pregnancy, social support, relationship power, sexual debut, sexual activity, transactional sex, physical or sexual violence, or condom use. | Low-medium |
| **Suubi** | | | | | | | | |
| **#** | **Author, year** | **Sample Size** | **Study Design** | **Outcomes** | **Analysis Method** | **Duration of Follow-up (from baseline)** | **Findings** | **Causal Estimate Quality** |
| 25 | Curley, Ssewamala, Nabunya, et al., 2016 | 157 | cRCT (school-level; n=15; 10 treatment and 5 control) | Educational planning; confidence of achieving the educational plan | Change scores were calculated by subtracting the outcome at Wave 1 from the measure at Wave 2. Independent and paired sample t-tests were conducted on the change scores. | 10-12 months post-intervention | At Wave 2 among girls, differences in change scores between treatment & control were significant for confidence in education plan (t=4.70), but were not significant for overall education plans. At wave 2 among boys, differences in change scores were significant for both education plans and confidence in education plans (t=-2.10). | Medium |
| 26 | Ssewamala, Ismayilova, McKay, et al., 2010 | n=277 (control n=142; treatment n=135) | cRCT (school-level; n=15 schools) | Attitudes toward sexual risk–taking behaviors | Implemented mixed models with school-level random effects and an interaction with treatment x time (intervention effect) and treatment x time x gender to examine moderating effects of gender. | 10 months | Treatment had a protective effect on attitudes towards sexual risk-taking behaviours (p<.05) and this was moderated by gender (p<.05), whereby intervention was protective for males but not females. | Medium |
| 27 | Ssewamala, Nielands, Waldfogel, 2012 | total n=286 (control n=148; treatment n=138) | cRCT (school-level; n=15 schools) | depressive symptoms | Implemented linear multilevel growth curve (focused on within-group changes over time) | baseline, 10 months, & 20 months | The intervention group had a significant reduction in depression over time (b=-.34, CI -.61,-.06), but the control group did not; however, the two slopes were not statistically different, (b=-.21, CI -.67, .26). Authors interpret this as a protective effect, but lack of significant differences in slopes suggest that programme effects were null. | Medium |
| 28 | Ssewamala, Brathwaite, Neilands, et al., 2023 | N=1260 adolescent girls | 3-arm cRCT randomized at the school-level (n=47 schools) | Sexual risk combined indicator for positive biomarkers for HIV, gonorrhea, trichomoniasis, chlamydia, genital warts, pregnancy; self-reported sexual risk (sexual intercourse, condom use, diagnosis of STD; intentions and attitudes towards risk-taking scale of 5 items; attitudes towards condom use; mental health (hopelessness, depressive symptoms, self-concept, self-esteem) | Comparison of differences of estimated marginal means; Multi-level logistic regression model for binary indicators and 3-level mixed effects model for continuous outcomes, all with group-by-time interaction. | 12 months, 24 months | According to differences of estimated marginal means, at 24 months, there were no impacts on biomarker-based sexual risk, self-reported sexual risk, sexual risk-taking intentions, or attitudes toward condom use. The savings arm reduced depression (β=-1.38, CI -2.63 to -0.12) and increased self-concept (β=1.96, CI 0.07 to 3.85). The combined savings and family strengthening arm reduced hoplessness (β=-0.45, CI -0.90 to -0.01) and depression (β=-2.80, CI -4.29 to -1.32) and increased self-concept (β=3.04, CI 0.295 to 5.12). According to multi-level models, after 12 months, the combined intervention arm reduced favorable attitudes towards condoms (β=5-1.05, CI -1.85 to -0.25 ), while after 24 months, the savings arm increased self-reported sexual risk (β=5.12, CI 1.58 to 16.56). Also according to multi-level models, after 12 months, the savings only arm increased self-concept (β=1.85, CI 0.28 to 3.43) and self-esteem (β=0.94, CI 0.01 to 1.87), while after 24 months, the savings only arm had no impacts, but the combined treatment reduced depressive symptoms (β=-2.04, CI -3.37 to -0.72), reduced hopelessness (β=-0.69, CI -1.16 to -0.22), increased self concept (β=2.74, CI 0.57 to 4.92) but had no effect on self-esteem. | High |
| **Suubi-Maka Project** | | | | | | | | |
| **#** | **Author, year** | **Sample Size** | **Study Design** | **Outcomes** | **Analysis Method** | **Duration of Follow-up (from baseline)** | **Findings** | **Causal Estimate Quality** |
| 29 | Jennings, Ssewamala, Nabunya, 2016 | n=346 (control n=167; intervention n=179) | cRCT at school level in two districts (Rakai and Masaka). | adolescents’ cash savings, attitudes toward savings (importance and ability to save), and attitudes toward HIV-preventive behaviors | To account for hierarchical nature of data, mixed-effects linear and logistic regression models with an interaction term of study group and time were used to examine differences in outcomes, with random effects for unmeasured school and individual-level characteristics and fixed effects for measured demographic characteristics | baseline, 12, and 24 months | Compared to the control group , intervention adolescents had higher cash savings (b = $US12.32, ±1.12, p < .001), higher savings attitudes (b=0.25 ± 0.11, p<.05) ), higher HIV-preventive attitudinal scores (b = +0.19, ±0.09, p < .05), and higher odds of a maximum HIV-prevention score (OR = 2.017, CI 1.43–2.84). | Medium |
| 30 | Karimli & Ssewamala 2015 | dyads of adolescents & caregivers: treatment n=179 dyads; control n=167 dyads | cRCT at school level (5 control schools, 5 treatment schools) | Hopelessness, self-concept, educational expectations, adolescent confidence and future orientation. | Generalized estimating equation (GEE) models to estimate population-averaged treatment effects while accounting for within-subject correlations with fixed effects for schools. | 12 months and 24 months | After 12 months, the intervention reduced hopelessness (b=-1.18, CI -2 to -0.4), increased educational expectations (b=0.54, CI 0.1 to 0.9), increased likelihood of savings (OR=2.68, CI 1.42-5.05), amount saved (b=2.69, CI 1.5-3.9). After 24 months, the intervention reduced hopelessness (b=-1.49, CI -2.2 to -0.7), increased self-concept (b=2.92, CI 0.2 to 5.6), increased confidence in educational plans (b=2.4, CI 1.3-4.2), increased likelihood of savings (OR=2.44, CI 1.25-4.79), and amount of savings (b=2.69, CI 1.5-3.9). | Medium |
| 31 | Ssewamala, Karimli, Torsten, et al., 2016 | n=346 (control n=167; intervention n=179) | cRCT (school-level; n=10 schools) | School attendance, took primary leaving exam, score on primary leaving exam (PLE; lower score is better), and self-esteem, hopelessness, self-concept, confidence in achieving educational plan | Multilevel regression (linear and logit) analyses with a random intercept with random intercept for schools. | baseline & 24 months | Intervention had a positive effect on taking PLE (OR=7.16, CI 2.03-25.29), having confidence in achieving educational plan (OR=6.06, CI 1.23-29.91), and improved test scores (b=-5.53, CI -11 to -0.1). Further, the intervention reduced hopelessness (b=-0.97, CI -1.7 to -0.3) and increased self-concept (b=1.83, CI 0.8-2.9). | Low-medium |
| 32 | Tutlam, Filiatreau, Byansi, et al., 2023 | N=346 adolescents aged 12-16 years | Two arm cRCT randomized at school-level (n=10 schools) | Child emotional and behavioral difficulties, categorized into difficulties (conduct problems, emotional symptoms, peer relationships, hyperactivity/inattention; increasing score is more difficulties) and prosocial behavior (increasing score is positive) | Multilevel mixed effects models with treat x time interaction term | 12 months, 24 months | There were no effects of the intervention on outcomes examined as measured by treat x time interaction coefficient. | Medium |
| **Suubi4Her** | | | | | | | | |
| **#** | **Author, year** | **Sample Size** | **Study Design** | **Outcomes** | **Analysis Method** | **Duration of Follow-up (from baseline)** | **Findings** | **Causal Estimate Quality** |
| 33 | Filiatreau, Tutlam, Brathwaite, et al., 2023 | N=1260 | Cluster RCT | psychosocial well-being: hopelessness (Beck’s Hopelessness Scale), self-concept (Tennessee Self-Concept Scale),and self-esteem (Rosenberg Self-Esteem Scale | Multi-level linear mixed models accounting for trial design, with fixed effects for study arm. | 12 months from enrollment | The treatment arm combining youth development accounts with mutiple family group intervention reduced hopelessness (β=-0.6, CI -1.1 to -0.1)) and increased self esteem (β=1.0, CI 0.2 to 1.8) but had no effects on self-concept. There were no effects of the youth development account only treatment arm on any outcomes. | Medium |
| **Women First and Go Girls!** | | | | | | | | |
| **#** | **Author, year** | **Sample Size** | **Study Design** | **Outcomes** | **Analysis Method** | **Duration of Follow-up (from baseline)** | **Findings** | **Causal Estimate Quality** |
| 34 | Burke, Field, González-Calvo, et al., 2019 | 5-6,000 AGWY annually in South Africa; 8,515-11,428 in Kenya | (observational) longitudinal prospective cohort using Demographic Surveillance Site (DSS) Data | HIV incidence | Poisson regression among residents aged 15 to 24 years who had at least 2 HIV test results, with the first HIV-negative test before the age of 25. Analyses compared the 5-year period immediately prior to rollout (2011 to 2015). The DREAMS scale-up period was monitored up to 3 years after DREAMS interventions were introduced (2016 to 2019). Poisson regression was used to estimate rate ratios (RRs) and 95% CIs for the effect of calendar period on HIV incidence, overall and separately by age group (age 15 to 19 years and 20 to 24 years). | 3 years | In the DREAMS implementation phase (2016 to 2018), age-adjusted RR (aRR) for HIV incidence were 0.62 (CI 0.48 to 0.82]) among 15-19 year olds as compared to the time period 2011-2015 in South Africa. Incidence estimates were not statistically significant among 20- to 24-year-olds in South Africa, nor among any age group in Kenya. | Low |
| **Women of Worth (cash plus)** | | | | | | | | |
| **#** | **Author, year** | **Sample Size** | **Study Design** | **Outcomes** | **Analysis Method** | **Duration of Follow-up (from baseline)** | **Findings** | **Causal Estimate Quality** |
| 35 | Naledi, Little, Pike,  et al., 2022 | n=5116 | Pre/post design combining participants from pilot and post-modified phase. RCT (individual-level) assignment to care only or cash plus care; however due to attrition in the care only group, authors pooled estimates for cash and cash plus care and examined changes over time (pre/post). Low retention rates (pilot phase: 4% in care arm, 43% in cash + care; modified phase: 6% in care arm; 77% in cash + care arm) | HIV testing, condom use, HIV risk perception, contraceptive use, treatment for STI, GBV threat, forced sex, transactional sex, employment, family satisfaction. | Logistic regression with subject-level random effects. Models were adjusted for characteristics not balanced at baseline and those associated with lost to follow-up at endline. Compare changes over time (pre/post) in the combined treatment groups (cash and cash plus care) | baseline, post-11 session, & 6-30 months post-intervention | Immediately after the programme ended, the intervention was associated with reduced HIV testing (OR=0.28, CI 0.20-0.31), reduced condom use at last sex (OR=0.49, CI 0.40-0.60), reduced high HIV risk perception (OR=0.05, CI 0.03-0.08), increased contraceptive use (OR=1.62, CI 1.29-2.03), increased the odds of being treated for an STI in the last 6 months (OR=1.50, CI 1.21-1.85), reduced GBV threat (OR=0.53, CI 0.41-0.69), reduced forced sex (OR=0.37, CI 0.37-0.66), reduced transactional sex (OR=0.50, CI 0.37-0.66), increased probability of being employed (OR=3.34, CI 2.22-5.04), and increased family satisfaction (OR 1.45, CI 1.20-1.75). At the post-intervention follow-up wave, impacts were no longer significant, with the exception of being employed, which was still positive (OR=2.47, CI 1.69-3.59). | Low |
| **Ujana Salama: Cash Plus Model for Safe Transitions to a Healthy and Productive Adulthood** | | | | | | | | |
| **#** | **Author, year** | **Sample Size** | **Study Design** | **Outcomes** | **Analysis Method** | **Duration of Follow-up (from baseline)** | **Findings** | **Causal Estimate Quality** |
| 36 | Chzhen, Prencipe, Eataama, et al., 2021 | 2458 | cRCT | gender equitable attitudes using GEM scale (overall and sub-scales) | Estimated intent-to-treat (ITT) effects using ANCOVA models at midline and endline. | Midline (12 months) and then 24-month follow-up (endline) | In the pooled (male and female) sample, the intervention increased gender equitable attitudes on the overall GEM scale at midline (b=0.88, se=0.40) but not at endline. The intervention increased attitudes measured by the domestic chores subscale at midline (b=0.27, se=0.10) and endline (b=0.23, se=0.07). In analyses stratified by gender, the intervention increased the overall GEM scale among males at midline (b=1.52, se=0.52) and increased attitudes as per the violence subscale among males at midline (b=0.30, se=0.12) and endline (b=0.24, se=0.11), increased the sexual relationships subscale among males at midline (b=0.43, se=0.18), and increased the domestic chores subscale among males at midline (b=0.48, se=0.21)) and endline (b=0.28, se=0.09). There were no statistically significant impacts on gender equitable attitudes among females. | High |
| 37 | Palermo, Prencipe and Kajula, 2021 | n=904 | cRCT (village-level; n=130) | Primary outcomes: emotional violence, physical violence, sexual violence, perpetration of physical violence, perpetration of emotional violence, help-seeking for violence; secondary outcomes: age at first sex, age-disparate sex, locus of control, self-esteem, self-perceived stress, school attendance, married/cohabiting, economic activities, domestic chores | ITT impacts estimated with OLS models and linear probability models for binary outcomes, controlling for age, sex, and stratum  (district and village size) | 24-26 months from baseline | Intervention reduced sexual violence experiences by 3pp (CI -0.06, -0.00) in the pooled sample; when examined by gender, intervention resulted in a 5pp reduction of sexual violence among females (CI -0.10, -0.00) and there were no impacts among males. In the pooled and female samples, there were no impacts on perpetration, but among males, the intervention reduced physical violence perpetration by 6pp (CI -0.10, -0.02). There were no impacts on physical and emotional violence, help-seeking, nor emotional violence perpetration. Among secondary outcomes in the pooled sample, the intervention increased livestock tending by 9pp (CI 0.02-0.17) and self-esteem (b=0.19; CI 0.08, 0.29) but had no impacts on age at first intercourse, age disparate sex, self-efficacy, self-perceived stress school attendance, married/cohabiting, paid work outside household, any economic activities, farm work for household, or domestic chores. Among females, the intervention increased livestock herding by 14 pp (CI 0.03, 0.24) and self-esteem (b=0.19, CI 0.05, 0.33) and sped up the age at first intercourse (b=0.55, CI -1.02, -0.09) and reduced the probability of school attendance by 10pp (CI 0.19, 0.00). Among males, the intervention increased self-esteem (b=0.29, CI 0.05,0.36). There were no impacts on age-disparate sex, locus of control, self-perceived stress, married/cohabiting, paid work outside household, any economic activities, farm work for household, or domestic chores among males or females in stratified analyses. | High |
| 38 | Prencipe, Houweling, van Lenthe, et al., 2022 | N=2458 | 2-arm cRCT (65 treatment, 65 control) | Depressive symptoms measured with CES-D, engagement in school and paid work jointly, school attendance, engagement in exclusive paid work, self-esteem, locus of control, quality of life, and having a romantic partner. | Intent-to-treat effects estimated with inear mixed models with village-level random effects and controlled for age, sex, baseline values of outcomes, and sampling strata. | Approximately 1 year and 2 years | After 2 years, the intervention reduced the odds of depressive symptoms (OR=0.67, CI 0.52 to 0.86) as measured by the binary indicator but had no effects on the continuous measure among the full sample. However, quantile regression estimates showed significant reductions in symptoms among those with more depressive symptoms at baseline. The intervention reduced the odds of engaging jointly in education and paid work (OR=0.46, CI 0.24 to 0.87), increased the odds of being engaged exclusively in paid work (OR=1.28, CI 1 to 1.64), and increased self esteem (Risk difference = 0.10, CI 0.03 to 0.18). There were no effects on quality of life, locus of control, school attendance, or having a romantic partner. | High |
| 39 | Ranganathan, Quinones, Palermo et al., 2022 | N=864 unmarried adolescent girls and young women | Mixed methods, 2-arm cRCT (65 treatment, 65 control) and qualitative interviews | Engagement in transactional sex | ANCOVA models, controlling for outcome at baseline, age, and sampling strata. | 2 years | No impacts of intervention on transactional sex. | High |
| 40 | Waidler, Gilbert, Mulokozi, et al., 2022 | n=1993 | cRCT (village-level; n=130) | ever married, has girlfriend/boyfriend, ever had sex, age at first intercourse, number of sexual partners 12 months, had concurrent sexual relationships past 12 months, ever pregnant, currently pregnant, ever got a female pregnant (males) | ITT impacts estimated with ANCOVA models controlling for gender, age at baseline and district-size dummies. | 24-26 months | In pooled (male and female) sample, the intervention increased contraceptive knowledge at Round 2 (b=0.039, se=0.02) and Round 3 (b=0.045, se=0.02), increased HIV knowledge at Round 2 (b=0.052, se=0.03), increased Probability of HIV testing by 6.8pp (se=0.03) at Wave 3, increased probability of knowing where to get contraceptives at Wave 2 (4.3pp, se=0.02) and Wave 3 (1.9pp, se=0.01), and increased probability of knowing where to get condoms at Wave 2 (3.5pp, se=0.02) and Wave 3 (2.5pp, se=0.01). There were no impacts on ever married, had girlfriend/boyfriend, ever had sex, age first intercourse, number of sexual partners, had concurrent relationship, ever pregnant, currently pregnant, ever got a female pregnant at either round, condom use. Among females, the intervention increased contraceptive knowledge at Round 2 (b=0.062, se=0.02), reduced age at first intercourse by approximately 5.7 months (β=−0.476; se=0.15; 12 months×0.476=5.7 months) at Round 3. Among males, the intervention reduced the probability of never being married (b=-0.028, se=0.01), increased the probability of HIV testing by 8.5pp (se=0.03), increased knowledge that condom use reduces HIV risk by 8.8pp (se=0.03), increased probability of visiting a health facility by 5.6pp (se=0.02), increased the probability of knowing where to seek contraception by 3.1pp (se=0.01). | High |
| **Unnamed Intervention** | | | | | | | | |
| **#** | **Author, year** | **Sample Size** | **Study Design** | **Outcomes** | **Analysis Method** | **Duration of Follow-up (from baseline)** | **Findings** | **Causal Estimate Quality** |
| 41 | Austrian & Muthengi, 2014 | 1064 | (quasi-experimental) case-control study | Touched indecently in the last 6 months, teased by people of opposite sex, has plan for saving money, has a budget, knowledge of reasons for saving, saved using informal method only | Random-intercept logistic regression with interaction term between treatment groups and time periods | 12 months | As compared to the no intervention group, Savings only increased the odds of having been sexually touched (OR = 3.146; CI 1.397-7.082); teased by men (OR = 1.962; CI 1.088-3.540); having a budget (OR=2.442, CI 1.448-3.121); saving money in last 6 months (OR=1.691, CI ) and lowered the odds of knowledge of reasons for saving (OR=0.442, CI 0.250-0.783). Savings Plus had no effects on being touched indecently or teased by men but increased likelihood of having a budget (OR=2.318, CI 1.441-3.729); saving in past 6 months (OR=2.125, CI 1.336-3.381); increased HIV transmission knowledge (OR=3.412, CI 1.703-6.834); knowledge of contraception (OR=2.609, CI 1.485-4.587); and knowledge of HIV prevention methods (OR=4.156, OR 1.891-9.132) and lowered the odds of saving using informal method only (OR=0.006, CI 0.001-0.050). | Medium |
| 42 | Hegdahl, Musonda, Svanemyr, et al., 2022 | N=4922 girls in 157 schools | Cluster RCT at school-level with three arms: 1) economic support arm (n=63 clusters), 2) combined intervention arm (economic support + community and parent meetings; n=63 clusters, and 3) control arm (n=31 clusters) | sexual activity, contraceptive use and beliefs | Pairwise comparisons using generalized estimating equations | Interviewed twice per year after implementation | Both intervention arms reduced the risk of adolescent girls being sexually active in the last 4 weeks (economic RR=0.70, CI0.54 to 0.91; combined RR=0.59, CI 0.46 to 0.75). The combined intervention arm reduced the risk of unprotected sex (RR=0.53, CI 0.37 to 0.75). The intervention did not have any effects on recent contraceptive use, current contraceptive use, knowledge of contraceptive methods, perceived norms on contraceptive use, or high perceived control around obtaining contraceptives and condom use. | High |
| 43 | Tozan, Capasso, Sun, et al., 2019 | n=1,383 | cRCT (school-level; n=48 randomized to three study arms (control, Bridges, Bridges+)) | self-rated health, depression, hopelessness, self-concept, self-efficacy, sexual risk-taking intentions, HIV prevention attitudes, and HIV knowledge | Intent-to-treat analyses using multilevel models. Incremental cost-effectiveness ratios (ICERs) calculated. | baseline & 48 months | At 48-months, BridgesPLUS increased self-rated health, (b=0.25, 95% CI 0.06, 0.43), HIV knowledge (b=0.21, 95% CI 0.01, 0.41), self-concept (b=0.26, 95% CI 0.09, 0.44), and self-efficacy (b=0.26, 95% CI 0.09, 0.43) and lowered hopelessness (b=-0.28, 95% CI -0.43, -0.12) but had no effects on depression, sexual risk taking, or HIV prevention attitudes; whereas Bridges improved self-rated health (b=0.26, 95% CI 0.08, 0.43) and HIV knowledge (b=0.22, 95% CI 0.05, 0.39) but had no effects on depression, hopelessness, self-concept, self-efficacy, sexual risk taking, or HIV prevention attitudes. ICERs ranged from $224 for hopelessness to $298 for HIV knowledge per 0.2 standard deviation change. | Medium |
