# Supplementary material for: Systematic review of cash plus or bundled interventions targeting adolescents in Africa to reduce HIV risk
Source: BMC Public Health. 2024 Jan 20;24:239. doi: 10.1186/s12889-023-17565-9 (PMC10799364; doi:10.1186/s12889-023-17565-9)
Supplement: Supplementary file 5 — Additional file 5: Appendix 5. Sum of Findings (Qualitative Studies). [file 12889_2023_17565_MOESM5_ESM.docx]

| **Appendix 5. Sum of Findings *(Qualitative Studies)*** | | | | | | | | |
| --- | --- | --- | --- | --- | --- | --- | --- | --- |
|  | | | | | | | | |
| **DREAMS** | | | | | | | | |
| **#** | **Author, year** | **Sample Size** | **Study Design** | **Outcomes** | **Analysis Methods** | **Duration of follow-up (from baseline)** | **Findings** | **Causal Estimate Quality** |
| 14 | Chimwaza-Manda, Kamndaya, Pilgrim, et al., 2023 | N=43 (n=23 in GOC and n=20 not in GOC) | Descriptive comparative qualitative study | Assessed social support concerning sexual behaviors for girls belonging to Girls Only Clubs and those who were not club members | Conducted in-depth interviews with participants. Used constant comparative analysis where systematic procedures are used to assign codes and categorise data, subsequently identifying themes/patterns. This provides a logical approach to examining qualitative data when comparing different groups (GOC and non-GOC members). | N/A | Both club and non-club participants found information social support concerning sexual relationships from parents, older relatives, and friends. However, they differed where club participants received detailed sexual health information from clubs: members consulted others on decision-making and information on sexual relationships; used condom (due to education received from the clubs) and quit sexual relationships; corrected sexual misinformation among peers with information learned from the club. Club members also appeared to receive more social support, making them more knowledgeable and better at handling sexual relationship issues than those not in clubs. Interventions that integrate social support, including asset building and safe spaces, are important SRH programming for very young adolescents. | N/A |
| 4 | Gangaramany, Balvanz, Gichane, et al., 2021 | Phase 1 n=61; Phase 2 n=120 | Qualitative: in-depth interviews (Phase 1) and simulated scenarios (through EthnoLab, a decision-making game) with qualitative follow-up questions to understand how decisions were made. | sexual, relationship, and financial decisions and decision-making processes, as well as the impact of the CT program upon AGYW. | Codebook was developed reflecting question domains, and including concepts of emotional appraisal for decision-making (relevance, implications, coping potential, and social norm significance) derived from the Emotional Appraisal Framework. Transcripts were coded manually. Key observations and insights were grouped and summarized for each code. | 18 months | Through phases one and two of research, identified three key themes in AGYW’s vision of their desired future economic state: 1) positive social image, 2) power balance and respect, and 3) emotional and economic security. It was found that AGYW lacked a vision to build self-agency. CT had some positive impacts on AGYW: helped to meet basic needs, reduced financial dependency on men, helped to resist influence of peers in terms of money spending and engagement in transactional sex. CT and WORTH+ engaged YW in business who would not have been otherwise. Findings suggest that providing economic resources to AGYW through CT has short-term benefits, but without ensuring self-agency, it is unlikely to be an effective long-term intervention for economic empowerment. | N/A |
| 5 | Gichane, Wamoyi, Atkins, et al., 2020 | Total N=80; n=20 longitudinal interviews, n=40 cross sectional in-depth interviews, and n=20 narrative timeline interviews | Qualitative: conducted longitudinal, cross-sectional, and narrative timeline in-depth interviews. | transactional sex and partner selection criteria | Analyzed data using a thematic approach guided by the transactional sex framework. To improve the confirmability and dependability of the data, analyses were conducted in a collaborative and iterative fashion. | Baseline interviews in June 2017, with follow-up interviews in June 2018 | Cash transfers may potentially reduce engagement in transactional sex that is motivated by economic vulnerability. Participants stated economic support as an important component of nearly all of the transactional relationships described, but there was a spectrum of relationships, ranging from those where money and sex were strongly connected, to those where they were less connected. Emotional connection, as well as young women's socioeconomic status, played a role in where the relationships fell on the spectrum. Relationships that demonstrated strong emotional connections saw a less-pronounced association between sex and money. Relationships where the primary reason for starting and/or maintaining them was because of the partner’s financial support had a strong association between sex and money, where sex was conditional on the transaction. | N/A |
| 7 | Manda, Pilgrim, Kamndaya, et al., 2021 | N=23 | Qualitative: in-depth interviews | experiences of VYA girls on DREAMS’ Go Girl club participation, SRH knowledge to reduce their risk for HIV, negative sexual health outcomes | Narrative inquiry & thematic analysis | 24 months | **Participation in clubs:** Girls generally enjoyed the go girl clubs, and joined primarily to learn about SRH, education, and child abuse. There were relatively few barriers to club participation. Out of school girls joined to have something to do with their time. **Gender norms/roles:** Participants reported learning about gender roles, and bringing that knowledge to their families to shift gendered roles in household chores. **Life skills/social networks:** participants reported better social connection and use of their time due to life skills learned at the clubs. **Education:** participants who indicated they had dropped out of school due to poverty found that association of the girls’ clubs with Village Savings Loans helped their families/caregivers to meet basic needs and send the girls back to school. **SRH knowledge:** the majority of participants reported learning about SRH (menstruation, STIs, HIV, contraception) for the first time at the girls' clubs, and noted this is a critical component of their participation in the clubs. Participants reported linkage to HIV testing and health services appointments. | N/A |
| 9 | Pettifor, Wamoyi, Balvanz, et al., 2019 | total N=80 (IDIs n=60; narrative interviews n=20) | Qualitative: in-depth and narrative interviews | partner choice and transactional sex | Applied thematic analysis to analyse data (a systematic, inductive process of sorting content by codes and conveying the meaning through themes) | 12 and 24 months from baseline | Participants in a cash transfer plus programme discussed how HIV risk could be reduced through decreased dependence on male sex partners. Two main mechanisms were identified: 1) Young women reported that the cash transfers allowed them to obtain basic needs (e.g., food, toiletries), which helped to reduce their dependence on male sex partners for these goods through activities like transactional sex. (This experience was reported more among poorer participants.) 2) Young women reported that the financial education/business development aspect of the programme supported skills that helped them to refuse some male sex partners. This was more pronounced in unmarried women than married women. Social support (from both family members and programme mentors) appeared to increase young women's ability to start businesses and gain income, thus making them less dependent on male sex partners. Overall, the CT component appeared to have reduced AGYW engagement in transactional sex use to meet basic needs. The financial education/business development and mentorship component appeared to support AGYW agency and promote self-esteem and future orientation, which may help AGYW refuse some male sex partners, thus helping to reduce their engagement in transactional sex. | N/A |
| 12 | Wamoyi, Balvanz, Atkins, et al., 2020 | N=80 | Qualitative: longitudinal IDIs (n=20), cross-sectional IDIs (n=40), and narrative timeline interviews (n=20) | sexual decision making | Using thematic analysis, conducted narrative timeline interviews wd to understand how AGYW’s sexual behavior and relationships changed over time, and what influence the CT had on that change. | 3 months & 1 year from baseline | AGYW established the concept of empowerment as “independence” and “hope and aspiration." Three pathways were identified through which CTs were thought to empower AGYW: 1) hope and aspiration for a better future, 2) access to knowledge, 3) potential for economic gain. AGYW reported reduced in engagement in transactional sex, experiences of intimate partner violence, and sexual risk-taking as a result of empowerment. The sense of responsibility that also developed as a result of economic empowerment helped to increase participants' self-esteem and decision-making confidence, which decreased AGYW’s participation in sexual risk behaviors. | N/A |
| 13 | Wamoyi, Balvanz, Gichane, et al., 2020 | N=80 | Qualitative: longitudinal IDIs (n=20), cross-sectional IDIs (n=40), and narrative timeline interviews (n=20) | use of cash, changes in spending over time, and decision making on use of cash | Applied thematic analysis, created matrices summarising major topical areas. | 3 months & 1 year from baseline | The analysis revealed 5 categories where AGYW used their CTs: 1) business development, 2) survival, 3) self-care, 4) helping their family, and 5) savings. The primary uses of CT funds were investment in businesses and livestock for saving, but the ways in which AGYW used the cash changed over instalments. When determining how best to use the CTs, AGYW discussed options with a variety of consultants, the most common being their mothers, programme personnel, and long-term partners or husbands. Overall findings indicate that CT programmes that incorporate both entrepreneurial mentorship, as well as cash transfers given directly to AGYW, could have implications for HIV prevention, SRH, and social and economic development. | N/A |
| **Girls Empowerment Programme (GEP)** | | | | | | | | |
| **#** | **Author, year** | **Sample Size** | **Study Design** | **Outcomes** | **Analysis Methods** | **Duration of follow-up (from baseline)** | **Findings** | **Causal Estimate Quality** |
| 2 | Berry, Kuriansky, Little, et al., 2013 | N=40 | **Quantitative:** observational pre/post tests **Qualitative:** anecdotal reports | Income-generating activities knowledge | Mean differences reported (no statistical tests) | 1 week | increase in income-generating activity knowledge from 40.6% to 71.1% (for a lot or great deal of knowledge). | Low |
| **Research Initiative to Support the Empowerment of Girls (RISE)** | | | | | | | | |
| **#** | **Author, year** | **Sample Size** | **Study Design** | **Outcomes** | **Analysis Methods** | **Duration of follow-up (from baseline)** | **Findings** | **Causal Estimate Quality** |
| 8 | Milimo, Zulu, Svanemyr, et al., 2021 | N=48 | Qualitative arm of RCT "RISE"; used 18 in-depth interviews and 4 focus groups | Education and sexual decision making | Thematic analysis | 24 months (Sept 2016-Nov 2018) | Findings suggested several benefits of the economic support for the female adolescents such as economic independence and empowerment; increased assertiveness and autonomy; reduced desire for sexual relationships with boys in exchange for cash and gifts; increased motivation for school; enhanced parental and community support for female adolescents’ education and; reduced school dropouts. However, they also experienced jealousy from those who did not benefit from the economic support. Economic support played a significant role in influencing both educational and sexual decision making among female adolescents. | N/A |
| **Women First and Go Girls!** | | | | | | | | |
| **#** | **Author, year** | **Sample Size** | **Study Design** | **Outcomes** | **Analysis Methods** | **Duration of follow-up (from baseline)** | **Findings** | **Causal Estimate Quality** |
| 3 | Burke, Packer, González-Calvo, et al., 2019 | n=49 girls, n=24 household heads, n=36 influential males, n=118 community members | (QUALITATIVE) longitudinal mixed-methods evaluation-this portion is the qualitative interviews (initial part was quantitative survey) | Respondent characteristics, implementation and sustainability of business componenet, sexual behaviors (inc. HIV risk factors), school attendance | Applied the social ecological model (SEM) to the design of the qualitative component of the evaluation, and to the analysis of the data. Developed initial codebooks for each respondent type and data collection round based on topic interview guides. Then analysts coded the interviews. Then generated coding reports and summarized data accoring to SEM level and other themes | Two interviews: first interview was after intervention was completed; second 1 year after first interview | **Business component:** majority of girls earned money and were satisfied with the amount earned. At round 1, just under 1/4 of participants stated they did not earn sufficient money. At round 2, roughly half were earning money, primarily due to the fact that they used earned money to repay the intervention for business-related items.  **Sexual behaviors:** Overall, attitudes towards girls’ sexual activity were negative. Girls reported that they learned premarital sex was "wrong" and that girls who engaged in it garnered little respect during the intervention. Respondents also reported that girls became more “respectful” due to the intervention. In Round 1, respondents in 11 out of the 12 communities reported reduced engagement for girls in transactional and/or intergenerational sex, due to money earned through the intervention. Positive effects on girls’ sexual risk behaviors were sustained for girls who maintained their businesses and income. Girls reported increased knowledge of GBV. Half of girls reported they were attending school and placed higher value on their education because of the intervention. At round 2, some respondents stated that girls from the intervention re-engaged in transactional sex or had multiple partners out of financial need; several girls reported getting married in the previous year due to financial pressure or because of pregnancy. Increased knowledge of GBV was sustained, and approximately two-thirds of the respondents described reduced perpetration of sexual or physical violence against women or girls in the community. One-third of girls said they were still in school. An additional third stated that they had dropped out of school within the year since the interventing ended. In both rounds, girls reported wanting or intending to use condoms/other family planning methods in the future; however, fewer reported actually using condoms. At round 1, most participants were single and childless. By round 2, some were married and had children. | N/A |
| 6 | Lenzi, Packer, Ridgeway, et al., 2019 | Round 1 N=121; Round 2 N=119 | Qualitative study: conducted in-depth interviews and foguc group discussions | adolescent girls’ risk of HIV and gender-based violence, school attendance, girls empowerment. Sought to understand perceptions of Women First at the interpersonal, household, and individual levels. | Review of summarised data from Rounds 1 and 2 to understand patterns how respondents understand respectful behaviour with the intervention followed by inductive analysis of responses focusing on respect and how respondents describe a girl who is well-behaved. Iterative process to sort and order data, then into broader thematic domains that captured related behaviours. Data were compared across respondent types and communities. | First round in 2015 when program ended; second round 12 months later | Respondents described “good girls” as deferential and obedient; productive and willing to serve their families and communities; and sexually chaste and modestly dressed. Respondents believed the intervention had reinforced or taught these behaviours, although they were generally aligned with gender norms that were not part of the formal intervention content and sometimes contrary to the intervention’s primary goals. | N/A |
| **Unnamed Intervention** | | | | | | | | |
| **#** | **Author, year** | **Sample Size** | **Study Design** | **Outcomes** | **Analysis Methods** | **Duration of follow-up (from baseline)** | **Findings** | **Causal Estimate Quality** |
| 1 | Banda, Svanemyr, Sandøy, et al., 2019 | N=46 | Exploratory qualitative design (using participants from RISE trial in Zambia): 5 focus group discussions and 6 in-depth, semi-structured interviews analysed with thematic analysis. 6 participants in individual interviews and 40 in 5 focus groups: one for non-beneficiary boys in the same class with beneficiary girls, one mixed group of beneficiary girls and non-beneficiary boys in same class, one for beneficiary parents, one for parents of non-beneficiary boys from same class as beneficiary girls and one for beneficiary girls. Individual interviews participants included community gate keepers – two village headmen, two teachers, one PTA Chair and one community member. | factors influencing acceptability of economic support in RISE trial - advantage, compatibility, observability, complexity | A priori coding and thematic network analysis using Roger's perceived attributes framework to understand acceptability of intervention. | 4 years | Findings organized around 4 of Rogers' 5 attributes: 1) relative advantage in that econ support was accepted due to perceived wide benefits to include other members of the family; 2) Compatibility in that econ support focused on needs and financial challenges concerning education and school fees; comparability with key community values and interests in that the econ support was consistent with the target populations values and would mold the same in participating girls (and promoted decrease in transactional sex) but boys' families felt left out. There was fear that girls would get used to having money and look for it through transactional sex after intervention completed; 3) observability of increased school attendance among girls and decrease teen pregnancy and marriage incidence (girls were more focused on school instead of how to get money for sex); 4) Complexity in that RISE was easy to understand and access (due to intensive community education). Findings suggest that contextual factors and norms play a large role in acceptability of intervention, and future success of interventions requires ease of access to and understanding of the intervention components within the community. | N/A |
| 15 | Mason, Zulaika, van Eijk, et al., 2022 | N=231 (n=79 at baseline; n=77 at midline; n=75 at endline) | Qualitative analysis of participants in a 4-arm cRCT | causes of school dropout, drivers of absenteeism and nature of relationships with males (including transactional sex, multiple partners and consequences of pregnancy) | Used both deductive and inductive approach to thematic analysis to analyse data from 24 semi-structured focus group discussions. (8 focus groups per time point - baseline, midline, and endline. Did not divide focus group participants by study arm; aimed for 8-12 participants/group) | 1 year and 2 years post-intervention | **4 arms:** 1) menstrual cup only group; 2) cash transfer only group; 3) menstrual cup + cash transfer; 4) control (puberty + hygiene education). All groups received puberty + hygiene education. **Baseline:** no differences. **Midline & Endline:** cash transfer only and menstrual cup + cash transfer: similar results, with girls in those groups reporting fewer pregnancies and less school drop outs, due to the cash component reducing their need to engage in transactional sex. These girls also reported feeling empowered to refuse men's sexual advances. Menstrual cup only group reported less absenteeism due to menstrual bleeding, but no effect on drop out or pregnancy. Control and menstrual cup only groups reported feelings of empowerment, but did not see any behavioral change related to dropout or pregnancy. Future programmes should consider alleviating material deprivation (which prevents girls from attending or performing at school) through cash transfers in combination with hygiene and education packages. | N/A |
| 10 | Sitienei & Pillay, 2019 | N=12 (females n=7; males n=5) | Qualitative: in-depth interviews, focus group discussions, and autobiographies | experiences of psychological, education and social-support interventions. | Data were coded and organized by themes and then triangulated between individual interviews and focus groups | 24 months | Respondents reported receiving psychosocial support from mentors and that peer-groups provided an opportunity to share with peers who had similar experiences and receive emotional support. Respondents felt gratitude for receipt of school uniforms, food, and house rentals. Respondents already reported learning life skills. | N/A |
| 11 | Skovdal, 2010 | total N=27 (children n=21; caregivers n=6) | Qualitative: participant workshops and in-depth interviews | Explores how intra-community relations can both facilitate and undermine child-led microfinance activities, and how these activities in turn can further strengthen some intra-community relations | Workshops, written narratives, and in-depth interviews were coded thematically through an iterative process to examined how intra-community relations impact outcomes of interest | 10 months | Thematic analysis indicated that the children leaned on both the guardians involved in the project, as well as on the their peers, with reliance on their peers in particular helping to strengthen their coping capabilities. The children's disenfranchised position in the community meant that they were vulnerable to being taken advantage of by some adults, who used the activities for personal gain. Additionally, children who were less engaged in collective work undermined the morale of their more active peers. Overall, however, as participation in the project increased respect for participant from the community, both caretakers and children began to view the caregiving of children differently, which facilitated a change in caregiver/child relations. The paper concludes that microfinance interventions targeting children and young people must consider children’s relationships with each other as well as with adults as key determinants of project success. | N/A |
