# Supplementary material for: Systematic review of cash plus or bundled interventions targeting adolescents in Africa to reduce HIV risk
Source: BMC Public Health. 2024 Jan 20;24:239. doi: 10.1186/s12889-023-17565-9 (PMC10799364; doi:10.1186/s12889-023-17565-9)
Supplement: Supplementary file 6 — Additional file 6: Appendix 6. Sexual Risk Behaviour Outcomes Sub-Chart. [file 12889_2023_17565_MOESM6_ESM.docx]

| **Appendix 6. Sexual Risk Behaviour Outcomes Sub-Chart** | | | | | |
| --- | --- | --- | --- | --- | --- |
| ***Panel A: quantitative studies*** | | | | | |
| **Paper** | **Sexual Risk Behavior** | | | | |
|  | Condom use | Age-disparate  partnerships | | Transactional sex | Concurrency/Number of Sexual Partners |
| Austrian, Soler-Hampejsek, Behrman, et al., 2020 |  |  | |  |  |
| Austrian, Soler-Hampejsek, Kangwana, et al., 2021 |  |  | |  |  |
| Bandiera, Buehren, Burgess, et al., 2020 |  |  | |  |  |
| Chabata, Hensen, Chiyaka, et al., 2021 |  |  | |  |  |
| Dunbar, Maternowska, Kang, et al., 2010 | NS |  | |  |  |
| Dunbar, Kang Dufour, Lambdin, et al., 2014 | NS |  | | NS |  |
| Floyd, Mulwa, Magut, et al., 2022 |  |  | | NS |  |
| Govender, Beckett, Reddy, et al., 2022 |  | NS | |  |  |
| Hegdahl, Musonda, Svanemyr, et al., 2022 |  |  | |  |  |
| Kuringe, Christensen, Materu, et al., 2022 | NS | NS | | NS | NS |
| Mathur, Heck, Patel, et al. 2022 |  |  | |  |  |
| Naledi, Little, Pike, et al., 2022 |  |  | |  |  |
| Özler, Hallman, Guimond, et al., 2020 |  |  | |  |  |
| Palermo, Prencipe and Kajula, 2021 |  | NS | |  |  |
| Ranganathan, Quinones, Palermo, et al., 2022 |  |  | | NS |  |
| Ssewamala, Brathwaite, & Neilands, 2023 |  |  | |  |  |
| Waidler, Gilbert, Mulokozi, et al., 2022 |  |  | |  | NS |
| = Protective effects  = Mixed protective effects | | | = Adverse effects  = Mixed adverse effects | | |
