# Supplementary material for: Systematic review of cash plus or bundled interventions targeting adolescents in Africa to reduce HIV risk
Source: BMC Public Health. 2024 Jan 20;24:239. doi: 10.1186/s12889-023-17565-9 (PMC10799364; doi:10.1186/s12889-023-17565-9)
Supplement: Supplementary file 7 — Additional file 7: Appendix 7. List of Abbreviations. [file 12889_2023_17565_MOESM7_ESM.docx]

**Appendix 7. List of Abbreviations**

AIDS = acquired immunodeficiency syndrome

AGYW = adolescent girls and young women

ALWHIV = adolescents living with HIV

AYA = adolescents and young adults

BCC = behaviour change communication

CI=confidence interval

CT=cash transfer

DSS = demographic surveillance site

EAG = early adolescent girls

FGD = focus group discussion

GBV = gender-based violence

HH = head of household

HIV = human immunodeficiency virus

IDI = in depth interview

IPV = intimate partner violence

MSM = men who have sex with men

OVC = orphans and vulnerable children

PICO = population, intervention, comparative intervention, outcome (search protocol)

PEP = post-exposure prophylaxis

PrEP = pre-exposure prophylaxis

RCT = randomized control trial

cRCT = cluster randomized control trial

SE=standard error

SSA = sub-Saharan Africa

UN = United Nations

UNICEF = United Nations International Children’s Emergency Fund

VYA = very young adolescents

YWSS = young women who sell sex
